# Fake news during the war in Ukraine: coping strategies and fear of war in the general population of Romania and in aid workers

**DOI:** 10.3389/fpsyg.2023.1151794

**Published:** 2023-05-12

**Authors:** Mona Vintilă, Gianina-Mălina Lăzărescu, Argyroula Kalaitzaki, Otilia Ioana Tudorel, Cosmin Goian

**Affiliations:** ^1^Faculty of Sociology and Psychology, West University of Timișoara, Timișoara, Romania; ^2^Department of Social Work, Faculty of Health Sciences, Hellenic Mediterranean University, Heraklion, Greece

**Keywords:** war refugees, asylum seekers, mental health workers, health providers, demoralization, fake news overload

## Abstract

**Introduction:**

In addition to the health crisis that erupted during the COVID-19 pandemic, the war between Russia and Ukraine is impacting the mental health and wellbeing of the Romanian population in a negative way.

**Objectives:**

This study sets out to investigate the impact that social media consumption and an overload of information related to the armed conflict between Russia and Ukraine is having on the distribution of fake news among Romanians. In addition, it explores the way in which several psychological features, including resilience, general health, perceived stress, coping strategies, and fear of war, change as a function of exposure to traumatic events or interaction with victims of war.

**Methods:**

Participants (*N* = 633) completed the General Health Questionnaire (GHQ), the CERQ scale with its nine subscales, the Perceived Stress Scale (PSS), and the BRS scale (Brief Resilience Scale), the last of which measures resilience. Information overload, information strain and the likelihood of the person concerned spreading fake news were assessed by adapting items related to these variables.

**Findings:**

Our results suggest that information strain partially moderates the relationship between information overload and the tendency to spread false information. Also, they indicate that information strain partially moderates the relationship between time spent online and the tendency to spread false information. Furthermore, our findings imply that there are differences of high and moderate significance between those who worked with refugees and those who did not as regards fear of war and coping strategies. We found no practical differences between the two groups as regards general health, level of resilience and perceived stress.

**Conclusion and recommendations:**

The importance of discovering the reasons why people share false information is discussed, as is the need to adopt strategies to combat this behavior, including infographics and games designed to teach people how to detect fake news. At the same time, aid workers need to be further supported to maintain a high level of psychological wellbeing.

## Introduction

In recent years, the entire population of Romania has been exposed to various disasters, for example the COVID-19 pandemic—events that have had a major impact on both physical and mental health. Most recently, we have been exposed to a large-scale conflict close at hand, the war between Russia and Ukraine, which has created a new context of uncertainty and panic among people. Coming on top of the health crisis that erupted during the COVID-19 pandemic, the war between the two states is once again impacting, again in a negative way, the mental health and wellbeing of the population. A number of states, among them Romania, Hungary, and Poland, have mobilized themselves to help those who have left their country in the hope of escaping with their lives.

One of the most important aspects to consider when discussing war victims is the concept of demoralization. This has been defined as the inability of individuals to cope with stressful events and is associated with a lack of hope and meaning in life, helplessness, and low self-esteem (Clarke and Kissane, 2002). This feature was observed first among American soldiers during World Word II and subsequently among Holocaust survivors, immigrants, mental health providers, and patients suffering from psychiatric or somatic symptoms, and is associated in most cases with suicidal ideation and suicidal risk (Frank, 1946). A systematic review by Costanza et al. (2022b) of 18 studies that investigated the concept of demoralization has highlighted how economic insecurity (the economic crisis of 2008) and unsafe living conditions (the COVID-19 pandemic) impact people’s mental health, associated with an increased risk of suicide. Taking into consideration what was stated above, we may say that both victims of war (refugees) and inhabitants of countries that receive refugees and are exposed to information related to the armed conflict between Russia and Ukraine are currently living in unsafe conditions, which means that the two groups represent risk categories in terms of mental health, associated with the concept of demoralization. Given this situation, we wish to investigate how traumatic experiences influence those exposed to them.

In terms of psychological aspects of trauma exposure, Vinck et al. (2007) highlight the prevalence of depressive and PTSD symptoms among the war-exposed Ugandan population; these points are supported by Ahorsu et al. (2020), who mention how traumatic events that affect an entire population (for example pandemics, wars, economic crises) can cause worry, fear, and anxiety, and by Hajek et al. (2022), who show how exposure to conflicts such as war can lead to a much lower level of mental health. Kirby (2022) has recently highlighted how terrifying these events are, above all for the citizens of Ukraine but also for all the countries of Europe, as they register increased levels of anxiety as a consequence of the ongoing climate of uncertainty and aggression to which they have been exposed.

Furthermore, exposure to traumatic events instills a certain level of fear, which can have negative consequences for mental health in both adults and children. A situation in which fear is one of the predominant emotions can have various negative consequences, with the attention of specialists being concentrated especially on children, a particularly vulnerable category. It has been observed that both children (Joshi, and O’donnell, 2003; Shaw, 2003) and their parents (Thabet et al., 2008), when exposed to war or other political conflicts, develop PTSD-type symptoms, depression, anxiety, and other somatic symptoms. At the same time, Rometsch-Ogioun El Sount et al. (2018) explains how fear of war negatively impacts people’s mental health by causing them to worry about their loved ones (relatives, children), since they feel powerless in the face of this type of calamity.

In addition to these psychological consequences, fear can also lead to the adoption of various behaviors that exacerbate hysteria among the population, such as the spreading of false information about the various crises that humanity has endured. Thus, Elías and Catalan-Matamoros (2020) show how in Spain, during the coronavirus pandemic, the volume of information disseminated in the mass media increased, with a focus on mystery and the esoteric, an emphasis that contrasted sharply with the line being taken by the official sources. Not surprisingly, the contradiction itself led to even more uncertainty and confusion among people. This effect is also supported by the studies of Montesi (2021), Beauvais (2022), and Pomerance et al. (2022) all of whom have shown how concern about the pandemic generated uncertainty, which was then amplified by exposure to fake news.

### Health providers and exposure to war

In addition to children and their parents, a further category of people who are most often exposed to traumatic experiences and can develop some symptoms associated with the concept of demoralization is represented by those who work directly with people (doctors, psychologists, nurses). Figley (2002) shows that such work can result in consequences such as nightmares, insomnia, hopelessness, and other forms of secondary traumatic stress (indirect exposure to trauma through a traumatic event; Zimering et al., 2003). Cardozo et al. (2005) describe how Kosovan and Albanian aid workers implementing health programs in Kosovo and working with victims exposed to traumatic events reported symptoms of PTSD and depression, with support services being an important factor in ameliorating these. Similar symptoms have been reported for aid workers in Palestine (Veronese et al., 2017) and Uganda (Ager et al., 2012). Additionally, during the European refugee crisis, medically qualified people went to Greece to provide first aid which eventually resulted in their developing post-traumatic stress symptoms following exposure to these traumatic events (Sifaki-Pistolla et al., 2017). Given these results, which highlight a decline in mental health among humanitarian workers, it is desirable to find strategies to help such workers improve their psychological wellbeing. Veronese et al. (2013) show how a high level of sense of coherence can improve the mental health of staff working with victims of traumatic events. Maguen et al. (2008) also show how resilience played a protective role in the context of negative experiences and in promoting healthy coping strategies, psychological wellbeing, and good mental health among military medical personnel in Iraq.

Although the studies so far mentioned emphasize the development of strategies at the intrapersonal level among mental health workers, it is equally crucial to intervene in the case of the refugee population, since this directly affects the mental health of the residents of countries receiving war news or hosting refugees. In this context, Costanza et al. (2022b) have summarized the results of studies that tested the effectiveness of interventions among the refugee population designed to improve their mental health: some refugees to whom an intervention based on self-help was applied reported a lower level of depression and a higher level of quality of life 6 months after its implementation. As well as this, CBT is effective in reducing PTSD and anxiety symptoms, EMDR is effective in reducing depressive symptoms, while in the case of narrative therapy there is an absence of consensus (in some studies it was shown to be effective in reducing symptoms, while in other studies no effect was recorded). Thus, in the interventions we have mentioned, it can be seen that the focus is not on the quality of the information or on its transmission between people but on other aspects (e.g., changing thoughts and beliefs). This highlights the need to investigate how the content of information circulating on social media affects individuals when they are faced with a disaster. Doing this will enable us to create interventions (also based on the power of the word) that work for both groups involved (refugees, and people hosting/caring for refugees) on an interpersonal and intrapersonal level.

### Fake news, time spent online, and traumatic events

The spread of false information on social media has become a major problem in recent years. The worrying aspect of false information is that it spreads very quickly, potentially negatively affecting the political, economic, and social spheres (Vosoughi et al., 2018). To better understand the term “false information,” which has been in such general use in recent years, Fallis and Mathiesen (2019) undertook research that led them to the conclusion that false information represents counterfeit, fabricated information that is presented as being from reliable sources. The spread of fake news in times of crisis can have disastrous effects, as supported by the study of Zarocostas (2020), which shows how, during the COVID-19 pandemic, misinformation had a catastrophic effect not only on the health of individuals but also on their behaviors (people bought extraordinary amounts of toilet paper, disinfectant, and food; Naeem, 2021). Mukhtar (2020) points out that the conspiracy theories (especially regarding vaccination; Domgaard and Park, 2021) and misinformation that took social media by storm during the pandemic only alarmed the population, leading to a loss of calm and creating a state of hysteria. The studies we have cited show the link between exposure to traumatic events and post-traumatic stress syndrome, the latter variable being associated by Marco et al. (2020) with other stressors, such as the spread of false information about COVID-19 on social networks.

Thus, to combat the spread of fake news, we must first investigate what motivates people to share information without checking whether the source from which it comes is a reliable one. One of the reasons that could underlie these decisions relates to the extraordinary amount of information that appears on social media when humanity faces a crisis (Zhang et al., 2016, 2022; Bawden and Robinson, 2020; Tang et al., 2021; Tandoc and Kim, 2022), whether this is a health crisis or a political conflict. Loading social media with information can be a stressor among people (Bermes, 2021) who want to eliminate the uncertainty caused by the negative experience they are facing (Shu et al., 2020). Another aspect of information load is the tension that appears along with the news distributed on social media about the harmful event (Sulaiman et al., 2020; Al-Zaman, 2021; Molina et al., 2021; Zeng et al., 2021). Ayyagari et al. (2011) also make these points in their work on technostress. In the present study, “information stress” is used to refer to the fact that, when browsing social networks, people feel that their lives are becoming overwhelmed by information about the conflict between Russia and Ukraine. And when people feel overwhelmed by a huge amount of information, their desire to understand it or to search for accurate information is greatly reduced, which results in a lack of effort and motivation to check the sources and their accuracy (Catedrilla et al., 2020; Xu et al., 2022).

Another factor that could be related to the spread of fake news, which has such an influence, especially in times of crisis, is time spent online (Nelson and Taneja, 2018; Di Domenico et al., 2021; Pennycook and Rand, 2021; Obadă and Dabija, 2022). Taking into consideration the conclusions already stated, it can be assumed that, when people spend considerable amounts of time on social networks, exposed to the informational load and tension that inevitably accompany a global crisis, it is much more likely that they will spread false information (Weinreich et al., 2008; Fletcher et al., 2018; Apuke and Omar, 2021) rather than filtering it to see which is accurate.

Given the previously mentioned need for a more detailed investigation of the effectiveness of narrative therapy (there is no consensus in the specialist literature regarding its efficacy) in improving mental health among victims of war, it is important to observe how information related to the current conflict that is circulated in social media is taken up and distributed further by individuals. It is well known that false information shared on social media without being questioned is a cause of hysteria. By learning how this mechanism operates, we can use social media to combat the spread of fake news and the escalation of tension and to deliver online narrative-based interventions, in which experiences are rewritten with compassion, that contain expressions which can have a positive impact.

Even though Romania is not directly involved in the war between Ukraine and Russia, its people have from the beginning dedicated themselves to helping refugees, especially through voluntary action. According to official border police figures, by the beginning of December 2022 over 98,000 Ukrainians had crossed the border into Romania. However, even among Romanians, more and more information has been circulating via social media, causing a certain degree of hysteria, anxiety, and uncertainty about the future. This being the case, the present study has as its first objective an investigation of the impact that the uploading of information related to the armed conflict between Russia and Ukraine on social networks has on the distribution of fake news among Romanians. A secondary objective is to explore how certain psychological aspects such as resilience, general health, perceived stress, coping strategies, and fear of war change as a function of exposure to traumatic events or interaction with victims of war.

Drawing on a synthesis of the specialist literature, the following hypotheses were investigated:

H1. Information strain moderates the relationship between information overload and the likelihood of spreading fake news.

H2. Information strain moderates the relationship between time spent online and the likelihood of spreading fake news.

H3. Those who have interacted with victims of war will report higher levels of perceived stress and fear of war and lower levels of resilience and general health and will use different coping strategies to those who have not interacted with them.

Our research project therefore sets out to examine the impact of war proximity on Romanians both in the online environment, through exposure to information (time spent online, information overload, information strain and fake news regarding war), and in the offline environment, through interaction with war refugees (here we consider psychological issues related to the concept of demoralization).

## Materials and methods

### Participants

The sample included 633 participants aged between 18 and 73 (*M* = 24.58; *SD* = 9.47), of which 67.93% were female and 32.07% were male. Regarding their interaction with refugees from Ukraine, 200 of the 633 respondents mentioned some degree of interaction (21.55% made donations to help refugees, 4.91% volunteered in refugee centers, 2.38% are translators, 1.58% hosted refugees in their homes, 1.43% are educators, 0.79% social workers, 0.48% psychologists/counselors and 0.32% doctors), while the remaining 433 did not interact.

### Research instruments

#### General health

This variable was measured using the General Health Questionnaire (GHQ-12) (Goldberg and Williams, 1988), which consists of 12 items that measure on a Likert-type scale (from 0 to 3) the severity of a mental problem in the past 4 weeks. A total score, which could therefore range between 0 and 36, was obtained from the answers provided. The internal consistency of the scale in the present study was 0.90, 95% CI [0.891, 0.914].

#### Coping strategies

The Cognitive Emotion Regulation Questionnaire (CERQ) (Garnefski et al., 2001), in the version validated for the Romanian population (Perte and Miclea, 2011), was used as a measurement tool to see what type of strategy participants used when they were exposed to this specific type of disaster—war. The CERQ contains 36 items, reported on a Likert scale from 1 to 5, which are grouped into several subscales, each of which corresponds to an emotion regulation strategy (self-blame, acceptance, rumination, positive refocusing, refocus on planning, positive reappraisal, putting into perspective, catastrophizing, other-blame). A high score on a subscale indicates more frequent use of the corresponding coping strategy. The scale records good internal consistency (Cronbach’s *α* = 0.89, 95% CI [0.881, 0.905]).

#### Perceived stress

The Perceived Stress Scale (PSS; Cohen et al., 1994) was used. The 10 items of the scale measured respondents’ thoughts and emotions during the past month. Responses are rated on a Likert scale from 0 to 4, with higher scores indicating a higher level of perceived stress. In this study, the internal consistency of the scale was 0.86, 95% CI [0.844, 0.877].

#### Resilience

Respondents’ level of resilience was measured using the Brief Resilience Scale (BRS) (Smith et al., 2008). This scale contains six statements that participants had to evaluate, expressing their degree of agreement or disagreement on a scale from 1 to 5. The internal consistency of the scale was 0.84, 95% CI [0.827, 0.864].

#### Time spent online

For this variable, we used the Social Networking Time Use Scale (SONTUS) (Olufadi, 2016). This instrument presents 29 items in which subjects have to identify the number of times they have used social media in the past week in various contexts, using an 11-point Likert scale. The scale showed good internal consistency (Cronbach’s *α* = 0.92, 95% CI [0.917, 0.933]) in this study.

#### Fear of war

To evaluate this construct, the Fear of War Scale (FOWARS) (Kalcza-Janosi et al., 2022) was used. This scale contains 13 items, measured on a Likert scale from 1 to 5, through which respondents have to evaluate how characteristic of them the statements given in the questionnaire are. The total score is obtained by averaging the items, with a high score indicating a greater fear of war. The internal consistency reported in this study is 0.92, 95% CI [0.912, 0.930].

#### Informational overload and informational strain

To measure these variables, the information given in Luqman et al. (2017) study was used. We adapted four items for each of the two variables so that they fitted the present context, the conflict between Russia and Ukraine. Items were measured on a 7-point Likert scale, where 1 = strongly disagree and 7 = strongly agree. The internal consistencies for each of the two scales were 0.82 95% CI [0.806, 0.849], for information overload and 0.84, 95% CI [0.826, 0.865], for information strain.

#### The probability of spreading fake news

Two items were adapted in accordance with the information provided by Talwar et al. (2019), reported on a 7-point Likert scale. The internal consistency was. 92, 95% CI [0.917, 0.940].

### Procedure

Ethical approval for this study was obtained from the relevant departmental ethics committee (approval code 2298) and all research was conducted in accordance with the principles of the Declaration of Helsinki. To collect participant responses, a questionnaire was designed using Google Forms. Participants were recruited via advertisements on social media sites, supplemented using a snowball sampling method. All potential participants were provided with additional information about the requirements of the study and assured of the confidentiality and anonymity of the data and its use exclusively for scientific research purposes. In addition, they were informed of an estimated time to complete the questionnaire (20–25 min) and told that they could withdraw from the research at any point if they felt a high degree of discomfort or distress while working on the survey. Those who expressed an interest in being part of the research gave their consent via a digital form before completing, online, the questionnaire made up of the scales described above. The questionnaire was anonymous, and respondents participated as volunteers without being remunerated. Before actually completing the scales, participants were asked to provide their email addresses if they were willing to be contacted for participation in future studies. All data were collected between November 2022 and January 2023. Internet Protocol (IP) addresses were checked to ensure that no participant took the survey more than once.

## Results

Statistical analyses were performed using SPSS v20 and jamovi programs (The Jamovi Project, 2020). In jamovi, we used to evaluate multivariate models in general. To check the reliability of the questionnaires that evaluated the variables in the study, internal consistency α Cronbach coefficients were calculated. A first step before testing the actual hypotheses consisted in carrying out a descriptive analysis of the variables; the central tendency indicators for each of them can be observed in Table 1. Additionally, the assumptions for testing the hypotheses using parametric tests were verified. Since all the variables tested were symmetric, we used a moderation test to investigate whether information strain moderates firstly the relationship between information overload and the probability of sharing fake news and secondly the relationship between time spent online and fake news sharing. Also, we used the *t*-test for independent samples to compare the two groups (those who worked with refugees and those who did not) in terms of variables such as general health, coping strategies, resilience, perceived stress, and fear of war. Considering that the study is not an exploratory one (where it would be essential to adjust the *p*-values; Greenland and Hofman, 2019), an experimental or RCT type in which are tested several outcomes (Vickerstaff et al., 2019), in the present study, which is a cross-sectional one (correlational and comparative), we did not resort to adjusting the *p*-values when reporting the results.

**Table 1:** Descriptive statistics for study variables.

Variables	*M*	*SD*
General health (GHQ)	16.17	8.18
Time spent online (SONTUS)	12.97	4.06
Self-blame (CERQ)	12.61	3.52
Acceptance (CERQ)	14.52	3.39
Rumination (CERQ)	15.09	3.56
Positive refocusing (CERQ)	12.58	3.86
Refocus on planning (CERQ)	15.84	3.83
Positive reappraisal (CERQ)	15.40	3.56
Putting into perspective (CERQ)	14.30	3.72
Catastrophizing (CERQ)	10.49	3.82
Blaming others (CERQ)	9.67	3.72
Perceived stress (PSS)	19.39	7.26
Resilience (BRS)	3.20	0.839
Fear of war (FOWARS)	3.04	0.906
Information overload	16.34	6.06
Information strain	10.31	5.49
Fake news sharing	3.35	2.65

M, Mean; SD, Standard deviation.

### Moderation analyses

We tested the first moderation model using information overload as a predictor, the probability of spreading fake news as the dependent variable and information strain as the moderating variable. A main effect of extreme significance was found between information overload and the possibility of spreading fake news, *b* = −0.07, BCa CI [− 0.10, −0.04], *z* = −4.79, *p* < 0.001, along with a main effect of extreme significance between information strain and fake news spreading, *b* = 0.33, BCa CI [0.30, 0.36], *z* = 21.56, *p* < 0.001. An interaction of high significance was also observed between information overload and information strain, *b* = −0.01, BCa CI [− 0.01, −0.001], *z* = −2.65, *p* = 0.008. Participants who were exposed to high rather than medium or low levels of information strain were also exposed to a greater amount of information about the war, leading to a significantly higher likelihood of spreading fake news (*b* = −0.10, BCa CI [−0.15, −0.06], *z* = −4.72, *p* < 0.001). When participants were exposed to a low rather than a medium level of information strain, there was an absence of practical effect (*b* = −0.03, BCa CI [−0.06, −0.003], *z* = −1.78, *p* > 0.05, *p* = 0.07). Thus, from these results, we can conclude that the effect of social network information overload on the likelihood of spreading fake news is partially moderated by information strain. Thus, the hypothesis was sustained (see Table 2). However, it is recommended to interpret the results with caution. Regarding the interaction between informational overload and informational strain, a high significant one is recorded, but if we investigate the confidence interval, we notice that the lower threshold is very close to the null value, which could mean that the effect is of very low significance. Comparing the confidence interval of this interaction with the confidence interval observed following exposure to a low level of informational strain, we can see that its lower threshold is far from the null value even if the interaction has no practical significance, *p* = 0.07, very close to 0.05. This may mean that there could be an interaction of very low significance even if in this case was not recorded.

**Table 2:** Moderation estimates.

	Estimate	SE	*Z*	*p*	*s*
Information overload	−0.06774	0.01415	−4.79	< 0.001	−
Information strain	0.32750	0.01519	21.56	< 0.001	−
Information overload × information strain	−0.00671	0.00253	−2.65	0.008	6.97

s = *S*-value (*p*-value converted to s-value – in bits).

The second moderation model included as predictive variable time spent online, as dependent variable the probability of sharing fake news and as moderating variable information strain. A main effect of high significance was found between time spent online and the possibility of spreading fake news, *b* = 0.06, BCa CI [0.02, 0.10], *z* = 3.03, *p* = 0.002, along with a main effect of extreme significance between information strain and false news spreading, *b* = 0.27, BCa CI [0.24, 0.30], *z* = 17.65, *p* = < 0.001. An interaction of high significance was also observed between time spent online and information strain (*b* = 0.01, BCa CI [− 0.00, −0.02], *z* = 2.70, *p* = 0.007. Participants who were exposed to high rather than medium or low levels of information strain also reported a greater amount of time spent online, which led to a much higher probability of spreading fake news (*b* = 0.11, BCa CI [0.06, 0.17], *z* = 4.24, *p* < 0.001). When participants were exposed to a low rather than a medium level of information strain, there was an absence of practical effect (*b* = 0.01, BCa CI [−0.05, 0.07], *z* = 0.39, *p* > 0.05, *p* = 0.69). Thus, from these results, we can conclude that the effect of time spent online on the likelihood of spreading fake news is partially moderated by information strain, so the hypothesis is supported by the data analysis (see Table 3). However, it is recommended to interpret the results with caution. Regarding the interaction between time spent online and informational strain, a high significant one is recorded, but if we investigate the confidence interval, we notice that the lower threshold is very close to the null value, which could mean that the effect is of very low significance or without practical significance. Comparing the confidence interval of this interaction with the confidence interval observed following exposure to a low level of informational strain, we can see that its lower threshold is far from the null value even if the interaction has no practical significance, *p* = 0.69. This could lead to the possibility of finding an interaction of very low significance even if in this case was not recorded.

**Table 3:** Moderation estimates.

	Estimate	SE	*Z*	*p*	*s*
Time spent online	0.06250	0.02064	3.03	0.002	8.97
Information strain	0.26865	0.01522	17.65	<0.001	–
Time spent online × information strain	0.00929	0.00344	2.70	0.007	7.16

s = *S*-value (*p*-value converted to *s*-value – in bits).

### Comparisons between groups in terms of general health, perceived stress, resilience, coping strategies, and fear of war

We expected that those who had interacted with war victims would report higher levels of perceived stress and of fear of war, with lower levels of resilience and of general health, and would use different coping strategies to those who had not interacted with them. As we can see below (see Table 4), this hypothesis is only partially supported. Those who worked with refugees reported higher levels of fear of war, the size effect being small: *t*(633) = 2.343, *p* = 0.010, *d* = 0.20, than those who did not. Regarding coping strategies, we may observe that those who worked with refugees chose to focus on rumination as an emotional regulation strategy, the size effect being small: *t*(633) = 1.718, *p* = 0.04, *d* = 0.14, while those who did not work with refugees chose to focus on blaming others, the size effect being small in this case too: *t*(633) = −1.656, *p* = 0.04, *d* = 0.14.

**Table 4:** Comparisons between those who worked with refugees and those who did not.

Variables	The group who worked with refugees (*N* = 200)		The group who did not work with refugees (*N* = 433)		*t*	*p*	*s*	Size effect (Cohen’s *d*)
	*M*	*SD*	*M*	*SD*				
Self-blame	12.60	3.673	12.61	3.454	−0.049	0.480	1.06	–
Acceptance	14.40	3.601	14.57	3.303	−0.594	0.276	1.86	–
Rumination	15.45	3.455	14.92	3.613	1.718	0.043*	4.54	0.14
Positive refocusing	12.55	3.775	12.60	3.916	−0.161	0.436	1.2	–
Refocus on planning	15.89	3.119	15.81	3.289	0.260	0.397	1.33	–
Positive reappraisal	15.58	3.522	15.31	3.588	0.855	0.196	2.35	–
Putting into perspective	14.40	3.751	14.26	3.721	0.413	0.34	1.56	
Catastrophizing	10.36	3.829	10.55	3.824	−0.588	0.278	1.85	–
Blaming others	9.31	3.727	9.84	3.722	−1.656	0.049*	4.35	0.14
Perceived stress	19.62	7.141	19.28	7.325	0.544	0.293	1.77	–
Resilience	3.17	0.851	3.22	0.833	−0.705	0.240	2.06	–
General health	15.53	8.211	16.46	8.082	−1.339	0.090	3.47	–
Fear of war	3.17	0.904	2.99	0.903	2.343	0.010**	6.8	0.20

**p < 0.01; *p < 0.05; M, Mean; SD, Standard deviation; s = *S*-value (*p*-value converted to *s*-value – in bits).

As can be seen, the observed effect sizes are small, which suggests that the results obtained should be interpreted with caution. The low magnitude of the effect can be due to the lack of an association of the constructs in reality (the risk of committing a type 1 error that can occur when there is a large sample—statistically significant associations are obtained but of a low magnitude due to the large number of respondents even if in reality this association does not exist; it happens when the null hypothesis that is true in the general population is rejected) or of a lack of practical applicability of the results obtained. Another error that can occur is type 2 error, which it is not in the case of the present study (it happens when we have a small sample—we have a large effect, but the significance is low or non-existent; the null hypothesis that is false in the general population is not rejected). It is important then to investigate the accuracy of the effect size measure. For the first effect size, where we compared the fear of war level between the two groups: *d* = 0.20, we report the following confidence interval: 95% [−0.03, 0.37]. For the second effect size (those who worked with refugees chose to focus on rumination as emotional regulation strategy): *d* = 0.14, we report the following confidence interval: 95% [−0.02, 0.31]. As for the third effect size (those who did not work with refugees chose to focus on blaming others as emotional regulation strategy): *d* = 0.14, we report the following confidence interval: 95% [−0.31, 0.02]. As we can see, the width of the confidence intervals are very large, another reason to interpret the obtained results with caution (the fact that the intervals are very wide indicate that we do not have very much information about the effect; this implies there is a need for further studies on this topic to gain more knowledge).

## Discussions

The present study had two objectives. The primary objective was to investigate the reasons behind people’s tendency to share fake news on social media, especially when they are exposed to traumatic events. The secondary objective was to observe how specific psychological correlates change among frontline people who work and interact with war victims.

Following the statistical analysis, it was possible to observe the impact that information strain has on the probability of sharing fake news. The variable mentioned above (information strain) was a moderating factor of both (a) the relationship between social media information overload related to the armed conflict and the probability of spreading fake news, and (b) the relationship between time spent online and fake news sharing. Thus, our results regarding the link between information overload, information strain, and the probability of spreading fake news are consistent with those reported by Bermes (2021). We can explain these findings by adapting the transactional stress theory (Lazarus, 1993) to the present traumatic situation and arguing that when social media consumers are exposed to information strain (they feel that information about the war between Ukraine and Russia is everywhere, overwhelming and invading their lives), they will tend to resort to certain behaviors to avoid the negative emotions provoked by exposure to such information (Luqman et al., 2017). Thus, when social networks are loaded with information that only deals with the subject of war, it can be assumed that people will wish to avoid contact with this information as much as possible, so they will read the articles in question as superficially as they can and will not want to devote effort to deep processing of information or to checking whether the source is indeed accurate, which can lead in turn to a greater likelihood of spreading fake news (Laato et al., 2020). Regarding the link between time spent online, information strain, and the probability of spreading fake news, the results obtained in this study are consistent with those of Apuke and Omar (2021). They can be explained by the fact that when consumers spend the greater part of their time online rather than limiting their use of social media, they are very likely to have much more contact with the huge amount of information found online that is related to the armed conflict (Fletcher et al., 2018). This behavior can activate their desire to go through the information as quickly as possible (so that they are informed, which somewhat mitigates the uncertainty caused by the global crisis), and also their wish not to come into contact with reality through deep processing and to avoid the negative emotions that might arise from understanding the material read) and implicitly to further distribute news items of questionable quality (Huang et al., 2015).

Regarding the secondary objective of the study, the results showed that there were no significant differences between those who interacted with war victims and those who did not in terms of their degree of perceived stress, resilience, and general health. This probably happened because those who interacted with refugees were exposed to the traumatic event only indirectly, through the aid activities they carried out. That is why it is possible that the stress that comes with direct exposure to a traumatic event was not felt so strongly and, as an implicit consequence, that their general state of health was not affected to a significant extent. The explanatory hypotheses mentioned above are supported by the results of the systematic review of May and Wisco (2016), which show that indirect exposure to traumatic events can indeed have negative consequences for psychological wellbeing, but that the probability of developing a disorder such as PTSD merely from indirect exposure to trauma is much lower (Neria and Sullivan, 2011) than would be the case from direct exposure.

Turning to the emotional regulation strategies used by the two groups of participants, we noticed that those who interacted with war victims used rumination as an adaptive mechanism, a result consistent with those obtained by Szabo et al. (2017). Basharpoor et al. (2015) and Im and Follette (2016) state that rumination is frequently associated with exposure (direct or indirect) to traumatic events, with individuals tending to think repetitively about the consequences of these experiences, the emotions they provoked, and the causes of the events in question. This being the case, it can be assumed that those who interacted with refugees from Ukraine resorted to rumination since they had a greater opportunity to think about what they would have done in such a situation, how they would have felt, and how they would have behaved. At the same time, it can also be surmised that when these subjects were exposed to the war through their interaction with its victims, they gained a much better understanding of the situation these people were experiencing, hence the greater degree of fear of war recorded among those who helped refugees (Pine et al., 2005; Zhen et al., 2018). When we look at the strategies adopted by those who had not interacted with refugees, we can see that they resorted to blaming others for the traumatic conflict. Given that this was the category of participants least exposed to trauma (either directly or indirectly) we can understand the emotional regulation strategy they resorted to (those who started the war are to blame for what is happening). In the case of those directly exposed to trauma, the emotional regulation strategy often encountered is self-blame (McNally, 2003; Ceschi et al., 2014; Reich et al., 2021).

## Limitations

The present study investigates issues related to the war between Russia and Ukraine in both the online environment (social networks) and the offline environment (interaction with refugees), and was completed by a large sample of respondents. It also deals with the subject from the point of view of demoralization, a phenomenon often encountered in war victims, related to depression and high suicidal risk, which is to be introduced as a concept in the next version of the DSM. Regarding the limitations of the study, all the instruments used to measure the variables of interest were of the self-reported type, leading to a possibility that subjects might give answers that corresponded to their need to be socially acceptable. At the same time, there were no items designed to measure participants’ motivation for completing the questionnaire, so they may have run out of patience as they were filling it in and not paid great attention to the answers they were giving. Another limitation concerns the design of the study: it is cross-sectional, meaning that we cannot highlight causal links. A further limitation would be that only one of the instruments used had been validated for the Romanian population and that three of the constructs of interest were measured not by a specific scale but by items used by other authors in similar contexts, adapted to the needs of the present study.

## Practical implications

Although the effect sizes recorded in this study are low, suggesting a reduced practical applicability, but also the cautious interpretation of the results obtained due to the risk of a type I error, the present research brings with it the further investigation of the recorded phenomena. The subjects who participated in the study were exclusive of Romanian nationality, Romania not being a country directly involved in the war. In addition to the previously mentioned aspects, the fact that Romania did not directly participate in this armed conflict may represent another factor for the effects to be so low (not participating in the war, the psychological impact of the calamity may not have been so high). However, it is recommended to further investigate the psychological impact of disasters on people so that specialists know how to act in the future (interventions, psychological support). Although there is limited practical applicability of the study, it can represent a first step for carrying out other similar studies in the future to highlight the functioning mechanisms of people when natural disasters occur. This aspect can lead to the prevention of hysteria that occurs in such cases of uncertainty (let us take the example of the COVID-19 pandemic) by creating protocols for the effective management of crises.

## Conclusion

The exchange of information without prior verification is always harmful, but the seriousness of this problem is exacerbated during crises by the negative effects of false news. Although it is vital to help those who have been subjected to such disasters, it is also crucial to support those who care for them (counselors, doctors, volunteers). Given all that has been said above, it is imperative that these topics be studied further. As for the practical implications of the study, we assume that the moment we reach a clear understanding of the reasons why people share fake news, we will be able to combat the phenomenon using appropriate methods. Thus, Siricharoen and Siricharoen (2018) propose using infographics to reduce information overload on social networks, Roozenbeek and Van der Linden (2019) consider creating games to teach people how to detect fake news, and Okeke et al. (2018) regard it as necessary to develop an intervention to help people reduce the time they spend online. Regarding asylum seekers and the use of narratives, Costanza et al. (2022a) recommend an intervention based on meaning-centered therapy. It is also crucial to help those on the front line, the health providers, to be able to find resources and healthy coping strategies to stay healthy both physically and mentally. When the problems they face are discovered, specialists such as psychologists and psychotherapists will be able to create interventions to help these people reduce their levels of stress, burnout, and withdrawal. Naudé and Rothmann (2006) emphasize the need for psychological support and supervisors for health providers.

## Data availability statement

The raw data supporting the conclusions of this article will be made available by the authors, without undue reservation.

## Ethics statement

The studies involving human participants were reviewed and approved by the Ethics Committee of the West University of Timișoara. The relevant registration number is 2298/16.01.2023. The patients/participants provided their written informed consent to participate in this study.

## Author contributions

MV, G-ML, and AK: conceptualization and methodology of the study. OT, CG, and G-ML: data collection and data proofing. MV and G-ML: data analysis and manuscript writing. AK, OT, and CG: manuscript review. All authors contributed to the article and approved the submitted version.

## Conflict of interest

The authors declare that the research was conducted in the absence of any commercial or financial relationships that could be construed as a potential conflict of interest.

## Publisher’s note

All claims expressed in this article are solely those of the authors and do not necessarily represent those of their affiliated organizations, or those of the publisher, the editors and the reviewers. Any product that may be evaluated in this article, or claim that may be made by its manufacturer, is not guaranteed or endorsed by the publisher.
