# Supplementary material for: Fake news during the war in Ukraine: coping strategies and fear of war in the general population of Romania and in aid workers
Source: Front Psychol. 2023 May 12;14:1151794. doi: 10.3389/fpsyg.2023.1151794 (PMC10213335; doi:10.3389/fpsyg.2023.1151794)
Supplement: Supplementary file 1 [file Data_Sheet_1.PDF]

```
 GET
  FILE='D:\Psiho-An 6\Conferinta razboi\FearOfWar_database_1.sav'.
DATASET NAME DataSet1 WINDOW=FRONT.
RELIABILITY
  /VARIABLES=sontus1 sontus2 sontus3 sontus4 sontus5 sontus6 sontus7 sontus8 sontus9 sontus10 s
sontus29
  /SCALE('ALL VARIABLES') ALL
  /MODEL=ALPHA
  /STATISTICS=DESCRIPTIVE SCALE CORR COV
  /SUMMARY=TOTAL
  /ICC=MODEL(MIXED) TYPE(CONSISTENCY) CIN=95 TESTVAL=0.
```

Reliability

Notes

Output Created		16-APR-2023 17:59:28
Comments		
Input	Data	D:\Psiho-An 6\Conferinta razboi\FearOfWar_database_1.sav
	Active Dataset	DataSet1
	Filter	<none>
	Weight	<none>
	Split File	<none>
	N of Rows in Working Data File	633
	Matrix Input	D:\Psiho-An 6\Conferinta razboi\FearOfWar_database_1.sav
Missing Value Handling	Definition of Missing	User-defined missing values are treated as missing.
	Cases Used	Statistics are based on all cases with valid data for all variables in the procedure.

Notes

Syntax		RELIABILITY /VARIABLES=sontus1 sontus2 sontus3 sontus4 sontus5 sontus6 sontus7 sontus8 sontus9 sontus10 sontus11 sontus12 sontus13 sontus14 sontus15 sontus16 sontus17 sontus18 sontus19 sontus20 sontus21 sontus22 sontus23 sontus24 sontus25 sontus26 sontus27 sontus28 sontus29 /SCALE('ALL VARIABLES') ALL /MODEL=ALPHA /STATISTICS=DESCRIPTIVE SCALE CORR COV /SUMMARY=TOTAL /ICC=MODEL(MIXED) TYPE(CONSISTENCY) CIN=95 TESTVAL=0.
Resources	Processor Time	00:00:00.03
	Elapsed Time	00:00:00.05

```
[DataSet1] D:\Psiho-An 6\Conferinta razboi\FearOfWar_database_1.sav
```

Scale: ALL VARIABLES

Case Processing Summary

		N	%
Cases	Valid	633	100.0
	Excluded[a]	0	.0
	Total	633	100.0

a. Listwise deletion based on all variables in the procedure.

Reliability Statistics

Cronbach's Alpha	Cronbach's Alpha Based on Standardized Items	N of Items
.925	.925	29

Item Statistics

	Mean	Std. Deviation	N
sontus1	2.09	1.092	633
sontus2	3.42	.833	633
sontus3	2.94	1.094	633
sontus4	1.61	.940	633
sontus5	2.27	1.081	633
sontus6	2.89	1.044	633
sontus7	2.49	1.195	633
sontus8	2.86	1.121	633
sontus9	1.54	.847	633
sontus10	2.05	1.037	633
sontus11	3.11	1.010	633
sontus12	2.74	1.132	633
sontus13	1.74	.960	633
sontus14	2.19	1.140	633
sontus15	2.77	1.095	633
sontus16	2.83	1.074	633
sontus17	2.15	1.016	633
sontus18	2.69	1.127	633
sontus19	1.56	.938	633
sontus20	2.55	1.123	633
sontus21	1.80	.982	633
sontus22	2.26	1.067	633
sontus23	1.41	.796	633
sontus24	2.53	1.095	633
sontus25	2.62	1.123	633
sontus26	2.00	1.104	633
sontus27	2.24	1.140	633
sontus28	2.41	1.108	633
sontus29	2.24	1.136	633

Inter-Item Correlation Matrix

	sontus1	sontus2	sontus3	sontus4	sontus5	sontus6	sontus7	sontus8
sontus1	1.000	.198	.252	.387	.329	.245	.268	.187
sontus2	.198	1.000	.544	.012	.247	.398	.319	.464
sontus3	.252	.544	1.000	.125	.303	.359	.335	.728
sontus4	.387	.012	.125	1.000	.310	.209	.226	.112
sontus5	.329	.247	.303	.310	1.000	.363	.392	.356
sontus6	.245	.398	.359	.209	.363	1.000	.405	.423
sontus7	.268	.319	.335	.226	.392	.405	1.000	.392
sontus8	.187	.464	.728	.112	.356	.423	.392	1.000
sontus9	.353	-.019	.109	.517	.346	.160	.286	.117
sontus10	.490	.192	.236	.318	.499	.353	.348	.306
sontus11	.189	.440	.338	.088	.278	.396	.254	.381
sontus12	.249	.447	.462	.137	.304	.427	.338	.425
sontus13	.389	.018	.195	.374	.417	.217	.361	.220
sontus14	.219	.139	.209	.355	.217	.340	.231	.264
sontus15	.208	.448	.714	.113	.344	.394	.354	.764
sontus16	.180	.443	.669	.136	.349	.364	.375	.702
sontus17	.308	.170	.249	.356	.320	.354	.328	.267
sontus18	.126	.228	.116	.073	.231	.213	.138	.184
sontus19	.281	-.055	.090	.408	.202	.191	.200	.125
sontus20	.273	.259	.291	.263	.267	.368	.265	.339
sontus21	.263	.063	.094	.390	.324	.206	.264	.135
sontus22	.277	.250	.332	.293	.404	.298	.358	.351
sontus23	.280	-.113	.050	.461	.246	.102	.177	.039
sontus24	.210	.247	.251	.271	.292	.437	.308	.319
sontus25	.180	.199	.236	.195	.165	.299	.220	.269
sontus26	.218	.104	.215	.340	.267	.362	.311	.255
sontus27	.248	.175	.424	.333	.304	.311	.305	.464
sontus28	.279	.236	.275	.231	.470	.341	.312	.336
sontus29	.271	.142	.231	.268	.422	.279	.302	.284

Inter-Item Correlation Matrix

	sontus9	sontus10	sontus11	sontus12	sontus13	sontus14	sontus15
sontus1	.353	.490	.189	.249	.389	.219	.208
sontus2	-.019	.192	.440	.447	.018	.139	.448
sontus3	.109	.236	.338	.462	.195	.209	.714
sontus4	.517	.318	.088	.137	.374	.355	.113
sontus5	.346	.499	.278	.304	.417	.217	.344
sontus6	.160	.353	.396	.427	.217	.340	.394
sontus7	.286	.348	.254	.338	.361	.231	.354
sontus8	.117	.306	.381	.425	.220	.264	.764
sontus9	1.000	.446	.075	.161	.562	.284	.158
sontus10	.446	1.000	.246	.326	.491	.276	.323
sontus11	.075	.246	1.000	.334	.120	.178	.404
sontus12	.161	.326	.334	1.000	.229	.295	.462
sontus13	.562	.491	.120	.229	1.000	.346	.254
sontus14	.284	.276	.178	.295	.346	1.000	.286
sontus15	.158	.323	.404	.462	.254	.286	1.000
sontus16	.163	.284	.373	.479	.231	.290	.790
sontus17	.407	.435	.211	.320	.402	.344	.290
sontus18	.169	.197	.397	.236	.193	.170	.201
sontus19	.462	.364	.045	.107	.380	.303	.158
sontus20	.192	.302	.368	.371	.278	.342	.326
sontus21	.555	.388	.133	.197	.443	.242	.156
sontus22	.358	.373	.272	.399	.403	.352	.369
sontus23	.640	.343	-.009	.080	.487	.248	.132
sontus24	.259	.357	.322	.403	.312	.292	.330
sontus25	.141	.227	.305	.253	.192	.289	.260
sontus26	.370	.383	.164	.296	.394	.419	.288
sontus27	.287	.321	.289	.360	.308	.388	.468
sontus28	.283	.421	.268	.276	.406	.271	.340
sontus29	.305	.362	.222	.207	.410	.267	.289

Inter-Item Correlation Matrix

	sontus16	sontus17	sontus18	sontus19	sontus20	sontus21	sontus22
sontus1	.180	.308	.126	.281	.273	.263	.277
sontus2	.443	.170	.228	-.055	.259	.063	.250
sontus3	.669	.249	.116	.090	.291	.094	.332
sontus4	.136	.356	.073	.408	.263	.390	.293
sontus5	.349	.320	.231	.202	.267	.324	.404
sontus6	.364	.354	.213	.191	.368	.206	.298
sontus7	.375	.328	.138	.200	.265	.264	.358
sontus8	.702	.267	.184	.125	.339	.135	.351
sontus9	.163	.407	.169	.462	.192	.555	.358
sontus10	.284	.435	.197	.364	.302	.388	.373
sontus11	.373	.211	.397	.045	.368	.133	.272
sontus12	.479	.320	.236	.107	.371	.197	.399
sontus13	.231	.402	.193	.380	.278	.443	.403
sontus14	.290	.344	.170	.303	.342	.242	.352
sontus15	.790	.290	.201	.158	.326	.156	.369
sontus16	1.000	.310	.186	.125	.352	.191	.379
sontus17	.310	1.000	.240	.333	.335	.514	.408
sontus18	.186	.240	1.000	.099	.382	.293	.236
sontus19	.125	.333	.099	1.000	.220	.415	.275
sontus20	.352	.335	.382	.220	1.000	.285	.371
sontus21	.191	.514	.293	.415	.285	1.000	.372
sontus22	.379	.408	.236	.275	.371	.372	1.000
sontus23	.085	.353	.113	.558	.196	.531	.332
sontus24	.337	.398	.228	.267	.380	.295	.380
sontus25	.256	.259	.284	.208	.591	.233	.240
sontus26	.290	.347	.109	.400	.334	.298	.329
sontus27	.478	.368	.159	.310	.384	.267	.405
sontus28	.357	.334	.246	.213	.342	.264	.411
sontus29	.292	.303	.206	.262	.367	.293	.415

Inter-Item Correlation Matrix

	sontus23	sontus24	sontus25	sontus26	sontus27	sontus28	sontus29
sontus1	.280	.210	.180	.218	.248	.279	.271
sontus2	-.113	.247	.199	.104	.175	.236	.142
sontus3	.050	.251	.236	.215	.424	.275	.231
sontus4	.461	.271	.195	.340	.333	.231	.268
sontus5	.246	.292	.165	.267	.304	.470	.422
sontus6	.102	.437	.299	.362	.311	.341	.279
sontus7	.177	.308	.220	.311	.305	.312	.302
sontus8	.039	.319	.269	.255	.464	.336	.284
sontus9	.640	.259	.141	.370	.287	.283	.305
sontus10	.343	.357	.227	.383	.321	.421	.362
sontus11	-.009	.322	.305	.164	.289	.268	.222
sontus12	.080	.403	.253	.296	.360	.276	.207
sontus13	.487	.312	.192	.394	.308	.406	.410
sontus14	.248	.292	.289	.419	.388	.271	.267
sontus15	.132	.330	.260	.288	.468	.340	.289
sontus16	.085	.337	.256	.290	.478	.357	.292
sontus17	.353	.398	.259	.347	.368	.334	.303
sontus18	.113	.228	.284	.109	.159	.246	.206
sontus19	.558	.267	.208	.400	.310	.213	.262
sontus20	.196	.380	.591	.334	.384	.342	.367
sontus21	.531	.295	.233	.298	.267	.264	.293
sontus22	.332	.380	.240	.329	.405	.411	.415
sontus23	1.000	.235	.178	.357	.313	.259	.298
sontus24	.235	1.000	.319	.429	.351	.389	.318
sontus25	.178	.319	1.000	.312	.426	.306	.309
sontus26	.357	.429	.312	1.000	.503	.313	.388
sontus27	.313	.351	.426	.503	1.000	.352	.403
sontus28	.259	.389	.306	.313	.352	1.000	.673
sontus29	.298	.318	.309	.388	.403	.673	1.000

Inter-Item Covariance Matrix

	sontus1	sontus2	sontus3	sontus4	sontus5	sontus6	sontus7	sontus8
sontus1	1.193	.180	.301	.397	.389	.279	.350	.229
sontus2	.180	.694	.496	.009	.222	.346	.317	.433
sontus3	.301	.496	1.196	.128	.359	.410	.438	.892
sontus4	.397	.009	.128	.884	.315	.205	.254	.118
sontus5	.389	.222	.359	.315	1.168	.410	.507	.432
sontus6	.279	.346	.410	.205	.410	1.090	.505	.495
sontus7	.350	.317	.438	.254	.507	.505	1.428	.525
sontus8	.229	.433	.892	.118	.432	.495	.525	1.257
sontus9	.326	-.014	.100	.412	.316	.142	.289	.111
sontus10	.555	.166	.267	.310	.560	.383	.431	.356
sontus11	.208	.370	.373	.083	.304	.418	.307	.432
sontus12	.308	.421	.572	.146	.373	.504	.457	.540
sontus13	.408	.014	.205	.338	.433	.217	.414	.237
sontus14	.272	.132	.261	.381	.267	.404	.315	.337
sontus15	.249	.408	.856	.117	.407	.451	.463	.938
sontus16	.212	.396	.786	.138	.405	.408	.481	.846
sontus17	.341	.144	.277	.340	.352	.376	.398	.304
sontus18	.155	.214	.143	.077	.281	.251	.185	.233
sontus19	.288	-.043	.092	.360	.205	.187	.224	.131
sontus20	.335	.242	.357	.278	.324	.432	.355	.427
sontus21	.282	.052	.101	.360	.344	.212	.309	.149
sontus22	.323	.222	.387	.294	.465	.332	.457	.420
sontus23	.243	-.075	.044	.345	.212	.085	.168	.035
sontus24	.251	.225	.301	.279	.346	.500	.403	.391
sontus25	.221	.186	.290	.206	.201	.351	.296	.339
sontus26	.263	.095	.259	.353	.319	.417	.411	.316
sontus27	.309	.166	.529	.357	.375	.370	.415	.593
sontus28	.338	.218	.334	.240	.563	.395	.413	.417
sontus29	.336	.134	.287	.287	.518	.331	.410	.361

Inter-Item Covariance Matrix

	sontus9	sontus10	sontus11	sontus12	sontus13	sontus14	sontus15
sontus1	.326	.555	.208	.308	.408	.272	.249
sontus2	-.014	.166	.370	.421	.014	.132	.408
sontus3	.100	.267	.373	.572	.205	.261	.856
sontus4	.412	.310	.083	.146	.338	.381	.117
sontus5	.316	.560	.304	.373	.433	.267	.407
sontus6	.142	.383	.418	.504	.217	.404	.451
sontus7	.289	.431	.307	.457	.414	.315	.463
sontus8	.111	.356	.432	.540	.237	.337	.938
sontus9	.717	.392	.064	.155	.457	.274	.146
sontus10	.392	1.076	.258	.382	.489	.327	.367
sontus11	.064	.258	1.021	.382	.116	.205	.447
sontus12	.155	.382	.382	1.283	.249	.381	.573
sontus13	.457	.489	.116	.249	.922	.379	.267
sontus14	.274	.327	.205	.381	.379	1.299	.357
sontus15	.146	.367	.447	.573	.267	.357	1.199
sontus16	.148	.316	.405	.583	.238	.355	.929
sontus17	.350	.459	.216	.368	.393	.398	.323
sontus18	.161	.231	.451	.301	.209	.218	.247
sontus19	.367	.354	.043	.114	.342	.324	.162
sontus20	.183	.352	.418	.472	.300	.437	.401
sontus21	.462	.395	.132	.219	.417	.271	.168
sontus22	.324	.413	.293	.482	.413	.428	.431
sontus23	.432	.284	-.008	.072	.372	.225	.115
sontus24	.240	.405	.357	.500	.328	.364	.396
sontus25	.134	.264	.347	.322	.207	.370	.319
sontus26	.346	.439	.182	.371	.418	.528	.349
sontus27	.277	.380	.333	.465	.337	.504	.584
sontus28	.266	.484	.300	.347	.431	.342	.413
sontus29	.294	.427	.255	.266	.448	.345	.359

Inter-Item Covariance Matrix

	sontus16	sontus17	sontus18	sontus19	sontus20	sontus21	sontus22
sontus1	.212	.341	.155	.288	.335	.282	.323
sontus2	.396	.144	.214	-.043	.242	.052	.222
sontus3	.786	.277	.143	.092	.357	.101	.387
sontus4	.138	.340	.077	.360	.278	.360	.294
sontus5	.405	.352	.281	.205	.324	.344	.465
sontus6	.408	.376	.251	.187	.432	.212	.332
sontus7	.481	.398	.185	.224	.355	.309	.457
sontus8	.846	.304	.233	.131	.427	.149	.420
sontus9	.148	.350	.161	.367	.183	.462	.324
sontus10	.316	.459	.231	.354	.352	.395	.413
sontus11	.405	.216	.451	.043	.418	.132	.293
sontus12	.583	.368	.301	.114	.472	.219	.482
sontus13	.238	.393	.209	.342	.300	.417	.413
sontus14	.355	.398	.218	.324	.437	.271	.428
sontus15	.929	.323	.247	.162	.401	.168	.431
sontus16	1.154	.338	.225	.126	.424	.201	.434
sontus17	.338	1.033	.275	.317	.382	.513	.443
sontus18	.225	.275	1.270	.104	.484	.324	.284
sontus19	.126	.317	.104	.879	.232	.382	.275
sontus20	.424	.382	.484	.232	1.261	.314	.444
sontus21	.201	.513	.324	.382	.314	.964	.390
sontus22	.434	.443	.284	.275	.444	.390	1.138
sontus23	.073	.286	.101	.417	.175	.415	.282
sontus24	.396	.443	.281	.274	.467	.317	.444
sontus25	.308	.296	.360	.219	.745	.257	.287
sontus26	.344	.390	.136	.414	.414	.323	.387
sontus27	.586	.426	.204	.331	.491	.299	.493
sontus28	.425	.376	.308	.221	.425	.287	.486
sontus29	.356	.350	.264	.279	.468	.327	.503

Inter-Item Covariance Matrix

	sontus23	sontus24	sontus25	sontus26	sontus27	sontus28	sontus29
sontus1	.243	.251	.221	.263	.309	.338	.336
sontus2	-.075	.225	.186	.095	.166	.218	.134
sontus3	.044	.301	.290	.259	.529	.334	.287
sontus4	.345	.279	.206	.353	.357	.240	.287
sontus5	.212	.346	.201	.319	.375	.563	.518
sontus6	.085	.500	.351	.417	.370	.395	.331
sontus7	.168	.403	.296	.411	.415	.413	.410
sontus8	.035	.391	.339	.316	.593	.417	.361
sontus9	.432	.240	.134	.346	.277	.266	.294
sontus10	.284	.405	.264	.439	.380	.484	.427
sontus11	-.008	.357	.347	.182	.333	.300	.255
sontus12	.072	.500	.322	.371	.465	.347	.266
sontus13	.372	.328	.207	.418	.337	.431	.448
sontus14	.225	.364	.370	.528	.504	.342	.345
sontus15	.115	.396	.319	.349	.584	.413	.359
sontus16	.073	.396	.308	.344	.586	.425	.356
sontus17	.286	.443	.296	.390	.426	.376	.350
sontus18	.101	.281	.360	.136	.204	.308	.264
sontus19	.417	.274	.219	.414	.331	.221	.279
sontus20	.175	.467	.745	.414	.491	.425	.468
sontus21	.415	.317	.257	.323	.299	.287	.327
sontus22	.282	.444	.287	.387	.493	.486	.503
sontus23	.634	.205	.160	.314	.284	.229	.269
sontus24	.205	1.199	.392	.518	.438	.471	.396
sontus25	.160	.392	1.261	.387	.545	.381	.394
sontus26	.314	.518	.387	1.220	.634	.383	.487
sontus27	.284	.438	.545	.634	1.301	.445	.523
sontus28	.229	.471	.381	.383	.445	1.227	.847
sontus29	.269	.396	.394	.487	.523	.847	1.290

Item-Total Statistics

	Scale Mean if Item Deleted	Scale Variance if Item Deleted	Corrected Item-Total Correlation	Squared Multiple Correlation	Cronbach's Alpha if Item Deleted
sontus1	65.91	284.359	.453	.359	.924
sontus2	64.58	290.200	.400	.477	.924
sontus3	65.06	281.362	.537	.664	.923
sontus4	66.39	287.117	.447	.420	.924
sontus5	65.73	280.682	.563	.432	.922
sontus6	65.11	281.535	.560	.433	.922
sontus7	65.51	279.833	.525	.342	.923
sontus8	65.14	278.921	.589	.691	.922
sontus9	66.46	287.226	.499	.579	.923
sontus10	65.94	280.286	.601	.498	.922
sontus11	64.89	285.848	.450	.386	.924
sontus12	65.25	280.320	.545	.428	.922
sontus13	66.26	283.174	.562	.503	.922
sontus14	65.81	282.152	.491	.335	.923
sontus15	65.23	278.575	.615	.743	.921
sontus16	65.17	279.327	.606	.689	.922
sontus17	65.85	281.470	.579	.425	.922
sontus18	65.31	287.570	.351	.297	.925
sontus19	66.44	287.889	.424	.411	.924
sontus20	65.45	279.432	.574	.498	.922
sontus21	66.20	284.840	.496	.492	.923
sontus22	65.73	279.442	.608	.413	.922
sontus23	66.59	290.099	.425	.572	.924
sontus24	65.47	280.395	.563	.392	.922
sontus25	65.38	283.422	.465	.426	.924
sontus26	66.00	280.636	.551	.452	.922
sontus27	65.76	277.558	.616	.507	.921
sontus28	65.58	279.455	.582	.548	.922
sontus29	65.76	279.924	.553	.531	.922

Scale Statistics

Mean	Variance	Std. Deviation	N of Items
68.00	302.252	17.385	29

Intraclass Correlation Coefficient

	Intraclass Correlation[b]	95% Confidence Interval		F Test with True Value 0		
		Lower Bound	Upper Bound	Value	df1	df2
Single Measures	.299[a]	.275	.326	13.366	632	17696
Average Measures	.925[c]	.917	.933	13.366	632	17696

Intraclass Correlation Coefficient

	F Test ...
	Sig
Single Measures	.000
Average Measures	.000

Two-way mixed effects model where people effects are random and measures effects are fixed.

a. The estimator is the same, whether the interaction effect is present or not.

b. Type C intraclass correlation coefficients using a consistency definition-the between-measure variance is excluded from the denominator variance.

c. This estimate is computed assuming the interaction effect is absent, because it is not estimable otherwise.

```
RELIABILITY
  /VARIABLES=ghq1p ghq2p ghq3p ghq4p ghq5p ghq6p ghq1n ghq2n ghq3n ghq4n ghq5n ghq6n
  /SCALE('ALL VARIABLES') ALL
  /MODEL=ALPHA
  /STATISTICS=DESCRIPTIVE SCALE CORR COV
  /SUMMARY=TOTAL
  /ICC=MODEL(MIXED) TYPE(CONSISTENCY) CIN=95 TESTVAL=0.
```

Reliability

Notes

Output Created		16-APR-2023 18:00:32
Comments		
Input	Data	D:\Psiho-An 6\Conferinta razboi\FearOfWar_database_1.sav
	Active Dataset	DataSet1
	Filter	<none>
	Weight	<none>
	Split File	<none>
	N of Rows in Working Data File	633
	Matrix Input	
Missing Value Handling	Definition of Missing	User-defined missing values are treated as missing.
	Cases Used	Statistics are based on all cases with valid data for all variables in the procedure.
Syntax		RELIABILITY /VARIABLES=ghq1p ghq2p ghq3p ghq4p ghq5p ghq6p ghq1n ghq2n ghq3n ghq4n ghq5n ghq6n /SCALE('ALL VARIABLES') ALL /MODEL=ALPHA /STATISTICS=DESCRIPTIVE SCALE CORR COV /SUMMARY=TOTAL /ICC=MODEL(MIXED) TYPE(CONSISTENCY) CIN=95 TESTVAL=0.
Resources	Processor Time	00:00:00.00
	Elapsed Time	00:00:00.00

```
[DataSet1] D:\Psiho-An 6\Conferinta razboi\FearOfWar_database_1.sav
```

Scale: ALL VARIABLES

Case Processing Summary

		N	%
Cases	Valid	633	100.0
	Excluded[a]	0	.0
	Total	633	100.0

a. Listwise deletion based on all variables in the procedure.

Reliability Statistics

Cronbach's Alpha	Cronbach's Alpha Based on Standardized Items	N of Items
.903	.903	12

Item Statistics

	Mean	Std. Deviation	N
ghq1p	1.48	.886	633
ghq2p	1.38	.931	633
ghq3p	1.32	.954	633
ghq4p	1.45	.952	633
ghq5p	1.36	.924	633
ghq6p	1.34	.929	633
ghq1n	1.47	1.045	633
ghq2n	1.59	.984	633
ghq3n	1.36	.984	633
ghq4n	1.36	1.014	633
ghq5n	1.12	1.023	633
ghq6n	.93	1.049	633

Inter-Item Correlation Matrix

	ghq1p	ghq2p	ghq3p	ghq4p	ghq5p	ghq6p	ghq1n	ghq2n
ghq1p	1.000	.564	.562	.521	.515	.502	.230	.342
ghq2p	.564	1.000	.579	.514	.494	.514	.134	.193
ghq3p	.562	.579	1.000	.624	.622	.599	.182	.270
ghq4p	.521	.514	.624	1.000	.633	.657	.272	.326
ghq5p	.515	.494	.622	.633	1.000	.652	.294	.313
ghq6p	.502	.514	.599	.657	.652	1.000	.296	.324
ghq1n	.230	.134	.182	.272	.294	.296	1.000	.546
ghq2n	.342	.193	.270	.326	.313	.324	.546	1.000
ghq3n	.385	.292	.350	.357	.428	.395	.502	.631
ghq4n	.415	.301	.358	.374	.386	.420	.460	.558
ghq5n	.374	.292	.340	.358	.385	.420	.462	.530
ghq6n	.348	.344	.363	.363	.367	.417	.415	.411

Inter-Item Correlation Matrix

	ghq3n	ghq4n	ghq5n	ghq6n
ghq1p	.385	.415	.374	.348
ghq2p	.292	.301	.292	.344
ghq3p	.350	.358	.340	.363
ghq4p	.357	.374	.358	.363
ghq5p	.428	.386	.385	.367
ghq6p	.395	.420	.420	.417
ghq1n	.502	.460	.462	.415
ghq2n	.631	.558	.530	.411
ghq3n	1.000	.637	.629	.561
ghq4n	.637	1.000	.683	.613
ghq5n	.629	.683	1.000	.722
ghq6n	.561	.613	.722	1.000

Inter-Item Covariance Matrix

	ghq1p	ghq2p	ghq3p	ghq4p	ghq5p	ghq6p	ghq1n	ghq2n
ghq1p	.785	.465	.474	.439	.421	.413	.213	.298
ghq2p	.465	.866	.514	.455	.425	.445	.131	.177
ghq3p	.474	.514	.909	.567	.548	.531	.181	.253
ghq4p	.439	.455	.567	.906	.557	.582	.270	.305
ghq5p	.421	.425	.548	.557	.854	.560	.283	.285
ghq6p	.413	.445	.531	.582	.560	.864	.288	.296
ghq1n	.213	.131	.181	.270	.283	.288	1.091	.561
ghq2n	.298	.177	.253	.305	.285	.296	.561	.968
ghq3n	.336	.267	.329	.334	.389	.361	.516	.610
ghq4n	.372	.284	.346	.361	.362	.395	.488	.557
ghq5n	.339	.278	.331	.348	.363	.399	.493	.533
ghq6n	.324	.336	.363	.363	.356	.407	.454	.425

Inter-Item Covariance Matrix

	ghq3n	ghq4n	ghq5n	ghq6n
ghq1p	.336	.372	.339	.324
ghq2p	.267	.284	.278	.336
ghq3p	.329	.346	.331	.363
ghq4p	.334	.361	.348	.363
ghq5p	.389	.362	.363	.356
ghq6p	.361	.395	.399	.407
ghq1n	.516	.488	.493	.454
ghq2n	.610	.557	.533	.425
ghq3n	.969	.636	.634	.580
ghq4n	.636	1.028	.708	.652
ghq5n	.634	.708	1.046	.775
ghq6n	.580	.652	.775	1.101

Item-Total Statistics

	Scale Mean if Item Deleted	Scale Variance if Item Deleted	Corrected Item-Total Correlation	Squared Multiple Correlation	Cronbach's Alpha if Item Deleted
ghq1p	14.69	57.095	.612	.468	.896
ghq2p	14.79	57.650	.535	.458	.899
ghq3p	14.84	56.285	.620	.560	.895
ghq4p	14.71	55.999	.643	.562	.894
ghq5p	14.81	56.115	.657	.564	.894
ghq6p	14.83	55.853	.673	.571	.893
ghq1n	14.70	57.222	.491	.378	.902
ghq2n	14.58	56.504	.581	.508	.897
ghq3n	14.80	55.117	.683	.582	.892
ghq4n	14.81	54.719	.688	.582	.892
ghq5n	15.04	54.619	.688	.647	.892
ghq6n	15.24	54.899	.648	.577	.894

Scale Statistics

Mean	Variance	Std. Deviation	N of Items
16.17	66.069	8.128	12

Intraclass Correlation Coefficient

	Intraclass Correlation[b]	95% Confidence Interval		F Test with True Value 0		
		Lower Bound	Upper Bound	Value	df1	df2
Single Measures	.437[a]	.406	.469	10.299	632	6952
Average Measures	.903[c]	.891	.914	10.299	632	6952

Intraclass Correlation Coefficient

	F Test ...
	Sig
Single Measures	.000
Average Measures	.000

Two-way mixed effects model where people effects are random and measures effects are fixed.

a. The estimator is the same, whether the interaction effect is present or not.

b. Type C intraclass correlation coefficients using a consistency definition-the between-measure variance is excluded from the denominator variance.

c. This estimate is computed assuming the interaction effect is absent, because it is not estimable otherwise.

```
RELIABILITY
 /VARIABLES=cerq1 cerq2 cerq3 cerq4 cerq5 cerq6 cerq7 cerq8 cerq9 cerq10 cerq11 cerq12 cerq13
 /SCALE('ALL VARIABLES') ALL
```

```
/MODEL=ALPHA
/STATISTICS=DESCRIPTIVE SCALE CORR COV
/SUMMARY=TOTAL
/ICC=MODEL(MIXED) TYPE(CONSISTENCY) CIN=95 TESTVAL=0.
```

Reliability

Notes

Output Created		16-APR-2023 18:01:45
Comments		
Input	Data	D:\Psiho-An 6\Conferinta razboi\FearOfWar_database_1.sav
	Active Dataset	DataSet1
	Filter	<none>
	Weight	<none>
	Split File	<none>
	N of Rows in Working Data File	633
	Matrix Input	
Missing Value Handling	Definition of Missing	User-defined missing values are treated as missing.
	Cases Used	Statistics are based on all cases with valid data for all variables in the procedure.
Syntax		RELIABILITY /VARIABLES=cerq1 cerq2 cerq3 cerq4 cerq5 cerq6 cerq7 cerq8 cerq9 cerq10 cerq11 cerq12 cerq13 cerq14 cerq15 cerq16 cerq17 cerq18 cerq19 cerq20 cerq21 cerq22 cerq23 cerq24 cerq25 cerq26 cerq27 cerq28 cerq29 cerq30 cerq31 cerq32 cerq33 cerq34 cerq35 cerq36 /SCALE('ALL VARIABLES') ALL /MODEL=ALPHA /STATISTICS=DESCRIPTIVE SCALE CORR COV /SUMMARY=TOTAL /ICC=MODEL(MIXED) TYPE(CONSISTENCY) CIN=95 TESTVAL=0.

Notes

Resources	Processor Time	00:00:00.02
	Elapsed Time	00:00:00.02

[DataSet1] D:\Psiho-An 6\Conferinta razboi\FearOfWar_database_1.sav

Scale: ALL VARIABLES

Case Processing Summary

		N	%
Cases	Valid	631	99.7
	Excluded[a]	2	.3
	Total	633	100.0

a. Listwise deletion based on all variables in the procedure.

Reliability Statistics

Cronbach's Alpha	Cronbach's Alpha Based on Standardized Items	N of Items
.893	.895	36

Item Statistics

	Mean	Std. Deviation	N
cerq1	2.71	1.234	631
cerq2	3.71	1.111	631
cerq3	3.76	1.104	631
cerq4	3.23	1.193	631
cerq5	4.09	1.013	631
cerq6	4.09	1.050	631
cerq7	3.67	1.155	631
cerq8	2.60	1.273	631
cerq9	2.25	1.122	631
cerq10	3.17	1.158	631
cerq11	3.85	1.065	631
cerq12	3.76	1.124	631
cerq13	3.12	1.244	631
cerq14	4.04	1.003	631
cerq15	3.98	1.118	631
cerq16	3.61	1.256	631
cerq17	2.81	1.188	631
cerq18	2.31	1.120	631
cerq19	3.77	1.067	631
cerq20	3.37	1.241	631
cerq21	3.87	1.163	631
cerq22	2.89	1.250	631
cerq23	3.81	1.098	631
cerq24	3.63	1.113	631
cerq25	3.44	1.156	631
cerq26	2.24	1.280	631
cerq27	2.84	1.189	631
cerq28	2.97	1.154	631
cerq29	3.60	1.138	631
cerq30	3.71	1.112	631
cerq31	3.35	1.192	631
cerq32	3.90	1.058	631
cerq33	3.71	1.128	631
cerq34	3.59	1.165	631
cerq35	2.85	1.208	631
cerq36	2.28	1.142	631

Inter-Item Correlation Matrix

	cerq1	cerq2	cerq3	cerq4	cerq5	cerq6	cerq7	cerq8
cerq1	1.000	.191	.295	-.043	.041	.000	.029	.218
cerq2	.191	1.000	.455	.236	.389	.374	.298	.043
cerq3	.295	.455	1.000	.202	.375	.320	.192	.019
cerq4	-.043	.236	.202	1.000	.279	.326	.327	.163
cerq5	.041	.389	.375	.279	1.000	.615	.419	-.056
cerq6	.000	.374	.320	.326	.615	1.000	.382	-.059
cerq7	.029	.298	.192	.327	.419	.382	1.000	.160
cerq8	.218	.043	.019	.163	-.056	-.059	.160	1.000
cerq9	.243	.038	.000	.097	-.097	-.129	.029	.377
cerq10	.527	.145	.188	-.016	.095	.077	.057	.120
cerq11	.048	.621	.349	.257	.458	.425	.400	-.021
cerq12	.259	.348	.532	.180	.346	.375	.263	.073
cerq13	.033	.130	.113	.490	.198	.204	.334	.165
cerq14	-.054	.349	.333	.288	.527	.508	.322	-.083
cerq15	-.072	.333	.262	.332	.472	.601	.369	-.093
cerq16	.071	.221	.130	.263	.312	.264	.477	.172
cerq17	.329	.097	.195	.042	-.033	-.101	.103	.438
cerq18	.144	.049	.008	.113	-.099	-.125	.040	.337
cerq19	.312	.243	.353	.077	.330	.328	.271	.094
cerq20	.144	.276	.231	.141	.134	.115	.171	.101
cerq21	.176	.355	.455	.159	.350	.362	.183	.037
cerq22	.006	.119	.034	.440	.079	.087	.183	.181
cerq23	.096	.176	.249	.172	.397	.394	.215	.010
cerq24	-.078	.236	.140	.286	.363	.447	.337	-.117
cerq25	.016	.223	.121	.301	.288	.304	.400	.036
cerq26	.181	.008	-.005	.087	-.139	-.163	-.021	.488
cerq27	.214	.148	.163	.168	.025	.029	.151	.300
cerq28	.571	.176	.188	.011	.031	-.008	.100	.180
cerq29	.167	.440	.343	.204	.266	.235	.248	.099
cerq30	.221	.300	.474	.158	.289	.274	.193	.074
cerq31	-.105	.163	.049	.480	.190	.292	.230	.077
cerq32	.028	.320	.300	.270	.531	.471	.294	-.004
cerq33	-.139	.235	.113	.355	.399	.478	.396	-.072
cerq34	.053	.218	.155	.240	.328	.319	.507	.085
cerq35	.299	.040	.158	.033	.010	-.121	.043	.366
cerq36	.134	-.022	-.067	.105	-.124	-.170	.007	.318

Inter-Item Correlation Matrix

	cerq9	cerq10	cerq11	cerq12	cerq13	cerq14	cerq15	cerq16
cerq1	.243	.527	.048	.259	.033	-.054	-.072	.071
cerq2	.038	.145	.621	.348	.130	.349	.333	.221
cerq3	.000	.188	.349	.532	.113	.333	.262	.130
cerq4	.097	-.016	.257	.180	.490	.288	.332	.263
cerq5	-.097	.095	.458	.346	.198	.527	.472	.312
cerq6	-.129	.077	.425	.375	.204	.508	.601	.264
cerq7	.029	.057	.400	.263	.334	.322	.369	.477
cerq8	.377	.120	-.021	.073	.165	-.083	-.093	.172
cerq9	1.000	.041	-.055	-.003	.099	-.108	-.134	.088
cerq10	.041	1.000	.165	.234	.069	-.032	.014	.024
cerq11	-.055	.165	1.000	.411	.224	.428	.420	.282
cerq12	-.003	.234	.411	1.000	.165	.378	.304	.158
cerq13	.099	.069	.224	.165	1.000	.231	.230	.300
cerq14	-.108	-.032	.428	.378	.231	1.000	.601	.323
cerq15	-.134	.014	.420	.304	.230	.601	1.000	.335
cerq16	.088	.024	.282	.158	.300	.323	.335	1.000
cerq17	.273	.251	.059	.237	.108	-.025	-.062	.147
cerq18	.651	.054	-.037	-.004	.154	-.077	-.093	.144
cerq19	.014	.301	.286	.369	.116	.347	.247	.278
cerq20	.073	.117	.307	.197	.142	.216	.093	.200
cerq21	-.028	.171	.345	.527	.155	.396	.326	.233
cerq22	.117	.020	.109	.026	.542	.159	.140	.229
cerq23	.014	.138	.238	.298	.100	.359	.356	.209
cerq24	-.104	.014	.300	.179	.318	.425	.495	.298
cerq25	.005	.106	.264	.146	.302	.270	.323	.445
cerq26	.344	.153	-.065	.034	.144	-.111	-.117	.071
cerq27	.427	.102	.085	.220	.198	.035	.035	.185
cerq28	.161	.557	.185	.213	.068	-.045	-.063	.110
cerq29	.068	.206	.487	.376	.205	.271	.272	.252
cerq30	.026	.201	.382	.491	.150	.301	.250	.207
cerq31	.064	-.035	.185	.025	.516	.248	.301	.287
cerq32	-.052	.107	.353	.341	.193	.498	.436	.317
cerq33	-.116	-.029	.302	.153	.305	.464	.552	.340
cerq34	.084	.050	.271	.175	.285	.284	.337	.606
cerq35	.321	.266	.024	.208	.137	-.069	-.154	.111
cerq36	.661	.068	-.055	-.021	.138	-.150	-.107	.121

Inter-Item Correlation Matrix

	cerq17	cerq18	cerq19	cerq20	cerq21	cerq22	cerq23	cerq24
cerq1	.329	.144	.312	.144	.176	.006	.096	-.078
cerq2	.097	.049	.243	.276	.355	.119	.176	.236
cerq3	.195	.008	.353	.231	.455	.034	.249	.140
cerq4	.042	.113	.077	.141	.159	.440	.172	.286
cerq5	-.033	-.099	.330	.134	.350	.079	.397	.363
cerq6	-.101	-.125	.328	.115	.362	.087	.394	.447
cerq7	.103	.040	.271	.171	.183	.183	.215	.337
cerq8	.438	.337	.094	.101	.037	.181	.010	-.117
cerq9	.273	.651	.014	.073	-.028	.117	.014	-.104
cerq10	.251	.054	.301	.117	.171	.020	.138	.014
cerq11	.059	-.037	.286	.307	.345	.109	.238	.300
cerq12	.237	-.004	.369	.197	.527	.026	.298	.179
cerq13	.108	.154	.116	.142	.155	.542	.100	.318
cerq14	-.025	-.077	.347	.216	.396	.159	.359	.425
cerq15	-.062	-.093	.247	.093	.326	.140	.356	.495
cerq16	.147	.144	.278	.200	.233	.229	.209	.298
cerq17	1.000	.353	.269	.222	.205	.107	.077	-.179
cerq18	.353	1.000	.064	.124	.000	.215	-.003	-.069
cerq19	.269	.064	1.000	.236	.438	.005	.327	.181
cerq20	.222	.124	.236	1.000	.240	.145	.081	.070
cerq21	.205	.000	.438	.240	1.000	.065	.386	.233
cerq22	.107	.215	.005	.145	.065	1.000	.117	.300
cerq23	.077	-.003	.327	.081	.386	.117	1.000	.341
cerq24	-.179	-.069	.181	.070	.233	.300	.341	1.000
cerq25	-.012	.035	.209	.142	.201	.316	.294	.532
cerq26	.428	.405	-.014	.137	-.024	.282	-.002	-.109
cerq27	.303	.486	.251	.216	.203	.206	.189	.057
cerq28	.291	.152	.330	.177	.178	.043	.114	.006
cerq29	.185	.049	.278	.360	.369	.182	.252	.240
cerq30	.235	.004	.359	.229	.544	.028	.315	.155
cerq31	-.007	.100	.085	.067	.113	.553	.167	.393
cerq32	.017	-.069	.326	.109	.391	.125	.507	.404
cerq33	-.191	-.075	.171	.079	.205	.260	.320	.674
cerq34	.057	.083	.252	.159	.178	.217	.292	.399
cerq35	.609	.345	.189	.176	.153	.112	.104	-.196
cerq36	.290	.662	-.041	.093	-.017	.201	.001	-.037

Inter-Item Correlation Matrix

	cerq25	cerq26	cerq27	cerq28	cerq29	cerq30	cerq31	cerq32
cerq1	.016	.181	.214	.571	.167	.221	-.105	.028
cerq2	.223	.008	.148	.176	.440	.300	.163	.320
cerq3	.121	-.005	.163	.188	.343	.474	.049	.300
cerq4	.301	.087	.168	.011	.204	.158	.480	.270
cerq5	.288	-.139	.025	.031	.266	.289	.190	.531
cerq6	.304	-.163	.029	-.008	.235	.274	.292	.471
cerq7	.400	-.021	.151	.100	.248	.193	.230	.294
cerq8	.036	.488	.300	.180	.099	.074	.077	-.004
cerq9	.005	.344	.427	.161	.068	.026	.064	-.052
cerq10	.106	.153	.102	.557	.206	.201	-.035	.107
cerq11	.264	-.065	.085	.185	.487	.382	.185	.353
cerq12	.146	.034	.220	.213	.376	.491	.025	.341
cerq13	.302	.144	.198	.068	.205	.150	.516	.193
cerq14	.270	-.111	.035	-.045	.271	.301	.248	.498
cerq15	.323	-.117	.035	-.063	.272	.250	.301	.436
cerq16	.445	.071	.185	.110	.252	.207	.287	.317
cerq17	-.012	.428	.303	.291	.185	.235	-.007	.017
cerq18	.035	.405	.486	.152	.049	.004	.100	-.069
cerq19	.209	-.014	.251	.330	.278	.359	.085	.326
cerq20	.142	.137	.216	.177	.360	.229	.067	.109
cerq21	.201	-.024	.203	.178	.369	.544	.113	.391
cerq22	.316	.282	.206	.043	.182	.028	.553	.125
cerq23	.294	-.002	.189	.114	.252	.315	.167	.507
cerq24	.532	-.109	.057	.006	.240	.155	.393	.404
cerq25	1.000	.023	.140	.082	.240	.159	.337	.349
cerq26	.023	1.000	.335	.232	.080	.063	.087	-.051
cerq27	.140	.335	1.000	.237	.216	.234	.154	.079
cerq28	.082	.232	.237	1.000	.282	.260	-.008	.073
cerq29	.240	.080	.216	.282	1.000	.436	.216	.330
cerq30	.159	.063	.234	.260	.436	1.000	.167	.391
cerq31	.337	.087	.154	-.008	.216	.167	1.000	.317
cerq32	.349	-.051	.079	.073	.330	.391	.317	1.000
cerq33	.466	-.129	.054	-.010	.225	.148	.429	.494
cerq34	.536	.013	.181	.149	.324	.222	.350	.390
cerq35	-.030	.452	.374	.354	.207	.284	.007	.050
cerq36	.050	.370	.448	.158	.036	.039	.113	-.076

Inter-Item Correlation Matrix

	cerq33	cerq34	cerq35	cerq36
cerq1	-.139	.053	.299	.134
cerq2	.235	.218	.040	-.022
cerq3	.113	.155	.158	-.067
cerq4	.355	.240	.033	.105
cerq5	.399	.328	.010	-.124
cerq6	.478	.319	-.121	-.170
cerq7	.396	.507	.043	.007
cerq8	-.072	.085	.366	.318
cerq9	-.116	.084	.321	.661
cerq10	-.029	.050	.266	.068
cerq11	.302	.271	.024	-.055
cerq12	.153	.175	.208	-.021
cerq13	.305	.285	.137	.138
cerq14	.464	.284	-.069	-.150
cerq15	.552	.337	-.154	-.107
cerq16	.340	.606	.111	.121
cerq17	-.191	.057	.609	.290
cerq18	-.075	.083	.345	.662
cerq19	.171	.252	.189	-.041
cerq20	.079	.159	.176	.093
cerq21	.205	.178	.153	-.017
cerq22	.260	.217	.112	.201
cerq23	.320	.292	.104	.001
cerq24	.674	.399	-.196	-.037
cerq25	.466	.536	-.030	.050
cerq26	-.129	.013	.452	.370
cerq27	.054	.181	.374	.448
cerq28	-.010	.149	.354	.158
cerq29	.225	.324	.207	.036
cerq30	.148	.222	.284	.039
cerq31	.429	.350	.007	.113
cerq32	.494	.390	.050	-.076
cerq33	1.000	.464	-.167	-.046
cerq34	.464	1.000	.090	.109
cerq35	-.167	.090	1.000	.388
cerq36	-.046	.109	.388	1.000

Inter-Item Covariance Matrix

	cerq1	cerq2	cerq3	cerq4	cerq5	cerq6	cerq7	cerq8
cerq1	1.522	.262	.402	-.064	.051	.000	.041	.343
cerq2	.262	1.234	.558	.313	.438	.436	.383	.060
cerq3	.402	.558	1.220	.267	.420	.371	.245	.027
cerq4	-.064	.313	.267	1.423	.337	.409	.451	.248
cerq5	.051	.438	.420	.337	1.027	.654	.491	-.072
cerq6	.000	.436	.371	.409	.654	1.102	.464	-.079
cerq7	.041	.383	.245	.451	.491	.464	1.335	.235
cerq8	.343	.060	.027	.248	-.072	-.079	.235	1.622
cerq9	.337	.047	.000	.130	-.111	-.152	.038	.538
cerq10	.753	.187	.240	-.023	.112	.093	.076	.178
cerq11	.062	.735	.410	.327	.495	.475	.492	-.028
cerq12	.360	.435	.660	.241	.394	.443	.341	.105
cerq13	.051	.179	.155	.728	.250	.266	.479	.261
cerq14	-.067	.389	.369	.344	.536	.534	.373	-.107
cerq15	-.100	.414	.323	.443	.535	.705	.477	-.133
cerq16	.110	.309	.180	.394	.397	.349	.693	.275
cerq17	.482	.128	.256	.060	-.040	-.126	.141	.663
cerq18	.199	.060	.010	.151	-.112	-.146	.052	.480
cerq19	.411	.288	.416	.098	.357	.368	.335	.127
cerq20	.221	.381	.317	.209	.169	.150	.245	.160
cerq21	.252	.458	.585	.221	.412	.442	.246	.055
cerq22	.009	.166	.047	.656	.100	.114	.265	.288
cerq23	.130	.215	.302	.225	.441	.454	.272	.014
cerq24	-.107	.292	.172	.380	.409	.522	.433	-.166
cerq25	.023	.287	.155	.415	.337	.368	.534	.053
cerq26	.285	.011	-.007	.132	-.180	-.220	-.031	.796
cerq27	.313	.195	.214	.239	.031	.037	.208	.454
cerq28	.813	.225	.239	.015	.036	-.010	.134	.264
cerq29	.234	.556	.431	.277	.307	.281	.326	.144
cerq30	.303	.371	.582	.210	.326	.320	.248	.105
cerq31	-.155	.216	.064	.683	.229	.365	.317	.117
cerq32	.037	.376	.350	.340	.569	.523	.359	-.005
cerq33	-.194	.294	.141	.478	.456	.565	.516	-.104
cerq34	.076	.283	.199	.333	.387	.390	.682	.127
cerq35	.446	.053	.211	.047	.012	-.154	.060	.563
cerq36	.188	-.028	-.085	.144	-.144	-.203	.010	.463

Inter-Item Covariance Matrix

	cerq9	cerq10	cerq11	cerq12	cerq13	cerq14	cerq15	cerq16
cerq1	.337	.753	.062	.360	.051	-.067	-.100	.110
cerq2	.047	.187	.735	.435	.179	.389	.414	.309
cerq3	.000	.240	.410	.660	.155	.369	.323	.180
cerq4	.130	-.023	.327	.241	.728	.344	.443	.394
cerq5	-.111	.112	.495	.394	.250	.536	.535	.397
cerq6	-.152	.093	.475	.443	.266	.534	.705	.349
cerq7	.038	.076	.492	.341	.479	.373	.477	.693
cerq8	.538	.178	-.028	.105	.261	-.107	-.133	.275
cerq9	1.259	.054	-.065	-.004	.138	-.122	-.168	.124
cerq10	.054	1.341	.204	.305	.099	-.037	.018	.035
cerq11	-.065	.204	1.133	.492	.297	.457	.500	.378
cerq12	-.004	.305	.492	1.263	.231	.426	.382	.223
cerq13	.138	.099	.297	.231	1.547	.289	.320	.469
cerq14	-.122	-.037	.457	.426	.289	1.006	.674	.407
cerq15	-.168	.018	.500	.382	.320	.674	1.250	.471
cerq16	.124	.035	.378	.223	.469	.407	.471	1.579
cerq17	.364	.346	.075	.316	.159	-.030	-.082	.220
cerq18	.818	.071	-.044	-.005	.214	-.086	-.117	.203
cerq19	.016	.372	.325	.443	.153	.372	.295	.372
cerq20	.102	.168	.406	.274	.219	.268	.129	.311
cerq21	-.037	.231	.427	.689	.224	.461	.424	.341
cerq22	.165	.029	.145	.036	.843	.200	.196	.359
cerq23	.018	.176	.278	.368	.137	.396	.437	.288
cerq24	-.130	.017	.355	.224	.440	.475	.615	.416
cerq25	.007	.142	.324	.189	.433	.313	.418	.647
cerq26	.494	.227	-.088	.049	.229	-.142	-.168	.114
cerq27	.569	.141	.108	.294	.292	.042	.047	.276
cerq28	.208	.744	.227	.276	.098	-.053	-.082	.160
cerq29	.087	.272	.590	.481	.290	.309	.347	.360
cerq30	.033	.259	.452	.614	.208	.336	.311	.290
cerq31	.086	-.048	.234	.033	.765	.296	.401	.430
cerq32	-.061	.131	.397	.405	.254	.528	.515	.421
cerq33	-.147	-.037	.363	.194	.428	.525	.697	.482
cerq34	.109	.067	.337	.230	.413	.332	.438	.887
cerq35	.435	.372	.031	.282	.206	-.084	-.208	.168
cerq36	.846	.090	-.067	-.027	.197	-.172	-.137	.174

Inter-Item Covariance Matrix

	cerq17	cerq18	cerq19	cerq20	cerq21	cerq22	cerq23	cerq24
cerq1	.482	.199	.411	.221	.252	.009	.130	-.107
cerq2	.128	.060	.288	.381	.458	.166	.215	.292
cerq3	.256	.010	.416	.317	.585	.047	.302	.172
cerq4	.060	.151	.098	.209	.221	.656	.225	.380
cerq5	-.040	-.112	.357	.169	.412	.100	.441	.409
cerq6	-.126	-.146	.368	.150	.442	.114	.454	.522
cerq7	.141	.052	.335	.245	.246	.265	.272	.433
cerq8	.663	.480	.127	.160	.055	.288	.014	-.166
cerq9	.364	.818	.016	.102	-.037	.165	.018	-.130
cerq10	.346	.071	.372	.168	.231	.029	.176	.017
cerq11	.075	-.044	.325	.406	.427	.145	.278	.355
cerq12	.316	-.005	.443	.274	.689	.036	.368	.224
cerq13	.159	.214	.153	.219	.224	.843	.137	.440
cerq14	-.030	-.086	.372	.268	.461	.200	.396	.475
cerq15	-.082	-.117	.295	.129	.424	.196	.437	.615
cerq16	.220	.203	.372	.311	.341	.359	.288	.416
cerq17	1.411	.469	.341	.327	.283	.158	.100	-.237
cerq18	.469	1.254	.076	.172	-.001	.301	-.004	-.086
cerq19	.341	.076	1.138	.313	.544	.007	.382	.215
cerq20	.327	.172	.313	1.541	.346	.225	.110	.096
cerq21	.283	-.001	.544	.346	1.352	.095	.493	.302
cerq22	.158	.301	.007	.225	.095	1.563	.160	.417
cerq23	.100	-.004	.382	.110	.493	.160	1.205	.416
cerq24	-.237	-.086	.215	.096	.302	.417	.416	1.238
cerq25	-.016	.045	.257	.204	.270	.457	.373	.684
cerq26	.651	.581	-.019	.217	-.036	.452	-.003	-.155
cerq27	.428	.647	.318	.319	.281	.306	.247	.075
cerq28	.398	.196	.407	.254	.238	.061	.144	.008
cerq29	.250	.062	.338	.509	.488	.260	.315	.304
cerq30	.310	.005	.426	.316	.704	.039	.385	.192
cerq31	-.010	.134	.108	.099	.156	.825	.219	.520
cerq32	.022	-.081	.368	.143	.480	.165	.588	.475
cerq33	-.256	-.095	.206	.110	.268	.367	.396	.845
cerq34	.078	.109	.314	.230	.241	.316	.374	.517
cerq35	.874	.466	.243	.263	.215	.169	.137	-.263
cerq36	.394	.847	-.050	.132	-.023	.287	.001	-.047

Inter-Item Covariance Matrix

	cerq25	cerq26	cerq27	cerq28	cerq29	cerq30	cerq31	cerq32
cerq1	.023	.285	.313	.813	.234	.303	-.155	.037
cerq2	.287	.011	.195	.225	.556	.371	.216	.376
cerq3	.155	-.007	.214	.239	.431	.582	.064	.350
cerq4	.415	.132	.239	.015	.277	.210	.683	.340
cerq5	.337	-.180	.031	.036	.307	.326	.229	.569
cerq6	.368	-.220	.037	-.010	.281	.320	.365	.523
cerq7	.534	-.031	.208	.134	.326	.248	.317	.359
cerq8	.053	.796	.454	.264	.144	.105	.117	-.005
cerq9	.007	.494	.569	.208	.087	.033	.086	-.061
cerq10	.142	.227	.141	.744	.272	.259	-.048	.131
cerq11	.324	-.088	.108	.227	.590	.452	.234	.397
cerq12	.189	.049	.294	.276	.481	.614	.033	.405
cerq13	.433	.229	.292	.098	.290	.208	.765	.254
cerq14	.313	-.142	.042	-.053	.309	.336	.296	.528
cerq15	.418	-.168	.047	-.082	.347	.311	.401	.515
cerq16	.647	.114	.276	.160	.360	.290	.430	.421
cerq17	-.016	.651	.428	.398	.250	.310	-.010	.022
cerq18	.045	.581	.647	.196	.062	.005	.134	-.081
cerq19	.257	-.019	.318	.407	.338	.426	.108	.368
cerq20	.204	.217	.319	.254	.509	.316	.099	.143
cerq21	.270	-.036	.281	.238	.488	.704	.156	.480
cerq22	.457	.452	.306	.061	.260	.039	.825	.165
cerq23	.373	-.003	.247	.144	.315	.385	.219	.588
cerq24	.684	-.155	.075	.008	.304	.192	.520	.475
cerq25	1.335	.033	.193	.110	.315	.204	.464	.426
cerq26	.033	1.638	.510	.343	.117	.089	.133	-.069
cerq27	.193	.510	1.414	.325	.292	.310	.218	.099
cerq28	.110	.343	.325	1.331	.371	.333	-.011	.089
cerq29	.315	.117	.292	.371	1.295	.552	.293	.398
cerq30	.204	.089	.310	.333	.552	1.237	.221	.460
cerq31	.464	.133	.218	-.011	.293	.221	1.421	.400
cerq32	.426	-.069	.099	.089	.398	.460	.400	1.119
cerq33	.607	-.186	.072	-.013	.289	.186	.577	.590
cerq34	.722	.019	.251	.201	.430	.287	.486	.481
cerq35	-.042	.698	.537	.493	.285	.382	.009	.064
cerq36	.066	.541	.609	.208	.047	.050	.153	-.091

Inter-Item Covariance Matrix

	cerq33	cerq34	cerq35	cerq36
cerq1	-.194	.076	.446	.188
cerq2	.294	.283	.053	-.028
cerq3	.141	.199	.211	-.085
cerq4	.478	.333	.047	.144
cerq5	.456	.387	.012	-.144
cerq6	.565	.390	-.154	-.203
cerq7	.516	.682	.060	.010
cerq8	-.104	.127	.563	.463
cerq9	-.147	.109	.435	.846
cerq10	-.037	.067	.372	.090
cerq11	.363	.337	.031	-.067
cerq12	.194	.230	.282	-.027
cerq13	.428	.413	.206	.197
cerq14	.525	.332	-.084	-.172
cerq15	.697	.438	-.208	-.137
cerq16	.482	.887	.168	.174
cerq17	-.256	.078	.874	.394
cerq18	-.095	.109	.466	.847
cerq19	.206	.314	.243	-.050
cerq20	.110	.230	.263	.132
cerq21	.268	.241	.215	-.023
cerq22	.367	.316	.169	.287
cerq23	.396	.374	.137	.001
cerq24	.845	.517	-.263	-.047
cerq25	.607	.722	-.042	.066
cerq26	-.186	.019	.698	.541
cerq27	.072	.251	.537	.609
cerq28	-.013	.201	.493	.208
cerq29	.289	.430	.285	.047
cerq30	.186	.287	.382	.050
cerq31	.577	.486	.009	.153
cerq32	.590	.481	.064	-.091
cerq33	1.272	.610	-.227	-.060
cerq34	.610	1.357	.127	.146
cerq35	-.227	.127	1.458	.535
cerq36	-.060	.146	.535	1.305

Item-Total Statistics

	Scale Mean if Item Deleted	Scale Variance if Item Deleted	Corrected Item-Total Correlation	Squared Multiple Correlation	Cronbach's Alpha if Item Deleted
cerq1	117.81	349.036	.282	.516	.893
cerq2	116.81	342.389	.485	.514	.890
cerq3	116.77	343.904	.450	.468	.890
cerq4	117.30	342.429	.447	.422	.890
cerq5	116.44	344.481	.480	.546	.890
cerq6	116.44	344.453	.462	.578	.890
cerq7	116.85	340.976	.498	.462	.889
cerq8	117.93	349.045	.271	.391	.893
cerq9	118.28	352.777	.226	.577	.894
cerq10	117.36	350.106	.280	.437	.893
cerq11	116.68	342.224	.513	.572	.889
cerq12	116.76	341.508	.501	.499	.889
cerq13	117.41	341.195	.453	.465	.890
cerq14	116.48	345.060	.470	.549	.890
cerq15	116.55	343.642	.451	.566	.890
cerq16	116.92	338.525	.508	.479	.889
cerq17	117.72	347.167	.339	.520	.892
cerq18	118.21	351.133	.267	.585	.893
cerq19	116.75	343.351	.483	.431	.890
cerq20	117.16	345.800	.352	.243	.892
cerq21	116.66	340.666	.502	.491	.889
cerq22	117.63	344.639	.374	.502	.891
cerq23	116.72	344.398	.441	.385	.890
cerq24	116.90	345.077	.417	.576	.891
cerq25	117.09	342.198	.469	.463	.890
cerq26	118.28	351.096	.226	.440	.894
cerq27	117.69	343.163	.431	.415	.890
cerq28	117.56	347.339	.347	.505	.892
cerq29	116.93	339.865	.534	.434	.889
cerq30	116.81	341.497	.507	.488	.889
cerq31	117.18	344.090	.408	.521	.891
cerq32	116.63	342.158	.519	.524	.889
cerq33	116.82	344.411	.427	.613	.891
cerq34	116.94	339.760	.523	.556	.889
cerq35	117.68	347.295	.329	.538	.892
cerq36	118.25	352.283	.233	.594	.894

Scale Statistics

Mean	Variance	Std. Deviation	N of Items
120.53	363.570	19.068	36

Intraclass Correlation Coefficient

	Intraclass Correlation[b]	95% Confidence Interval		F Test with True Value 0		
		Lower Bound	Upper Bound	Value	df1	df2
Single Measures	.189[a]	.171	.209	9.389	630	22050
Average Measures	.893[c]	.881	.905	9.389	630	22050

Intraclass Correlation Coefficient

	F Test ...
	Sig
Single Measures	.000
Average Measures	.000

Two-way mixed effects model where people effects are random and measures effects are fixed.

a. The estimator is the same, whether the interaction effect is present or not.

b. Type C intraclass correlation coefficients using a consistency definition-the between-measure variance is excluded from the denominator variance.

c. This estimate is computed assuming the interaction effect is absent, because it is not estimable otherwise.

```
RELIABILITY
  /VARIABLES=pss1 pss2 pss3 pss4 pss5 pss6 pss7 pss8 pss9 pss10
  /SCALE('ALL VARIABLES') ALL
  /MODEL=ALPHA
  /STATISTICS=DESCRIPTIVE SCALE CORR COV
  /SUMMARY=TOTAL
  /ICC=MODEL(MIXED) TYPE(CONSISTENCY) CIN=95 TESTVAL=0.
```

Reliability

Notes

Output Created		16-APR-2023 18:02:32
Comments		
Input	Data	D:\Psiho-An 6\Conferinta razboi\FearOfWar_database_1.sav
	Active Dataset	DataSet1
	Filter	<none>
	Weight	<none>
	Split File	<none>
	N of Rows in Working Data File	633
	Matrix Input	
Missing Value Handling	Definition of Missing	User-defined missing values are treated as missing.
	Cases Used	Statistics are based on all cases with valid data for all variables in the procedure.
Syntax		RELIABILITY /VARIABLES=pss1 pss2 pss3 pss4 pss5 pss6 pss7 pss8 pss9 pss10 /SCALE('ALL VARIABLES') ALL /MODEL=ALPHA /STATISTICS=DESCRIPTIVE SCALE CORR COV /SUMMARY=TOTAL /ICC=MODEL(MIXED) TYPE(CONSISTENCY) CIN=95 TESTVAL=0.
Resources	Processor Time	00:00:00.00
	Elapsed Time	00:00:00.00

```
[DataSet1] D:\Psiho-An 6\Conferinta razboi\FearOfWar_database_1.sav
```

Scale: ALL VARIABLES

Case Processing Summary

		N	%
Cases	Valid	633	100.0
	Excluded[a]	0	.0
	Total	633	100.0

a. Listwise deletion based on all variables in the procedure.

Reliability Statistics

Cronbach's Alpha	Cronbach's Alpha Based on Standardized Items	N of Items
.861	.859	10

Item Statistics

	Mean	Std. Deviation	N
pss1	2.18	1.048	633
pss2	2.05	1.172	633
pss3	2.51	1.103	633
pss4	1.46	1.018	633
pss5	1.69	1.010	633
pss6	2.07	1.158	633
pss7	1.66	.996	633
pss8	1.67	1.013	633
pss9	2.17	1.114	633
pss10	1.92	1.238	633

Inter-Item Correlation Matrix

	pss1	pss2	pss3	pss4	pss5	pss6	pss7	pss8
pss1	1.000	.576	.589	.114	.244	.360	.160	.239
pss2	.576	1.000	.578	.259	.394	.534	.318	.390
pss3	.589	.578	1.000	.147	.296	.438	.220	.280
pss4	.114	.259	.147	1.000	.527	.216	.464	.491
pss5	.244	.394	.296	.527	1.000	.285	.504	.550
pss6	.360	.534	.438	.216	.285	1.000	.206	.305
pss7	.160	.318	.220	.464	.504	.206	1.000	.592
pss8	.239	.390	.280	.491	.550	.305	.592	1.000
pss9	.563	.520	.520	.101	.241	.397	.172	.219
pss10	.523	.584	.526	.316	.378	.559	.270	.333

Inter-Item Correlation Matrix

	pss9	pss10
pss1	.563	.523
pss2	.520	.584
pss3	.520	.526
pss4	.101	.316
pss5	.241	.378
pss6	.397	.559
pss7	.172	.270
pss8	.219	.333
pss9	1.000	.568
pss10	.568	1.000

Inter-Item Covariance Matrix

	pss1	pss2	pss3	pss4	pss5	pss6	pss7	pss8
pss1	1.097	.707	.680	.121	.258	.437	.167	.253
pss2	.707	1.374	.746	.309	.466	.724	.371	.464
pss3	.680	.746	1.216	.165	.330	.560	.242	.312
pss4	.121	.309	.165	1.037	.542	.254	.471	.507
pss5	.258	.466	.330	.542	1.020	.333	.507	.563
pss6	.437	.724	.560	.254	.333	1.340	.237	.358
pss7	.167	.371	.242	.471	.507	.237	.993	.598
pss8	.253	.464	.312	.507	.563	.358	.598	1.027
pss9	.656	.679	.639	.115	.271	.512	.191	.248
pss10	.678	.848	.718	.398	.473	.802	.333	.417

Inter-Item Covariance Matrix

	pss9	pss10
pss1	.656	.678
pss2	.679	.848
pss3	.639	.718
pss4	.115	.398
pss5	.271	.473
pss6	.512	.802
pss7	.191	.333
pss8	.248	.417
pss9	1.241	.783
pss10	.783	1.533

Item-Total Statistics

	Scale Mean if Item Deleted	Scale Variance if Item Deleted	Corrected Item-Total Correlation	Squared Multiple Correlation	Cronbach's Alpha if Item Deleted
pss1	17.21	43.750	.571	.491	.848
pss2	17.33	40.761	.710	.550	.835
pss3	16.88	42.763	.609	.476	.845
pss4	17.93	45.961	.418	.381	.860
pss5	17.70	44.257	.557	.446	.849
pss6	17.32	42.990	.556	.391	.849
pss7	17.73	45.538	.464	.418	.856
pss8	17.71	44.296	.552	.479	.849
pss9	17.22	43.334	.558	.453	.849
pss10	17.47	40.332	.693	.545	.836

Scale Statistics

Mean	Variance	Std. Deviation	N of Items
19.39	52.763	7.264	10

Intraclass Correlation Coefficient

	Intraclass Correlation[b]	95% Confidence Interval		F Test with True Value 0		
		Lower Bound	Upper Bound	Value	df1	df2
Single Measures	.383[a]	.352	.415	7.195	632	5688
Average Measures	.861[c]	.844	.877	7.195	632	5688

Intraclass Correlation Coefficient

	F Test ...
	Sig
Single Measures	.000
Average Measures	.000

Two-way mixed effects model where people effects are random and measures effects are fixed.

a. The estimator is the same, whether the interaction effect is present or not.

b. Type C intraclass correlation coefficients using a consistency definition-the between-measure variance is excluded from the denominator variance.

c. This estimate is computed assuming the interaction effect is absent, because it is not estimable otherwise.

```
RELIABILITY
  /VARIABLES=brs1 brs2 brs3 brs4 brs5 brs6
  /SCALE('ALL VARIABLES') ALL
  /MODEL=ALPHA
  /STATISTICS=DESCRIPTIVE SCALE CORR COV
  /SUMMARY=TOTAL
```

```
/ICC=MODEL(MIXED) TYPE(CONSISTENCY) CIN=95 TESTVAL=0.
```

Reliability

Notes

Output Created		16-APR-2023 18:03:09
Comments		
Input	Data	D:\Psiho-An 6\Conferinta razboi\FearOfWar_database_1.sav
	Active Dataset	DataSet1
	Filter	<none>
	Weight	<none>
	Split File	<none>
	N of Rows in Working Data File	633
	Matrix Input	
Missing Value Handling	Definition of Missing	User-defined missing values are treated as missing.
	Cases Used	Statistics are based on all cases with valid data for all variables in the procedure.
Syntax		RELIABILITY /VARIABLES=brs1 brs2 brs3 brs4 brs5 brs6 /SCALE('ALL VARIABLES') ALL /MODEL=ALPHA /STATISTICS=DESCRIPTIVE SCALE CORR COV /SUMMARY=TOTAL /ICC=MODEL(MIXED) TYPE(CONSISTENCY) CIN=95 TESTVAL=0.
Resources	Processor Time	00:00:00.02
	Elapsed Time	00:00:00.01

```
[DataSet1] D:\Psiho-An 6\Conferinta razboi\FearOfWar_database_1.sav
```

Scale: ALL VARIABLES

Case Processing Summary

		N	%
Cases	Valid	633	100.0
	Excluded[a]	0	.0
	Total	633	100.0

a. Listwise deletion based on all variables in the procedure.

Reliability Statistics

Cronbach's Alpha	Cronbach's Alpha Based on Standardized Items	N of Items
.846	.847	6

Item Statistics

	Mean	Std. Deviation	N
brs1	3.43	1.140	633
brs2	3.09	1.101	633
brs3	3.28	1.123	633
brs4	3.17	1.091	633
brs5	3.24	1.094	633
brs6	3.02	1.141	633

Inter-Item Correlation Matrix

	brs1	brs2	brs3	brs4	brs5	brs6
brs1	1.000	.425	.504	.425	.541	.411
brs2	.425	1.000	.398	.613	.407	.516
brs3	.504	.398	1.000	.357	.566	.393
brs4	.425	.613	.357	1.000	.488	.644
brs5	.541	.407	.566	.488	1.000	.501
brs6	.411	.516	.393	.644	.501	1.000

Inter-Item Covariance Matrix

	brs1	brs2	brs3	brs4	brs5	brs6
brs1	1.299	.534	.645	.528	.675	.534
brs2	.534	1.212	.492	.736	.490	.648
brs3	.645	.492	1.260	.437	.695	.503
brs4	.528	.736	.437	1.191	.583	.801
brs5	.675	.490	.695	.583	1.197	.626
brs6	.534	.648	.503	.801	.626	1.302

Item-Total Statistics

	Scale Mean if Item Deleted	Scale Variance if Item Deleted	Corrected Item-Total Correlation	Squared Multiple Correlation	Cronbach's Alpha if Item Deleted
brs1	15.80	18.188	.600	.388	.826
brs2	16.14	18.305	.616	.437	.823
brs3	15.95	18.512	.574	.395	.831
brs4	16.06	17.954	.667	.541	.813
brs5	15.99	17.984	.661	.477	.815
brs6	16.20	17.792	.647	.479	.817

Scale Statistics

Mean	Variance	Std. Deviation	N of Items
19.23	25.318	5.032	6

Intraclass Correlation Coefficient

	Intraclass Correlation[b]	95% Confidence Interval		F Test with True Value 0		
		Lower Bound	Upper Bound	Value	df1	df2
Single Measures	.479[a]	.443	.515	6.508	632	3160
Average Measures	.846[c]	.827	.864	6.508	632	3160

Intraclass Correlation Coefficient

	F Test ...
	Sig
Single Measures	.000
Average Measures	.000

Two-way mixed effects model where people effects are random and measures effects are fixed.

a. The estimator is the same, whether the interaction effect is present or not.

b. Type C intraclass correlation coefficients using a consistency definition-the between-measure variance is excluded from the denominator variance.

c. This estimate is computed assuming the interaction effect is absent, because it is not estimable otherwise.

```
RELIABILITY
  /VARIABLES=si1 si2 si3 si4
  /SCALE('ALL VARIABLES') ALL
  /MODEL=ALPHA
  /STATISTICS=DESCRIPTIVE SCALE CORR COV
  /SUMMARY=TOTAL
  /ICC=MODEL(MIXED) TYPE(CONSISTENCY) CIN=95 TESTVAL=0.
```

Reliability

Notes

Output Created		16-APR-2023 18:03:48
Comments		
Input	Data	D:\Psiho-An 6\Conferinta razboi\FearOfWar_database_1.sav
	Active Dataset	DataSet1
	Filter	<none>
	Weight	<none>
	Split File	<none>
	N of Rows in Working Data File	633
	Matrix Input	
Missing Value Handling	Definition of Missing	User-defined missing values are treated as missing.
	Cases Used	Statistics are based on all cases with valid data for all variables in the procedure.
Syntax		RELIABILITY<br>/VARIABLES=si1 si2 si3 si4<br>/SCALE('ALL VARIABLES') ALL<br>/MODEL=ALPHA<br><br>/STATISTICS=DESCRIPTIVE SCALE CORR COV<br>/SUMMARY=TOTAL<br>/ICC=MODEL(MIXED) TYPE(CONSISTENCY) CIN=95 TESTVAL=0.
Resources	Processor Time	00:00:00.00
	Elapsed Time	00:00:00.00

```
[DataSet1] D:\Psiho-An 6\Conferinta razboi\FearOfWar_database_1.sav
```

Scale: ALL VARIABLES

Case Processing Summary

		N	%
Cases	Valid	633	100.0
	Excluded[a]	0	.0
	Total	633	100.0

a. Listwise deletion based on all variables in the procedure.

Reliability Statistics

Cronbach's Alpha	Cronbach's Alpha Based on Standardized Items	N of Items
.828	.829	4

Item Statistics

	Mean	Std. Deviation	N
si1	4.46	1.913	633
si2	4.22	1.870	633
si3	3.88	1.816	633
si4	3.78	1.868	633

Inter-Item Correlation Matrix

	si1	si2	si3	si4
si1	1.000	.551	.544	.471
si2	.551	1.000	.563	.535
si3	.544	.563	1.000	.623
si4	.471	.535	.623	1.000

Inter-Item Covariance Matrix

	si1	si2	si3	si4
si1	3.660	1.970	1.891	1.685
si2	1.970	3.496	1.913	1.869
si3	1.891	1.913	3.299	2.114
si4	1.685	1.869	2.114	3.490

Item-Total Statistics

	Scale Mean if Item Deleted	Scale Variance if Item Deleted	Corrected Item-Total Correlation	Squared Multiple Correlation	Cronbach's Alpha if Item Deleted
si1	11.88	22.077	.617	.391	.801
si2	12.12	21.828	.658	.435	.782
si3	12.46	21.695	.699	.499	.764
si4	12.56	22.003	.647	.445	.787

Scale Statistics

Mean	Variance	Std. Deviation	N of Items
16.34	36.829	6.069	4

Intraclass Correlation Coefficient

	Intraclass Correlation[b]	95% Confidence Interval		F Test with True Value 0		
		Lower Bound	Upper Bound	Value	df1	df2
Single Measures	.547[a]	.509	.585	5.830	632	1896
Average Measures	.828[c]	.806	.849	5.830	632	1896

Intraclass Correlation Coefficient

	F Test ...
	Sig
Single Measures	.000
Average Measures	.000

Two-way mixed effects model where people effects are random and measures effects are fixed.

a. The estimator is the same, whether the interaction effect is present or not.

b. Type C intraclass correlation coefficients using a consistency definition-the between-measure variance is excluded from the denominator variance.

c. This estimate is computed assuming the interaction effect is absent, because it is not estimable otherwise.

```
RELIABILITY
  /VARIABLES=ti1 ti2 ti3 ti4
  /SCALE('ALL VARIABLES') ALL
  /MODEL=ALPHA
  /STATISTICS=DESCRIPTIVE SCALE CORR COV
  /SUMMARY=TOTAL
  /ICC=MODEL(MIXED) TYPE(CONSISTENCY) CIN=95 TESTVAL=0.
```

Reliability

Notes

Output Created		16-APR-2023 18:04:15
Comments		
Input	Data	D:\Psiho-An 6\Conferinta razboi\FearOfWar_database_1.sav
	Active Dataset	DataSet1
	Filter	<none>
	Weight	<none>
	Split File	<none>
	N of Rows in Working Data File	633
	Matrix Input	
Missing Value Handling	Definition of Missing	User-defined missing values are treated as missing.
	Cases Used	Statistics are based on all cases with valid data for all variables in the procedure.
Syntax		RELIABILITY<br>/VARIABLES=ti1 ti2 ti3 ti4<br>/SCALE('ALL VARIABLES') ALL<br>/MODEL=ALPHA<br><br>/STATISTICS=DESCRIPTIVE SCALE CORR COV<br>/SUMMARY=TOTAL<br>/ICC=MODEL(MIXED) TYPE(CONSISTENCY) ...
Resources	Processor Time	00:00:00.00
	Elapsed Time	00:00:00.00

```
[DataSet1] D:\Psiho-An 6\Conferinta razboi\FearOfWar_database_1.sav
```

Scale: ALL VARIABLES

Case Processing Summary

		N	%
Cases	Valid	633	100.0
	Excluded[a]	0	.0
	Total	633	100.0

a. Listwise deletion based on all variables in the procedure.

Reliability Statistics

Cronbach's Alpha	Cronbach's Alpha Based on Standardized Items	N of Items
.846	.847	4

Item Statistics

	Mean	Std. Deviation	N
ti1	2.71	1.685	633
ti2	2.29	1.539	633
ti3	2.47	1.675	633
ti4	2.85	1.731	633

Inter-Item Correlation Matrix

	ti1	ti2	ti3	ti4
ti1	1.000	.608	.583	.550
ti2	.608	1.000	.594	.550
ti3	.583	.594	1.000	.602
ti4	.550	.550	.602	1.000

Inter-Item Covariance Matrix

	ti1	ti2	ti3	ti4
ti1	2.841	1.576	1.646	1.604
ti2	1.576	2.368	1.531	1.465
ti3	1.646	1.531	2.806	1.744
ti4	1.604	1.465	1.744	2.997

Item-Total Statistics

	Scale Mean if Item Deleted	Scale Variance if Item Deleted	Corrected Item-Total Correlation	Squared Multiple Correlation	Cronbach's Alpha if Item Deleted
ti1	7.61	17.654	.681	.471	.806
ti2	8.02	18.632	.688	.479	.804
ti3	7.84	17.496	.702	.494	.796
ti4	7.47	17.521	.664	.446	.814

Scale Statistics

Mean	Variance	Std. Deviation	N of Items
10.31	30.146	5.491	4

Intraclass Correlation Coefficient

	Intraclass Correlation[b]	95% Confidence Interval		F Test with True Value 0		
		Lower Bound	Upper Bound	Value	df1	df2
Single Measures	.579[a]	.542	.616	6.505	632	1896
Average Measures	.846[c]	.826	.865	6.505	632	1896

Intraclass Correlation Coefficient

	F Test ...
	Sig
Single Measures	.000
Average Measures	.000

Two-way mixed effects model where people effects are random and measures effects are fixed.

a. The estimator is the same, whether the interaction effect is present or not.

b. Type C intraclass correlation coefficients using a consistency definition-the between-measure variance is excluded from the denominator variance.

c. This estimate is computed assuming the interaction effect is absent, because it is not estimable otherwise.

```
RELIABILITY
  /VARIABLES=fn1 fn2
  /SCALE('ALL VARIABLES') ALL
  /MODEL=ALPHA
  /STATISTICS=DESCRIPTIVE SCALE CORR COV
  /SUMMARY=TOTAL
  /ICC=MODEL(MIXED) TYPE(CONSISTENCY) CIN=95 TESTVAL=0.
```

Reliability

Notes

Output Created		16-APR-2023 18:04:49
Comments		
Input	Data	D:\Psiho-An 6\Conferinta razboi\FearOfWar_database_1.sav
	Active Dataset	DataSet1
	Filter	<none>
	Weight	<none>
	Split File	<none>
	N of Rows in Working Data File	633
	Matrix Input	
Missing Value Handling	Definition of Missing	User-defined missing values are treated as missing.
	Cases Used	Statistics are based on all cases with valid data for all variables in the procedure.
Syntax		RELIABILITY /VARIABLES=fn1 fn2 /SCALE('ALL VARIABLES') ALL /MODEL=ALPHA /STATISTICS=DESCRIPTIVE SCALE CORR COV /SUMMARY=TOTAL /ICC=MODEL(MIXED) TYPE(CONSISTENCY) CIN=95 TESTVAL=0.
Resources	Processor Time	00:00:00.00
	Elapsed Time	00:00:00.00

```
[DataSet1] D:\Psiho-An 6\Conferinta razboi\FearOfWar_database_1.sav
```

Scale: ALL VARIABLES

Case Processing Summary

		N	%
Cases	Valid	633	100.0
	Excluded[a]	0	.0
	Total	633	100.0

a. Listwise deletion based on all variables in the procedure.

Reliability Statistics

Cronbach's Alpha	Cronbach's Alpha Based on Standardized Items	N of Items
.929	.929	2

Item Statistics

	Mean	Std. Deviation	N
fn1	1.66	1.382	633
fn2	1.69	1.367	633

Inter-Item Correlation Matrix

	fn1	fn2
fn1	1.000	.868
fn2	.868	1.000

Inter-Item Covariance Matrix

	fn1	fn2
fn1	1.909	1.640
fn2	1.640	1.870

Item-Total Statistics

	Scale Mean if Item Deleted	Scale Variance if Item Deleted	Corrected Item-Total Correlation	Squared Multiple Correlation	Cronbach's Alpha if Item Deleted
fn1	1.69	1.870	.868	.753	.
fn2	1.66	1.909	.868	.753	.

Scale Statistics

Mean	Variance	Std. Deviation	N of Items
3.35	7.059	2.657	2

Intraclass Correlation Coefficient

	Intraclass Correlation[b]	95% Confidence Interval		F Test with True Value 0		
		Lower Bound	Upper Bound	Value	df1	df2
Single Measures	.868[a]	.847	.886	14.146	632	632
Average Measures	.929[c]	.917	.940	14.146	632	632

Intraclass Correlation Coefficient

	F Test ...
	Sig
Single Measures	.000
Average Measures	.000

Two-way mixed effects model where people effects are random and measures effects are fixed.

a. The estimator is the same, whether the interaction effect is present or not.

b. Type C intraclass correlation coefficients using a consistency definition-the between-measure variance is excluded from the denominator variance.

c. This estimate is computed assuming the interaction effect is absent, because it is not estimable otherwise.

```
RELIABILITY
  /VARIABLES=fow1 fow2 fow3 fow4 fow5 fow6 fow7 fow8 fow9 fow10 fow11 fow12 fow13
  /SCALE('ALL VARIABLES') ALL
  /MODEL=ALPHA
  /STATISTICS=DESCRIPTIVE SCALE CORR COV
  /SUMMARY=TOTAL
  /ICC=MODEL(MIXED) TYPE(CONSISTENCY) CIN=95 TESTVAL=0.
```

Reliability

Notes

Output Created		16-APR-2023 18:05:35
Comments		
Input	Data	D:\Psiho-An 6\Conferinta razboi\FearOfWar_database_1.sav
	Active Dataset	DataSet1
	Filter	<none>
	Weight	<none>
	Split File	<none>
	N of Rows in Working Data File	633
	Matrix Input	
Missing Value Handling	Definition of Missing	User-defined missing values are treated as missing.
	Cases Used	Statistics are based on all cases with valid data for all variables in the procedure.
Syntax		RELIABILITY /VARIABLES=fow1 fow2 fow3 fow4 fow5 fow6 fow7 fow8 fow9 fow10 fow11 fow12 fow13 /SCALE('ALL VARIABLES') ALL /MODEL=ALPHA /STATISTICS=DESCRIPTIVE SCALE CORR COV /SUMMARY=TOTAL /ICC=MODEL(MIXED) TYPE(CONSISTENCY) CIN=95 TESTVAL=0.
Resources	Processor Time	00:00:00.00
	Elapsed Time	00:00:00.00

```
[DataSet1] D:\Psiho-An 6\Conferinta razboi\FearOfWar_database_1.sav
```

Scale: ALL VARIABLES

Case Processing Summary

		N	%
Cases	Valid	633	100.0
	Excluded[a]	0	.0
	Total	633	100.0

a. Listwise deletion based on all variables in the procedure.

Reliability Statistics

Cronbach's Alpha	Cronbach's Alpha Based on Standardized Items	N of Items
.922	.921	13

Item Statistics

	Mean	Std. Deviation	N
fow1	3.71	1.184	633
fow2	3.74	1.163	633
fow3	3.58	1.206	633
fow4	3.55	1.244	633
fow5	3.61	1.230	633
fow6	3.90	1.148	633
fow7	3.64	1.298	633
fow8	2.62	1.366	633
fow9	2.36	1.349	633
fow10	2.45	1.371	633
fow11	1.94	1.214	633
fow12	2.26	1.312	633
fow13	2.19	1.302	633

Inter-Item Correlation Matrix

	fow1	fow2	fow3	fow4	fow5	fow6	fow7	fow8
fow1	1.000	.729	.639	.590	.609	.573	.553	.334
fow2	.729	1.000	.762	.683	.629	.681	.607	.372
fow3	.639	.762	1.000	.715	.649	.635	.608	.398
fow4	.590	.683	.715	1.000	.670	.630	.635	.404
fow5	.609	.629	.649	.670	1.000	.611	.573	.446
fow6	.573	.681	.635	.630	.611	1.000	.597	.280
fow7	.553	.607	.608	.635	.573	.597	1.000	.448
fow8	.334	.372	.398	.404	.446	.280	.448	1.000
fow9	.316	.321	.324	.358	.389	.221	.367	.769
fow10	.352	.351	.355	.358	.423	.246	.393	.725
fow11	.209	.152	.203	.213	.235	.066	.237	.558
fow12	.297	.301	.347	.331	.386	.181	.345	.693
fow13	.288	.283	.294	.309	.350	.174	.326	.623

Inter-Item Correlation Matrix

	fow9	fow10	fow11	fow12	fow13
fow1	.316	.352	.209	.297	.288
fow2	.321	.351	.152	.301	.283
fow3	.324	.355	.203	.347	.294
fow4	.358	.358	.213	.331	.309
fow5	.389	.423	.235	.386	.350
fow6	.221	.246	.066	.181	.174
fow7	.367	.393	.237	.345	.326
fow8	.769	.725	.558	.693	.623
fow9	1.000	.806	.642	.734	.697
fow10	.806	1.000	.634	.789	.731
fow11	.642	.634	1.000	.733	.713
fow12	.734	.789	.733	1.000	.752
fow13	.697	.731	.713	.752	1.000

Inter-Item Covariance Matrix

	fow1	fow2	fow3	fow4	fow5	fow6	fow7	fow8
fow1	1.403	1.004	.913	.869	.887	.779	.850	.540
fow2	1.004	1.353	1.070	.988	.900	.909	.916	.591
fow3	.913	1.070	1.456	1.073	.963	.880	.952	.656
fow4	.869	.988	1.073	1.549	1.025	.900	1.025	.687
fow5	.887	.900	.963	1.025	1.513	.862	.915	.749
fow6	.779	.909	.880	.900	.862	1.317	.888	.439
fow7	.850	.916	.952	1.025	.915	.888	1.684	.794
fow8	.540	.591	.656	.687	.749	.439	.794	1.865
fow9	.505	.504	.528	.602	.645	.342	.643	1.416
fow10	.571	.559	.587	.611	.714	.386	.700	1.357
fow11	.301	.214	.297	.322	.351	.092	.373	.924
fow12	.462	.460	.549	.540	.623	.272	.588	1.243
fow13	.444	.429	.462	.500	.561	.260	.551	1.108

Inter-Item Covariance Matrix

	fow9	fow10	fow11	fow12	fow13
fow1	.505	.571	.301	.462	.444
fow2	.504	.559	.214	.460	.429
fow3	.528	.587	.297	.549	.462
fow4	.602	.611	.322	.540	.500
fow5	.645	.714	.351	.623	.561
fow6	.342	.386	.092	.272	.260
fow7	.643	.700	.373	.588	.551
fow8	1.416	1.357	.924	1.243	1.108
fow9	1.819	1.490	1.051	1.299	1.225
fow10	1.490	1.881	1.055	1.420	1.305
fow11	1.051	1.055	1.473	1.167	1.127
fow12	1.299	1.420	1.167	1.722	1.284
fow13	1.225	1.305	1.127	1.284	1.695

Item-Total Statistics

	Scale Mean if Item Deleted	Scale Variance if Item Deleted	Corrected Item-Total Correlation	Squared Multiple Correlation	Cronbach's Alpha if Item Deleted
fow1	35.85	121.158	.623	.586	.917
fow2	35.81	120.373	.669	.721	.915
fow3	35.97	119.501	.677	.679	.915
fow4	36.01	118.981	.674	.637	.915
fow5	35.94	118.908	.686	.589	.915
fow6	35.66	123.478	.550	.576	.919
fow7	35.92	118.740	.650	.532	.916
fow8	36.93	115.942	.714	.663	.913
fow9	37.20	116.497	.704	.742	.914
fow10	37.11	115.421	.730	.757	.913
fow11	37.61	122.794	.541	.620	.920
fow12	37.30	117.278	.697	.747	.914
fow13	37.37	118.606	.653	.667	.916

Scale Statistics

Mean	Variance	Std. Deviation	N of Items
39.56	138.814	11.782	13

Intraclass Correlation Coefficient

	Intraclass Correlation[b]	95% Confidence Interval		F Test with True Value 0		
		Lower Bound	Upper Bound	Value	df1	df2
Single Measures	.475[a]	.444	.506	12.750	632	7584
Average Measures	.922[c]	.912	.930	12.750	632	7584

Intraclass Correlation Coefficient

	F Test ...
	Sig
Single Measures	.000
Average Measures	.000

Two-way mixed effects model where people effects are random and measures effects are fixed.

a. The estimator is the same, whether the interaction effect is present or not.

b. Type C intraclass correlation coefficients using a consistency definition-the between-measure variance is excluded from the denominator variance.

c. This estimate is computed assuming the interaction effect is absent, because it is not estimable otherwise.

```
FREQUENCIES VARIABLES=fearofwar fake_news information_strain information_overload brs_total pss
cerq_rumination cerq_acceptance cerq_selfblame sontus_total ghq_total
  /STATISTICS=STDDEV MINIMUM MAXIMUM MEAN MEDIAN MODE SKEWNESS SESKEW KURTOSIS SEKURT
  /HISTOGRAM NORMAL
  /ORDER=ANALYSIS.
```

Frequencies

Notes

Output Created		16-APR-2023 18:10:18
Comments		
Input	Data	D:\Psiho-An 6\Conferinta razboi\FearOfWar_database_1.sav
	Active Dataset	DataSet1
	Filter	<none>
	Weight	<none>
	Split File	<none>
	N of Rows in Working Data File	633
Missing Value Handling	Definition of Missing	User-defined missing values are treated as missing.
	Cases Used	Statistics are based on all cases with valid data.

Notes

Syntax		FREQUENCIES VARIABLES=fearofwar fake_news information_strain information_overload brs_total pss_total cerq_blamingothers cerq_catastrophizing cerq_puttingintoperspectiv e cerq_positivereappraisal cerq_refocusonplanning cerq_positiverefocusing cerq_rumination cerq_acceptance cerq_selfblame sontus_total ghq_total /STATISTICS=STDDEV MINIMUM MAXIMUM MEAN MEDIAN MODE SKEWNESS SESKEW KURTOSIS SEKURT /HISTOGRAM NORMAL /ORDER=ANALYSIS.
Resources	Processor Time	00:00:03.16
	Elapsed Time	00:00:02.71

```
[DataSet1] D:\Psiho-An 6\Conferinta razboi\FearOfWar_database_1.sav
```

Statistics

		fearofwar	fake_news	information_strain	information_overload	brs_total	pss_total
N	Valid	633	633	633	633	633	633
	Missing	0	0	0	0	0	0
Mean		3.04	3.35	10.31	16.34	3.20	19.39
Median		3.00	2.00	9.00	17.00	3.17	20.00
Mode		3	2	4	16	3	20
Std. Deviation		.906	2.657	5.491	6.069	.839	7.264
Skewness		-.105	1.849	.638	-.181	.018	.068
Std. Error of Skewness		.097	.097	.097	.097	.097	.097
Kurtosis		-.227	2.136	-.484	-.393	-.021	.019
Std. Error of Kurtosis		.194	.194	.194	.194	.194	.194
Minimum		1	2	4	4	1	0
Maximum		5	14	28	28	5	40

Statistics

		cerq_blaming others	cerq_catastro phizing	cerq_puttingin toperspective	cerq_positiver eappraisal
N	Valid	631	633	633	633
	Missing	2	0	0	0
Mean		9.67	10.49	14.30	15.40
Median		9.00	10.00	15.00	16.00
Mode		8	12	15[a]	20
Std. Deviation		3.729	3.824	3.728	3.567
Skewness		.397	.257	-.408	-.638
Std. Error of Skewness		.097	.097	.097	.097
Kurtosis		-.446	-.606	-.304	-.003
Std. Error of Kurtosis		.194	.194	.194	.194
Minimum		4	4	4	4
Maximum		20	20	20	20

Statistics

		cerq_refocuso nplanning	cerq_positiver efocusing	cerq_ruminati on	cerq_accepta nce
N	Valid	633	633	633	633
	Missing	0	0	0	0
Mean		15.84	12.58	15.09	14.52
Median		16.00	13.00	15.00	14.00
Mode		16	12	20	14
Std. Deviation		3.234	3.869	3.569	3.398
Skewness		-.764	-.133	-.497	-.272
Std. Error of Skewness		.097	.097	.097	.097
Kurtosis		.386	-.568	-.181	-.285
Std. Error of Kurtosis		.194	.194	.194	.194
Minimum		4	4	4	4
Maximum		20	20	20	20

Statistics

		cerq_selfblame	sontus_total	ghq_total
N	Valid	633	633	633
	Missing	0	0	0
Mean		12.61	12.97	16.17
Median		13.00	13.00	16.00
Mode		13	13	12
Std. Deviation		3.522	4.066	8.128
Skewness		-.012	.070	.287
Std. Error of Skewness		.097	.097	.097
Kurtosis		-.396	-.572	-.552
Std. Error of Kurtosis		.194	.194	.194
Minimum		4	5	0
Maximum		20	23	36

a. Multiple modes exist. The smallest value is shown

Frequency Table

fearofwar

		Frequency	Percent	Valid Percent	Cumulative Percent
Valid	1	18	2.8	2.8	2.8
	1	1	.2	.2	3.0
	1	4	.6	.6	3.6
	1	3	.5	.5	4.1
	1	7	1.1	1.1	5.2
	1	4	.6	.6	5.8
	2	6	.9	.9	6.8
	2	5	.8	.8	7.6
	2	2	.3	.3	7.9
	2	6	.9	.9	8.8
	2	12	1.9	1.9	10.7
	2	6	.9	.9	11.7
	2	9	1.4	1.4	13.1
	2	14	2.2	2.2	15.3
	2	12	1.9	1.9	17.2
	2	7	1.1	1.1	18.3
	2	18	2.8	2.8	21.2
	2	15	2.4	2.4	23.5
	2	14	2.2	2.2	25.8

fearofwar

	Frequency	Percent	Valid Percent	Cumulative Percent
3	16	2.5	2.5	28.3
3	19	3.0	3.0	31.3
3	20	3.2	3.2	34.4
3	19	3.0	3.0	37.4
3	25	3.9	3.9	41.4
3	13	2.1	2.1	43.4
3	45	7.1	7.1	50.6
3	26	4.1	4.1	54.7
3	22	3.5	3.5	58.1
3	20	3.2	3.2	61.3
3	15	2.4	2.4	63.7
3	25	3.9	3.9	67.6
3	16	2.5	2.5	70.1
4	19	3.0	3.0	73.1
4	10	1.6	1.6	74.7
4	14	2.2	2.2	76.9
4	20	3.2	3.2	80.1
4	15	2.4	2.4	82.5
4	13	2.1	2.1	84.5
4	8	1.3	1.3	85.8
4	16	2.5	2.5	88.3
4	10	1.6	1.6	89.9
4	3	.5	.5	90.4
4	8	1.3	1.3	91.6
4	7	1.1	1.1	92.7
4	5	.8	.8	93.5
5	5	.8	.8	94.3
5	7	1.1	1.1	95.4
5	8	1.3	1.3	96.7
5	3	.5	.5	97.2
5	6	.9	.9	98.1
5	2	.3	.3	98.4
5	10	1.6	1.6	100.0
Total	633	100.0	100.0	

fake_news

		Frequency	Percent	Valid Percent	Cumulative Percent
Valid	2	469	74.1	74.1	74.1
	3	19	3.0	3.0	77.1
	4	21	3.3	3.3	80.4
	5	10	1.6	1.6	82.0
	6	13	2.1	2.1	84.0
	7	10	1.6	1.6	85.6
	8	39	6.2	6.2	91.8
	9	20	3.2	3.2	94.9
	10	14	2.2	2.2	97.2
	11	11	1.7	1.7	98.9
	12	4	.6	.6	99.5
	13	2	.3	.3	99.8
	14	1	.2	.2	100.0
	Total	633	100.0	100.0	

information_strain

		Frequency	Percent	Valid Percent	Cumulative Percent
Valid	4	122	19.3	19.3	19.3
	5	43	6.8	6.8	26.1
	6	33	5.2	5.2	31.3
	7	42	6.6	6.6	37.9
	8	65	10.3	10.3	48.2
	9	29	4.6	4.6	52.8
	10	33	5.2	5.2	58.0
	11	29	4.6	4.6	62.6
	12	27	4.3	4.3	66.8
	13	23	3.6	3.6	70.5
	14	16	2.5	2.5	73.0
	15	18	2.8	2.8	75.8
	16	57	9.0	9.0	84.8
	17	24	3.8	3.8	88.6
	18	22	3.5	3.5	92.1
	19	11	1.7	1.7	93.8
	20	11	1.7	1.7	95.6
	21	8	1.3	1.3	96.8
	22	7	1.1	1.1	97.9
	23	6	.9	.9	98.9
	24	3	.5	.5	99.4

information_strain

		Frequency	Percent	Valid Percent	Cumulative Percent
	25	1	.2	.2	99.5
	28	3	.5	.5	100.0
	Total	633	100.0	100.0	

information_overload

		Frequency	Percent	Valid Percent	Cumulative Percent
Valid	4	32	5.1	5.1	5.1
	5	12	1.9	1.9	7.0
	6	8	1.3	1.3	8.2
	7	8	1.3	1.3	9.5
	8	19	3.0	3.0	12.5
	9	14	2.2	2.2	14.7
	10	23	3.6	3.6	18.3
	11	20	3.2	3.2	21.5
	12	21	3.3	3.3	24.8
	13	24	3.8	3.8	28.6
	14	24	3.8	3.8	32.4
	15	31	4.9	4.9	37.3
	16	76	12.0	12.0	49.3
	17	61	9.6	9.6	58.9
	18	36	5.7	5.7	64.6
	19	42	6.6	6.6	71.2
	20	30	4.7	4.7	76.0
	21	29	4.6	4.6	80.6
	22	28	4.4	4.4	85.0
	23	17	2.7	2.7	87.7
	24	14	2.2	2.2	89.9
	25	22	3.5	3.5	93.4
	26	7	1.1	1.1	94.5
	27	5	.8	.8	95.3
	28	30	4.7	4.7	100.0
	Total	633	100.0	100.0	

brs_total

		Frequency	Percent	Valid Percent	Cumulative Percent
Valid	1	5	.8	.8	.8
	1	4	.6	.6	1.4
	1	3	.5	.5	1.9
	2	7	1.1	1.1	3.0
	2	10	1.6	1.6	4.6
	2	13	2.1	2.1	6.6
	2	20	3.2	3.2	9.8
	2	16	2.5	2.5	12.3
	2	23	3.6	3.6	16.0
	3	24	3.8	3.8	19.7
	3	41	6.5	6.5	26.2
	3	53	8.4	8.4	34.6
	3	81	12.8	12.8	47.4
	3	45	7.1	7.1	54.5
	3	47	7.4	7.4	61.9
	4	48	7.6	7.6	69.5
	4	37	5.8	5.8	75.4
	4	36	5.7	5.7	81.0
	4	34	5.4	5.4	86.4
	4	16	2.5	2.5	88.9
	4	15	2.4	2.4	91.3
	5	12	1.9	1.9	93.2
	5	9	1.4	1.4	94.6
	5	9	1.4	1.4	96.1
	5	25	3.9	3.9	100.0
	Total	633	100.0	100.0	

pss_total

		Frequency	Percent	Valid Percent	Cumulative Percent
Valid	0	1	.2	.2	.2
	2	1	.2	.2	.3
	3	7	1.1	1.1	1.4
	4	7	1.1	1.1	2.5
	5	7	1.1	1.1	3.6
	6	7	1.1	1.1	4.7
	7	5	.8	.8	5.5
	8	9	1.4	1.4	7.0
	9	13	2.1	2.1	9.0
	10	14	2.2	2.2	11.2
	11	25	3.9	3.9	15.2
	12	14	2.2	2.2	17.4
	13	21	3.3	3.3	20.7
	14	23	3.6	3.6	24.3
	15	21	3.3	3.3	27.6
	16	33	5.2	5.2	32.9
	17	29	4.6	4.6	37.4
	18	30	4.7	4.7	42.2
	19	34	5.4	5.4	47.6
	20	58	9.2	9.2	56.7
	21	43	6.8	6.8	63.5
	22	44	7.0	7.0	70.5
	23	23	3.6	3.6	74.1
	24	27	4.3	4.3	78.4
	25	28	4.4	4.4	82.8
	26	13	2.1	2.1	84.8
	27	15	2.4	2.4	87.2
	28	14	2.2	2.2	89.4
	29	14	2.2	2.2	91.6
	30	9	1.4	1.4	93.0
	31	6	.9	.9	94.0
	32	7	1.1	1.1	95.1
	33	9	1.4	1.4	96.5
	34	3	.5	.5	97.0
	35	7	1.1	1.1	98.1
	36	5	.8	.8	98.9
	37	2	.3	.3	99.2
	38	2	.3	.3	99.5

pss_total

	Frequency	Percent	Valid Percent	Cumulative Percent
39	2	.3	.3	99.8
40	1	.2	.2	100.0
Total	633	100.0	100.0	

cerq_blamingothers

		Frequency	Percent	Valid Percent	Cumulative Percent
Valid	4	54	8.5	8.6	8.6
	5	43	6.8	6.8	15.4
	6	44	7.0	7.0	22.3
	7	48	7.6	7.6	30.0
	8	86	13.6	13.6	43.6
	9	57	9.0	9.0	52.6
	10	40	6.3	6.3	59.0
	11	54	8.5	8.6	67.5
	12	68	10.7	10.8	78.3
	13	28	4.4	4.4	82.7
	14	40	6.3	6.3	89.1
	15	24	3.8	3.8	92.9
	16	21	3.3	3.3	96.2
	17	7	1.1	1.1	97.3
	18	8	1.3	1.3	98.6
	19	3	.5	.5	99.0
	20	6	.9	1.0	100.0
	Total	631	99.7	100.0	
Missing	System	2	.3		
Total		633	100.0		

cerq_catastrophizing

		Frequency	Percent	Valid Percent	Cumulative Percent
Valid	4	36	5.7	5.7	5.7
	5	24	3.8	3.8	9.5
	6	43	6.8	6.8	16.3
	7	54	8.5	8.5	24.8
	8	65	10.3	10.3	35.1
	9	52	8.2	8.2	43.3
	10	44	7.0	7.0	50.2
	11	61	9.6	9.6	59.9
	12	71	11.2	11.2	71.1
	13	37	5.8	5.8	76.9
	14	40	6.3	6.3	83.3
	15	35	5.5	5.5	88.8
	16	32	5.1	5.1	93.8
	17	16	2.5	2.5	96.4
	18	8	1.3	1.3	97.6
	19	7	1.1	1.1	98.7
	20	8	1.3	1.3	100.0
	Total	633	100.0	100.0	

cerq_puttingintoperspective

		Frequency	Percent	Valid Percent	Cumulative Percent
Valid	4	6	.9	.9	.9
	5	6	.9	.9	1.9
	6	5	.8	.8	2.7
	7	12	1.9	1.9	4.6
	8	21	3.3	3.3	7.9
	9	19	3.0	3.0	10.9
	10	34	5.4	5.4	16.3
	11	28	4.4	4.4	20.7
	12	62	9.8	9.8	30.5
	13	60	9.5	9.5	40.0
	14	55	8.7	8.7	48.7
	15	74	11.7	11.7	60.3
	16	74	11.7	11.7	72.0
	17	30	4.7	4.7	76.8
	18	50	7.9	7.9	84.7
	19	38	6.0	6.0	90.7
	20	59	9.3	9.3	100.0
	Total	633	100.0	100.0	

cerq_positivereappraisal

		Frequency	Percent	Valid Percent	Cumulative Percent
Valid	4	3	.5	.5	.5
	5	4	.6	.6	1.1
	6	3	.5	.5	1.6
	7	9	1.4	1.4	3.0
	8	9	1.4	1.4	4.4
	9	15	2.4	2.4	6.8
	10	14	2.2	2.2	9.0
	11	29	4.6	4.6	13.6
	12	47	7.4	7.4	21.0
	13	47	7.4	7.4	28.4
	14	54	8.5	8.5	37.0
	15	61	9.6	9.6	46.6
	16	81	12.8	12.8	59.4
	17	52	8.2	8.2	67.6
	18	57	9.0	9.0	76.6
	19	45	7.1	7.1	83.7
	20	103	16.3	16.3	100.0
	Total	633	100.0	100.0	

cerq_refocusonplanning

		Frequency	Percent	Valid Percent	Cumulative Percent
Valid	4	3	.5	.5	.5
	6	3	.5	.5	.9
	7	3	.5	.5	1.4
	8	9	1.4	1.4	2.8
	9	8	1.3	1.3	4.1
	10	15	2.4	2.4	6.5
	11	21	3.3	3.3	9.8
	12	38	6.0	6.0	15.8
	13	47	7.4	7.4	23.2
	14	45	7.1	7.1	30.3
	15	56	8.8	8.8	39.2
	16	97	15.3	15.3	54.5
	17	68	10.7	10.7	65.2
	18	72	11.4	11.4	76.6
	19	53	8.4	8.4	85.0
	20	95	15.0	15.0	100.0
	Total	633	100.0	100.0	

cerq_positiverefocusing

		Frequency	Percent	Valid Percent	Cumulative Percent
Valid	4	12	1.9	1.9	1.9
	5	13	2.1	2.1	3.9
	6	26	4.1	4.1	8.1
	7	15	2.4	2.4	10.4
	8	32	5.1	5.1	15.5
	9	44	7.0	7.0	22.4
	10	51	8.1	8.1	30.5
	11	45	7.1	7.1	37.6
	12	66	10.4	10.4	48.0
	13	58	9.2	9.2	57.2
	14	64	10.1	10.1	67.3
	15	54	8.5	8.5	75.8
	16	57	9.0	9.0	84.8
	17	27	4.3	4.3	89.1
	18	24	3.8	3.8	92.9
	19	22	3.5	3.5	96.4
	20	23	3.6	3.6	100.0
	Total	633	100.0	100.0	

cerq_rumination

		Frequency	Percent	Valid Percent	Cumulative Percent
Valid	4	5	.8	.8	.8
	5	2	.3	.3	1.1
	6	4	.6	.6	1.7
	7	5	.8	.8	2.5
	8	10	1.6	1.6	4.1
	9	14	2.2	2.2	6.3
	10	24	3.8	3.8	10.1
	11	37	5.8	5.8	16.0
	12	54	8.5	8.5	24.5
	13	52	8.2	8.2	32.7
	14	61	9.6	9.6	42.3
	15	55	8.7	8.7	51.0
	16	67	10.6	10.6	61.6
	17	61	9.6	9.6	71.2
	18	51	8.1	8.1	79.3
	19	42	6.6	6.6	85.9
	20	89	14.1	14.1	100.0
	Total	633	100.0	100.0	

cerq_acceptance

		Frequency	Percent	Valid Percent	Cumulative Percent
Valid	4	3	.5	.5	.5
	5	1	.2	.2	.6
	6	4	.6	.6	1.3
	7	7	1.1	1.1	2.4
	8	18	2.8	2.8	5.2
	9	14	2.2	2.2	7.4
	10	23	3.6	3.6	11.1
	11	39	6.2	6.2	17.2
	12	68	10.7	10.7	28.0
	13	66	10.4	10.4	38.4
	14	74	11.7	11.7	50.1
	15	72	11.4	11.4	61.5
	16	57	9.0	9.0	70.5
	17	49	7.7	7.7	78.2
	18	43	6.8	6.8	85.0
	19	38	6.0	6.0	91.0
	20	57	9.0	9.0	100.0
	Total	633	100.0	100.0	

cerq_selfblame

		Frequency	Percent	Valid Percent	Cumulative Percent
Valid	4	7	1.1	1.1	1.1
	5	8	1.3	1.3	2.4
	6	15	2.4	2.4	4.7
	7	12	1.9	1.9	6.6
	8	34	5.4	5.4	12.0
	9	43	6.8	6.8	18.8
	10	58	9.2	9.2	28.0
	11	70	11.1	11.1	39.0
	12	67	10.6	10.6	49.6
	13	75	11.8	11.8	61.5
	14	53	8.4	8.4	69.8
	15	50	7.9	7.9	77.7
	16	49	7.7	7.7	85.5
	17	35	5.5	5.5	91.0
	18	22	3.5	3.5	94.5
	19	18	2.8	2.8	97.3
	20	17	2.7	2.7	100.0
	Total	633	100.0	100.0	

sontus_total

		Frequency	Percent	Valid Percent	Cumulative Percent
Valid	5	16	2.5	2.5	2.5
	6	25	3.9	3.9	6.5
	7	25	3.9	3.9	10.4
	8	31	4.9	4.9	15.3
	9	35	5.5	5.5	20.9
	10	53	8.4	8.4	29.2
	11	48	7.6	7.6	36.8
	12	53	8.4	8.4	45.2
	13	63	10.0	10.0	55.1
	14	62	9.8	9.8	64.9
	15	41	6.5	6.5	71.4
	16	46	7.3	7.3	78.7
	17	40	6.3	6.3	85.0
	18	42	6.6	6.6	91.6
	19	20	3.2	3.2	94.8
	20	16	2.5	2.5	97.3
	21	6	.9	.9	98.3
	22	4	.6	.6	98.9
	23	7	1.1	1.1	100.0
	Total	633	100.0	100.0	

ghq_total

		Frequency	Percent	Valid Percent	Cumulative Percent
Valid	0	7	1.1	1.1	1.1
	1	8	1.3	1.3	2.4
	2	7	1.1	1.1	3.5
	3	3	.5	.5	3.9
	4	13	2.1	2.1	6.0
	5	12	1.9	1.9	7.9
	6	19	3.0	3.0	10.9
	7	24	3.8	3.8	14.7
	8	27	4.3	4.3	19.0
	9	27	4.3	4.3	23.2
	10	28	4.4	4.4	27.6
	11	24	3.8	3.8	31.4
	12	36	5.7	5.7	37.1
	13	25	3.9	3.9	41.1
	14	26	4.1	4.1	45.2
	15	30	4.7	4.7	49.9
	16	33	5.2	5.2	55.1
	17	22	3.5	3.5	58.6
	18	29	4.6	4.6	63.2
	19	21	3.3	3.3	66.5
	20	21	3.3	3.3	69.8
	21	22	3.5	3.5	73.3
	22	25	3.9	3.9	77.3
	23	14	2.2	2.2	79.5
	24	22	3.5	3.5	82.9
	25	18	2.8	2.8	85.8
	26	16	2.5	2.5	88.3
	27	10	1.6	1.6	89.9
	28	8	1.3	1.3	91.2
	29	11	1.7	1.7	92.9
	30	14	2.2	2.2	95.1
	31	9	1.4	1.4	96.5
	32	4	.6	.6	97.2
	33	5	.8	.8	97.9
	34	3	.5	.5	98.4
	35	4	.6	.6	99.1
	36	6	.9	.9	100.0
	Total	633	100.0	100.0	

Histogram

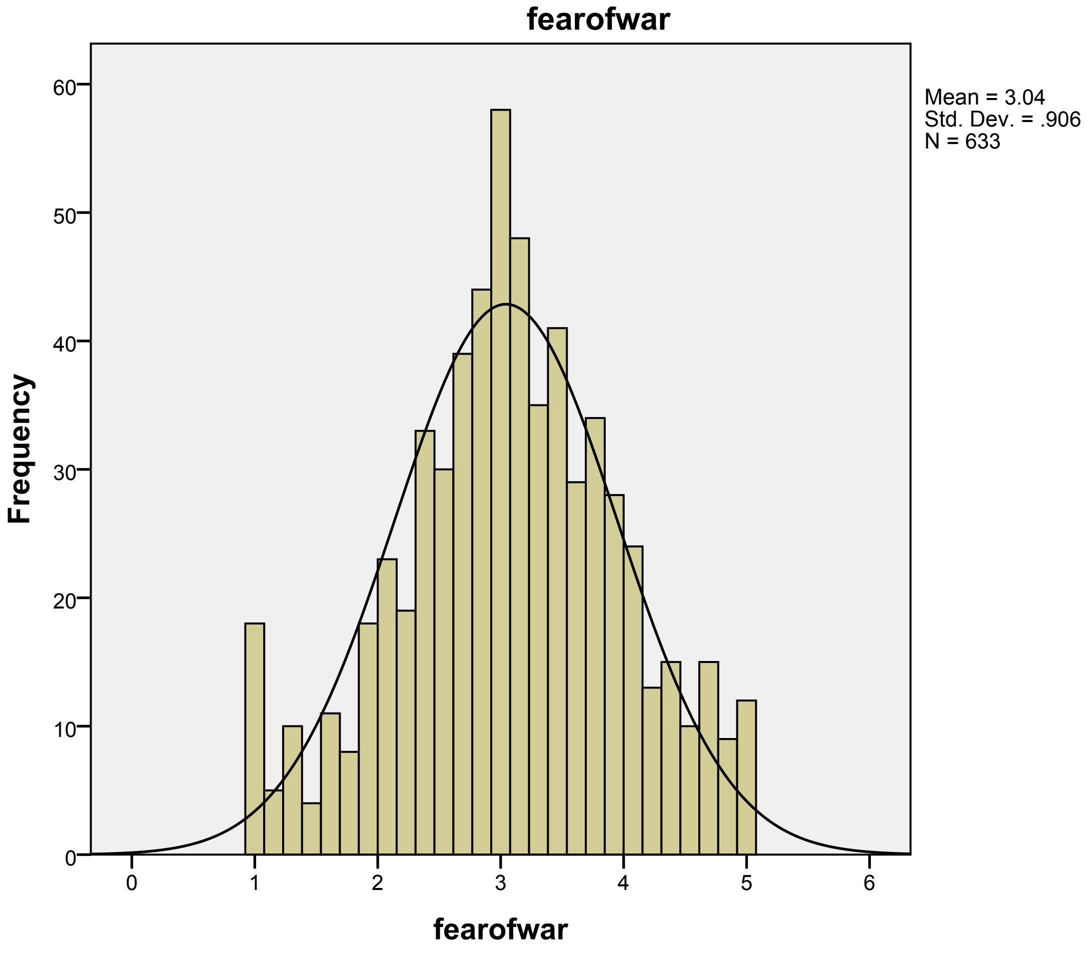

fearofwar
Mean = 3.04
Std. Dev. = .906
N = 633
Frequency
0
10
20
30
40
50
60
0
1
2
3
4
5
6
fearofwar

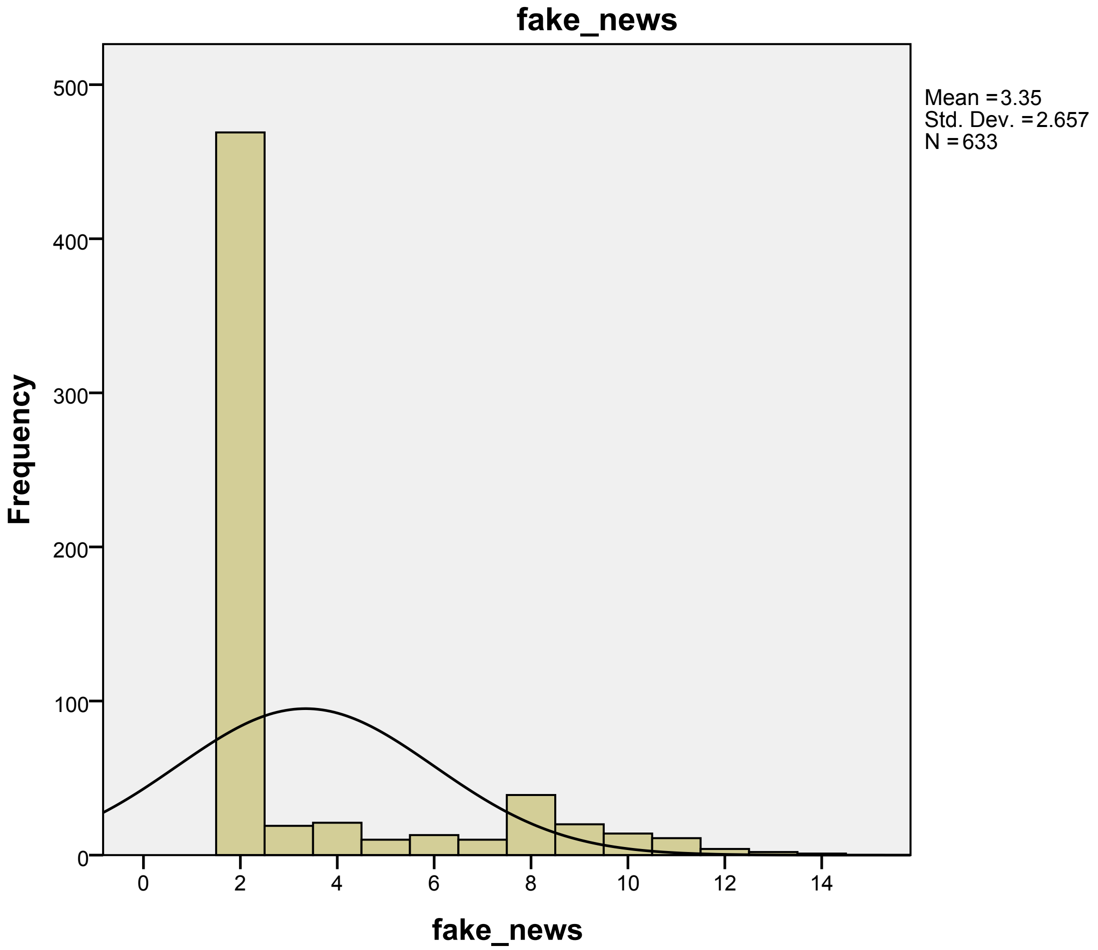
fake_news
Mean = 3.35
Std. Dev. = 2.657
N = 633
Frequency
500
400
300
200
100
0
0
2
4
6
8
10
12
14
fake_news

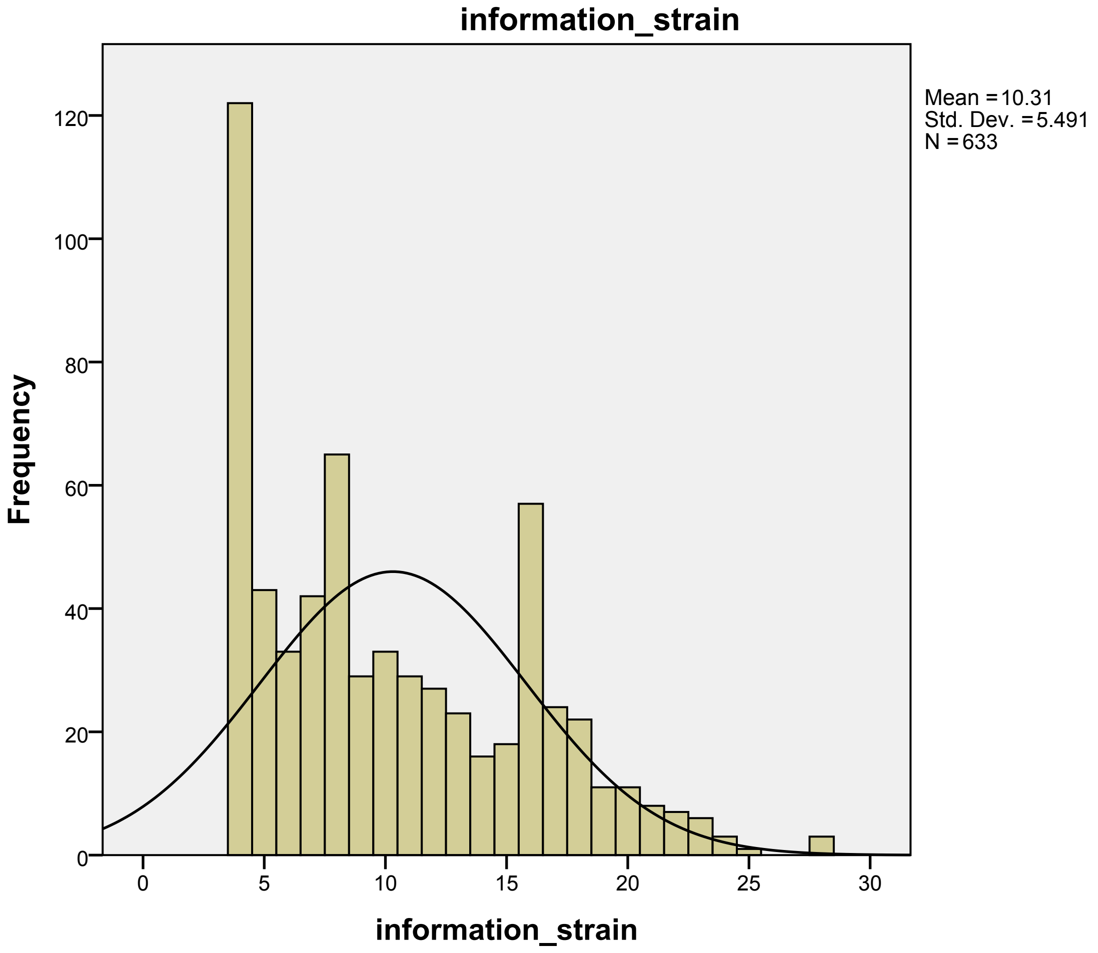
information_strain
Mean = 10.31
Std. Dev. = 5.491
N = 633
Frequency
0
20
40
60
80
100
120
0
5
10
15
20
25
30
information_strain

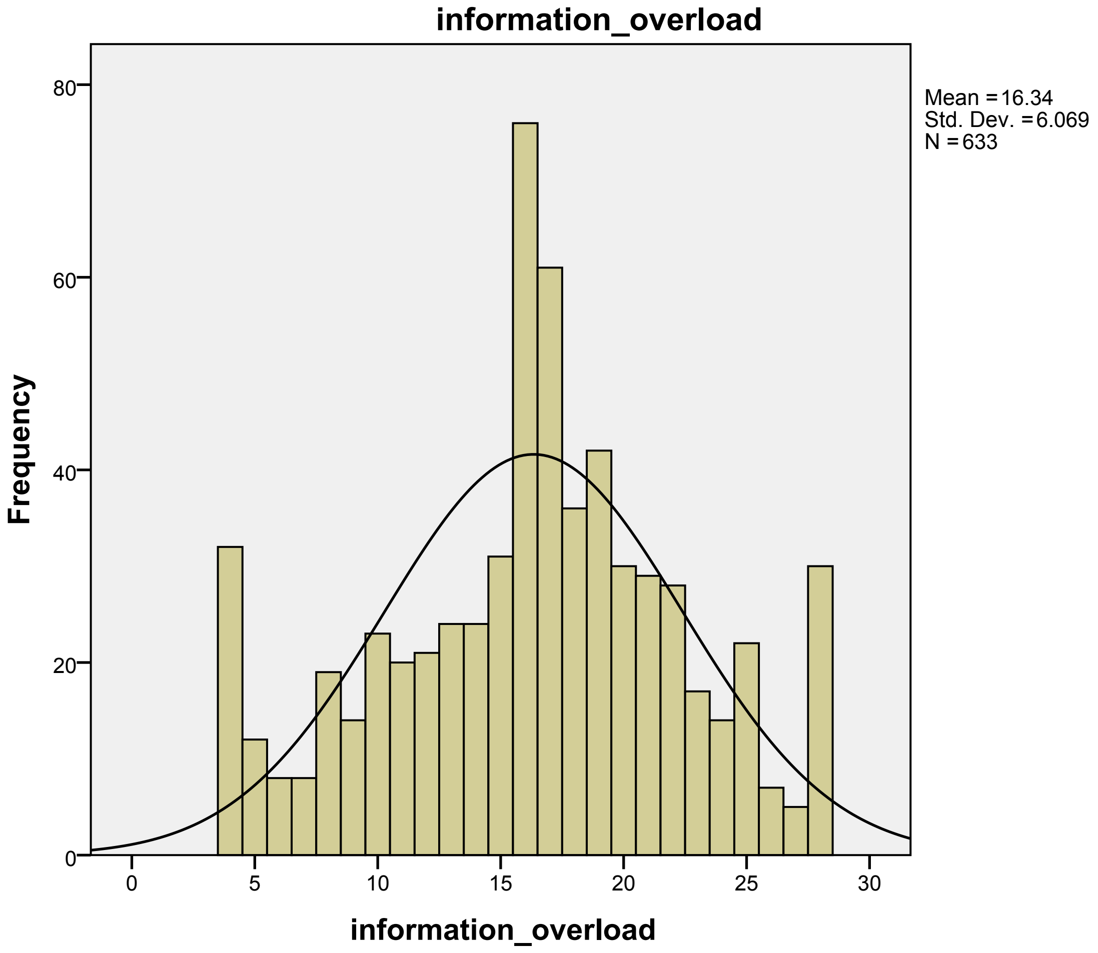
information_overload
Frequency
80
60
40
20
0
0
5
10
15
20
25
30
information_overload
Mean = 16.34
Std. Dev. = 6.069
N = 633

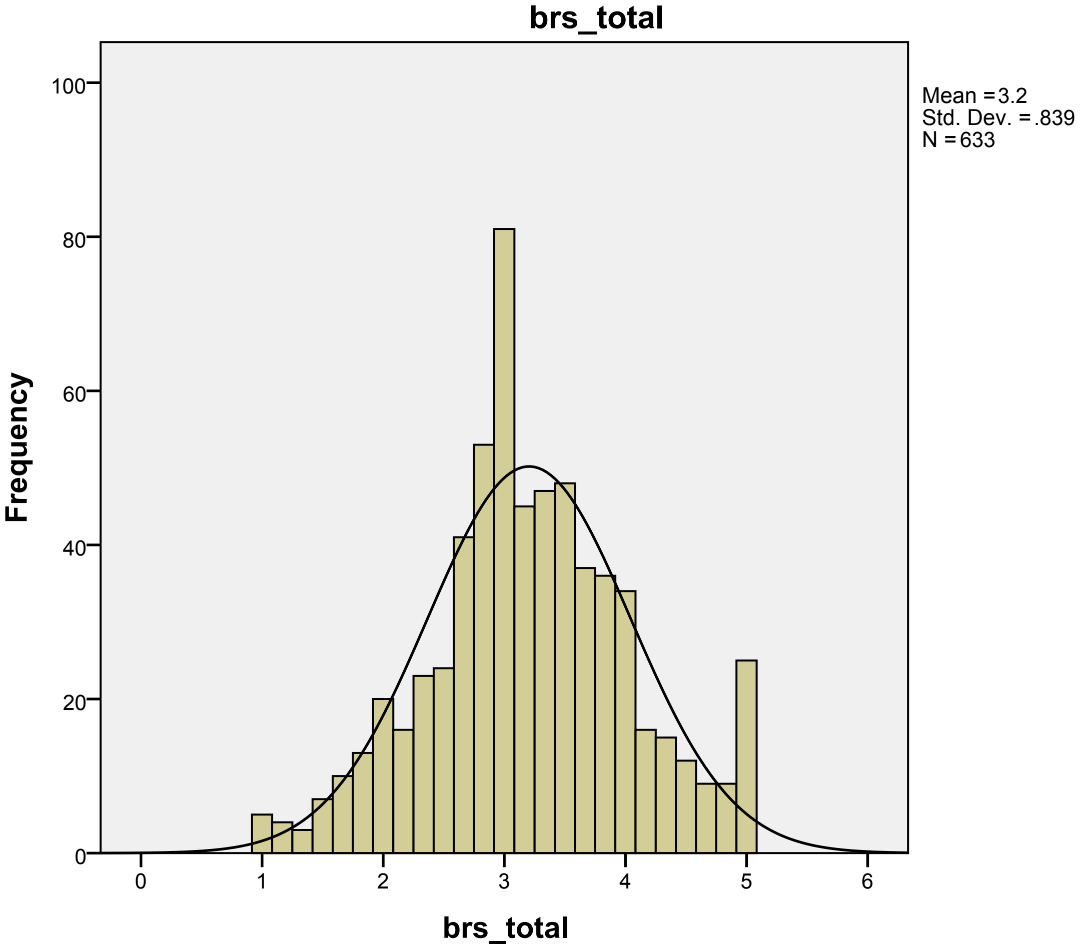
brs_total
Mean =3.2
Std. Dev. =.839
N =633
Frequency
100
80
60
40
20
0
0
1
2
3
4
5
6
brs_total

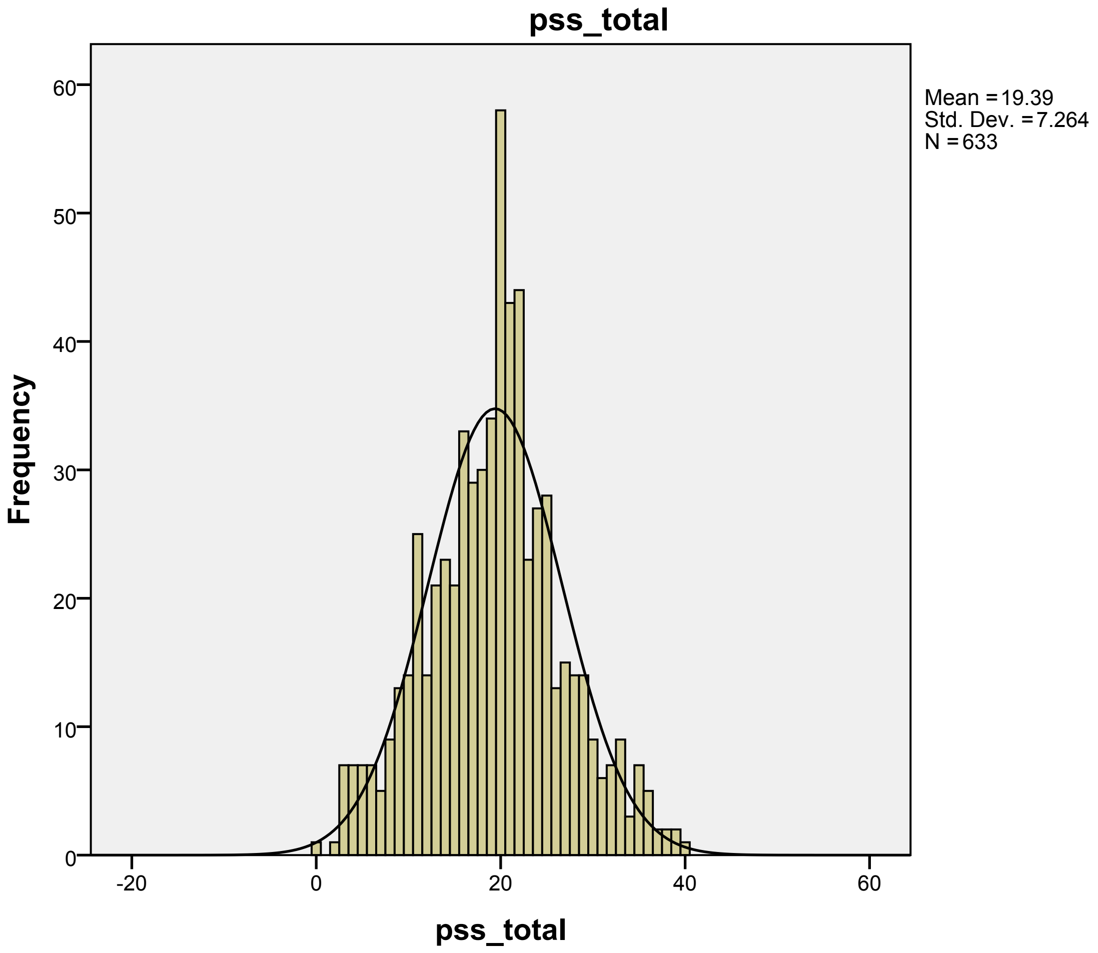
pss_total
Mean = 19.39
Std. Dev. = 7.264
N = 633
Frequency
0
10
20
30
40
50
60
-20
0
20
40
60
pss_total

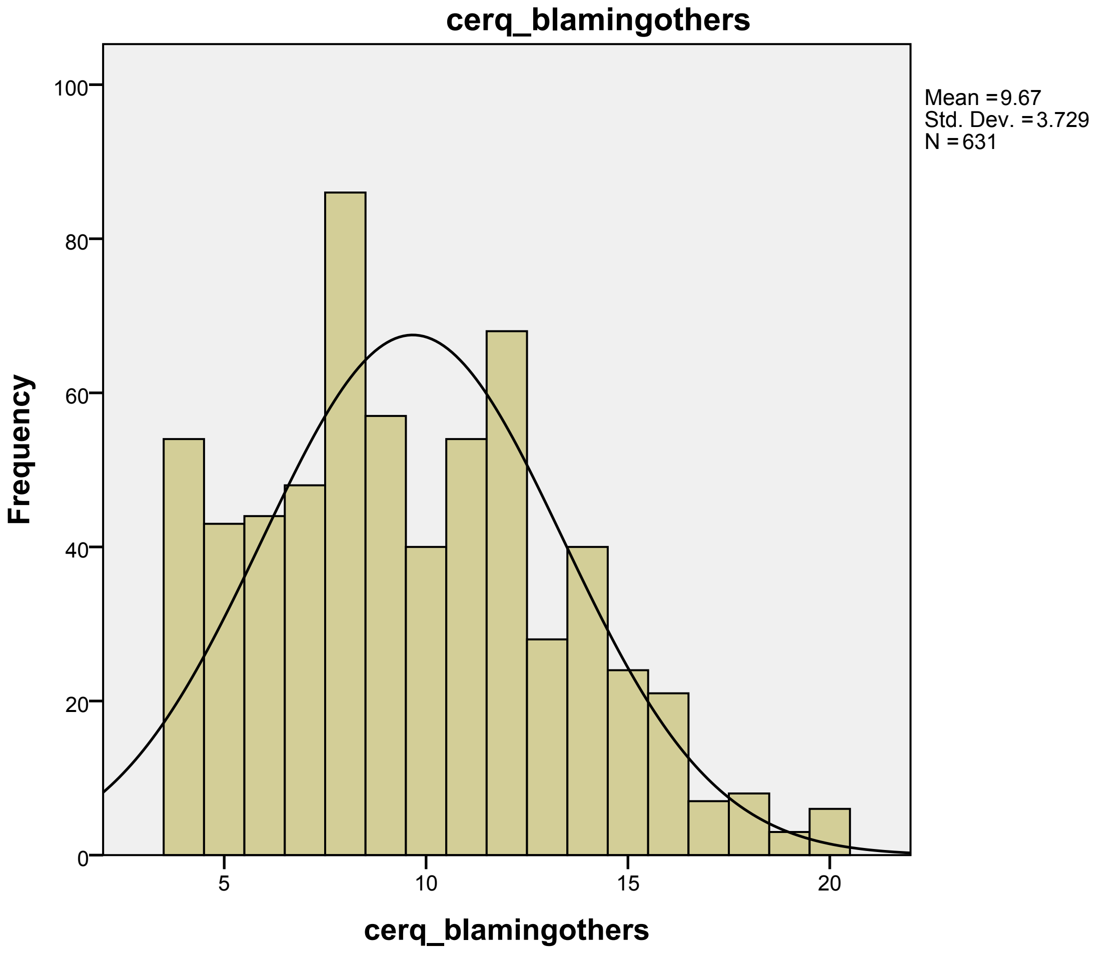
cerq_blamingothers
Mean = 9.67
Std. Dev. = 3.729
N = 631
Frequency
0
20
40
60
80
100
5
10
15
20
cerq_blamingothers

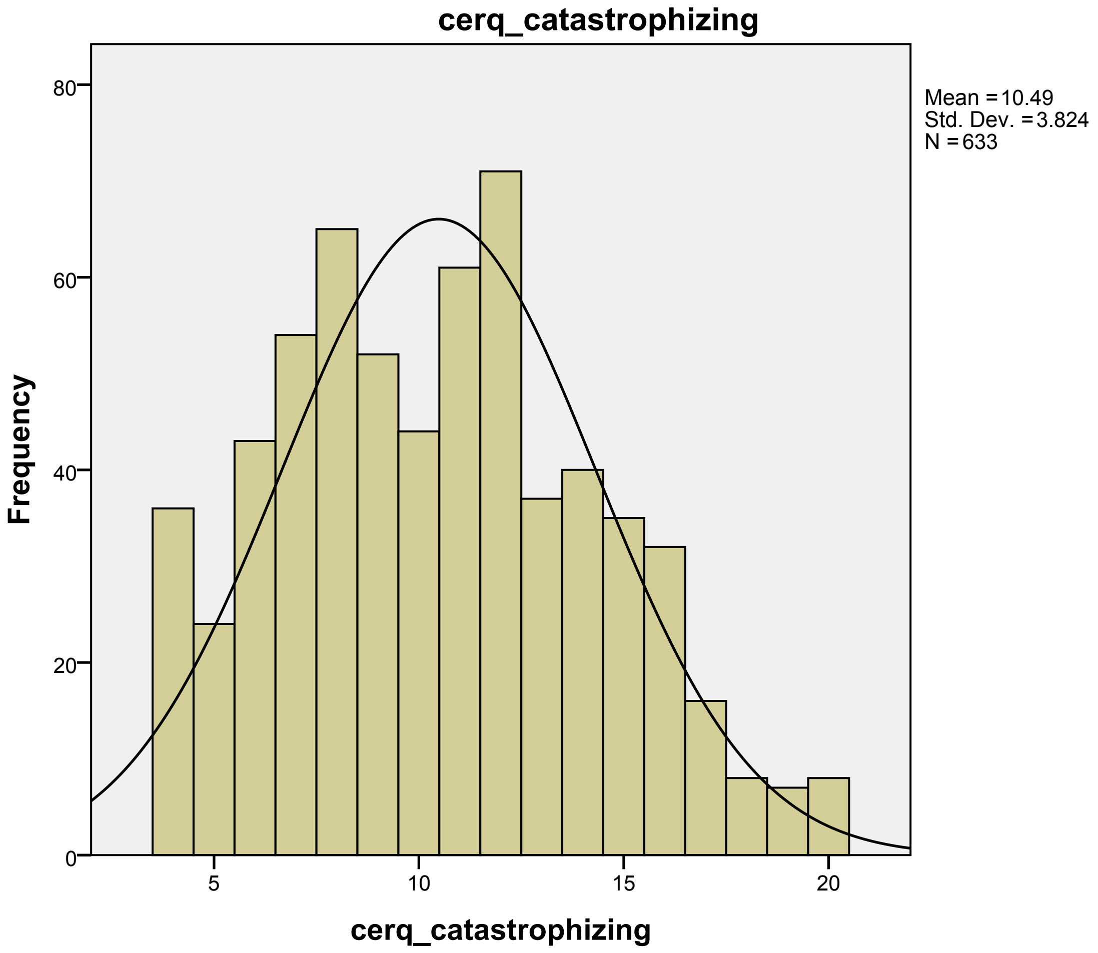
cerq_catastrophizing
Mean = 10.49
Std. Dev. = 3.824
N = 633
Frequency
0
20
40
60
80
5
10
15
20
cerq_catastrophizing

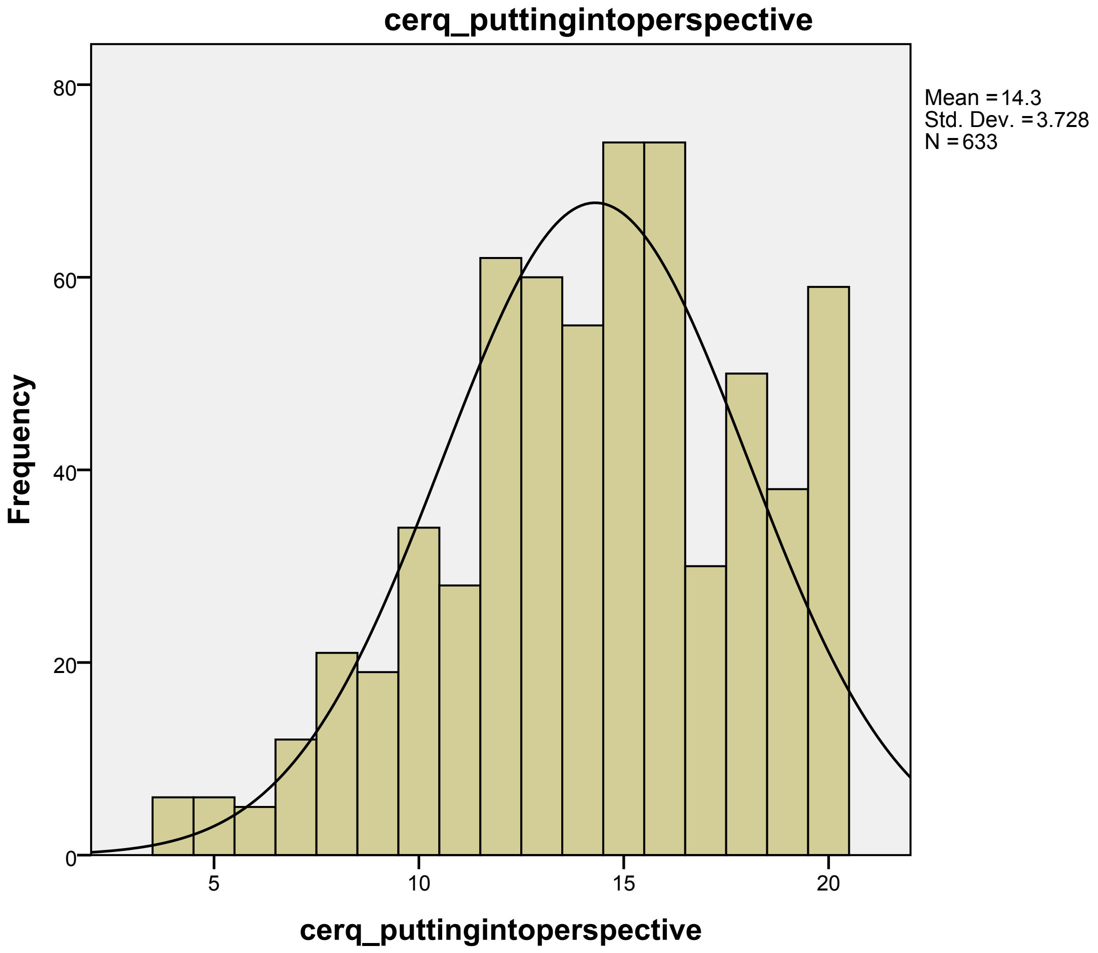
cerq_puttingintoperspective
Mean = 14.3
Std. Dev. = 3.728
N = 633
Frequency
80
60
40
20
0
5
10
15
20
cerq_puttingintoperspective

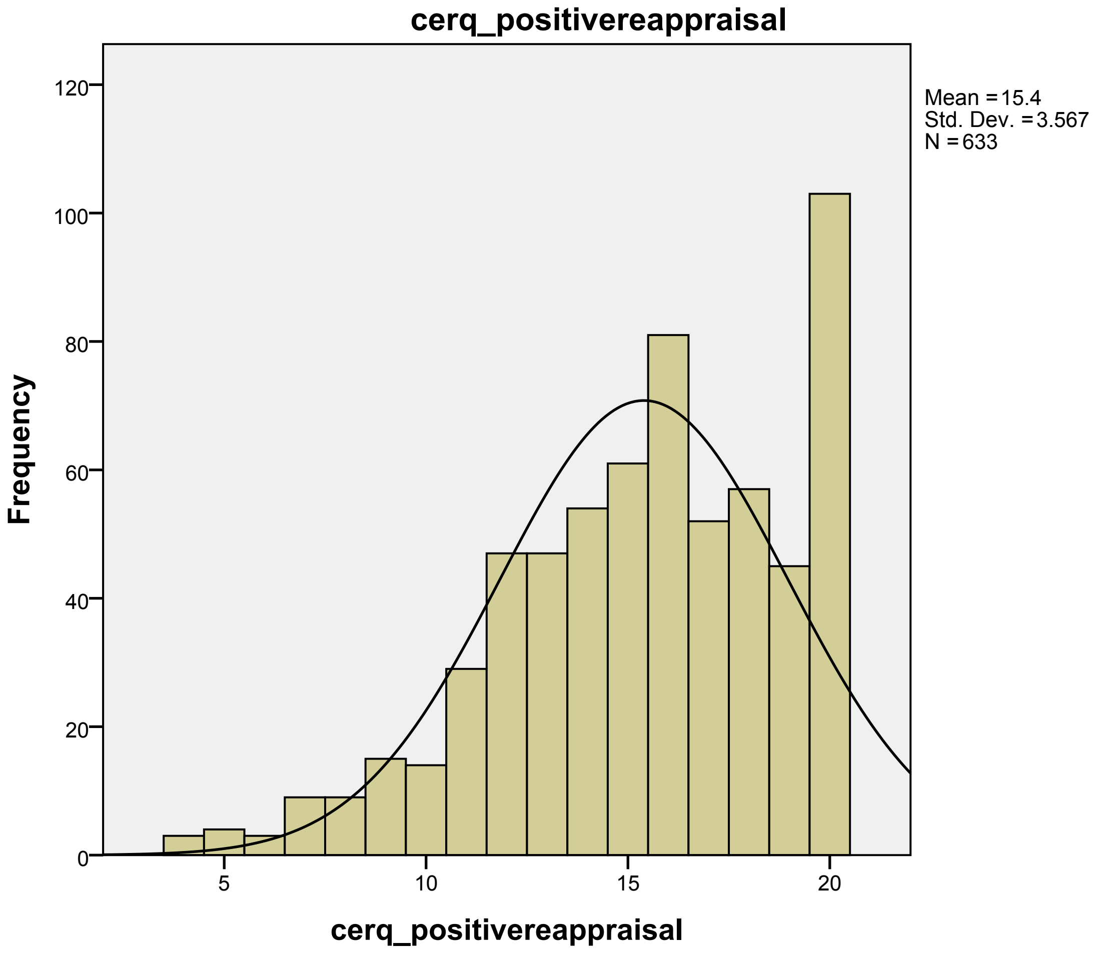
cerq_positivereappraisal
Mean = 15.4
Std. Dev. = 3.567
N = 633
Frequency
0
20
40
60
80
100
120
5
10
15
20
cerq_positivereappraisal

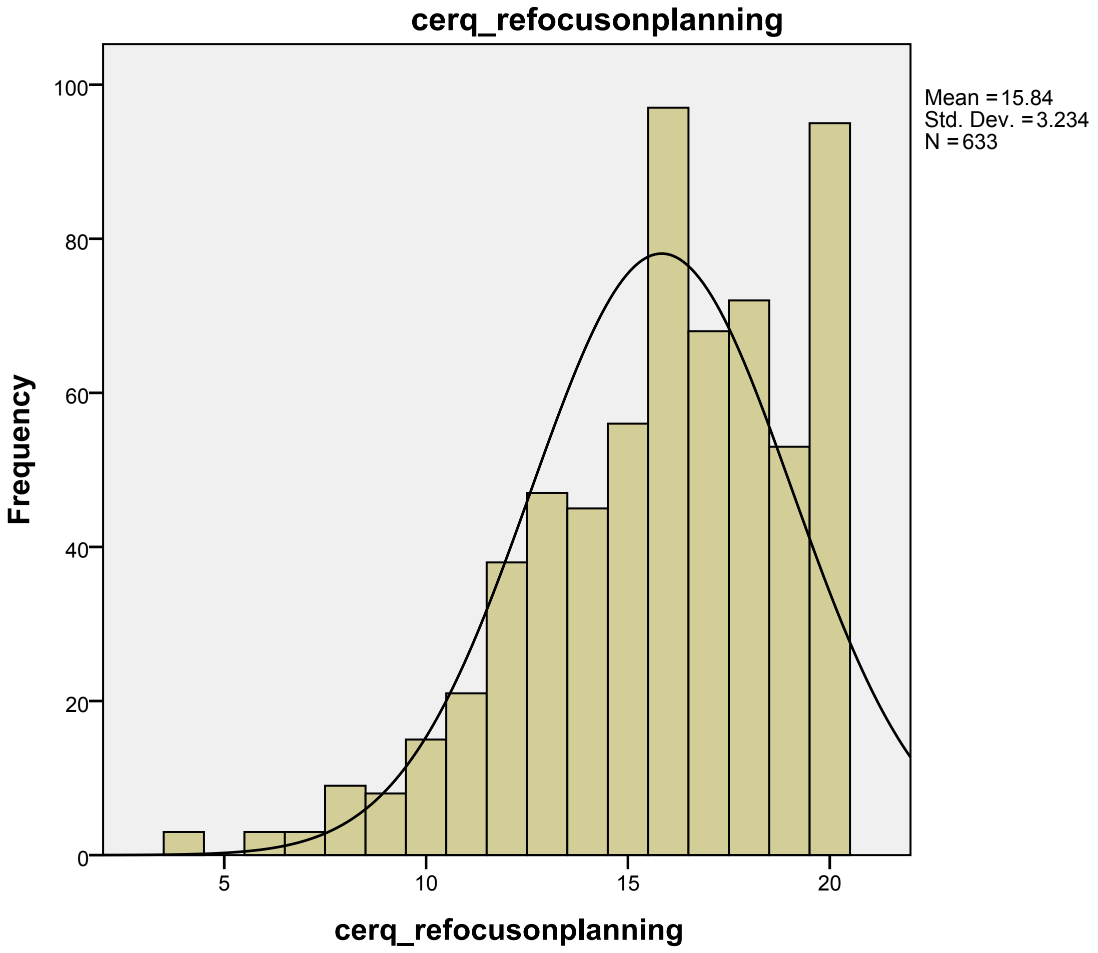
cerq_refocusonplanning
Frequency
cerq_refocusonplanning
0
20
40
60
80
100
5
10
15
20
Mean = 15.84
Std. Dev. = 3.234
N = 633

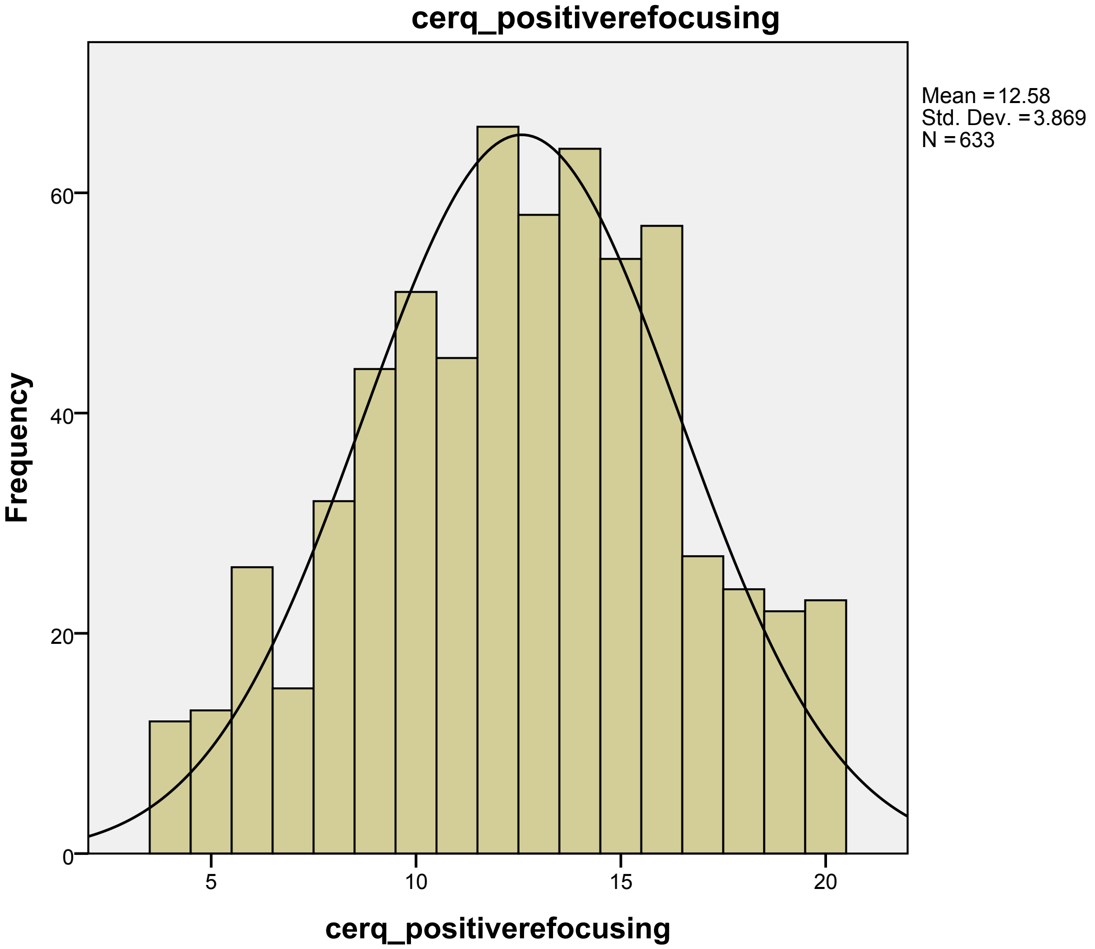
cerq_positiverefocusing
Mean = 12.58
Std. Dev. = 3.869
N = 633
Frequency
0
20
40
60
5
10
15
20
cerq_positiverefocusing

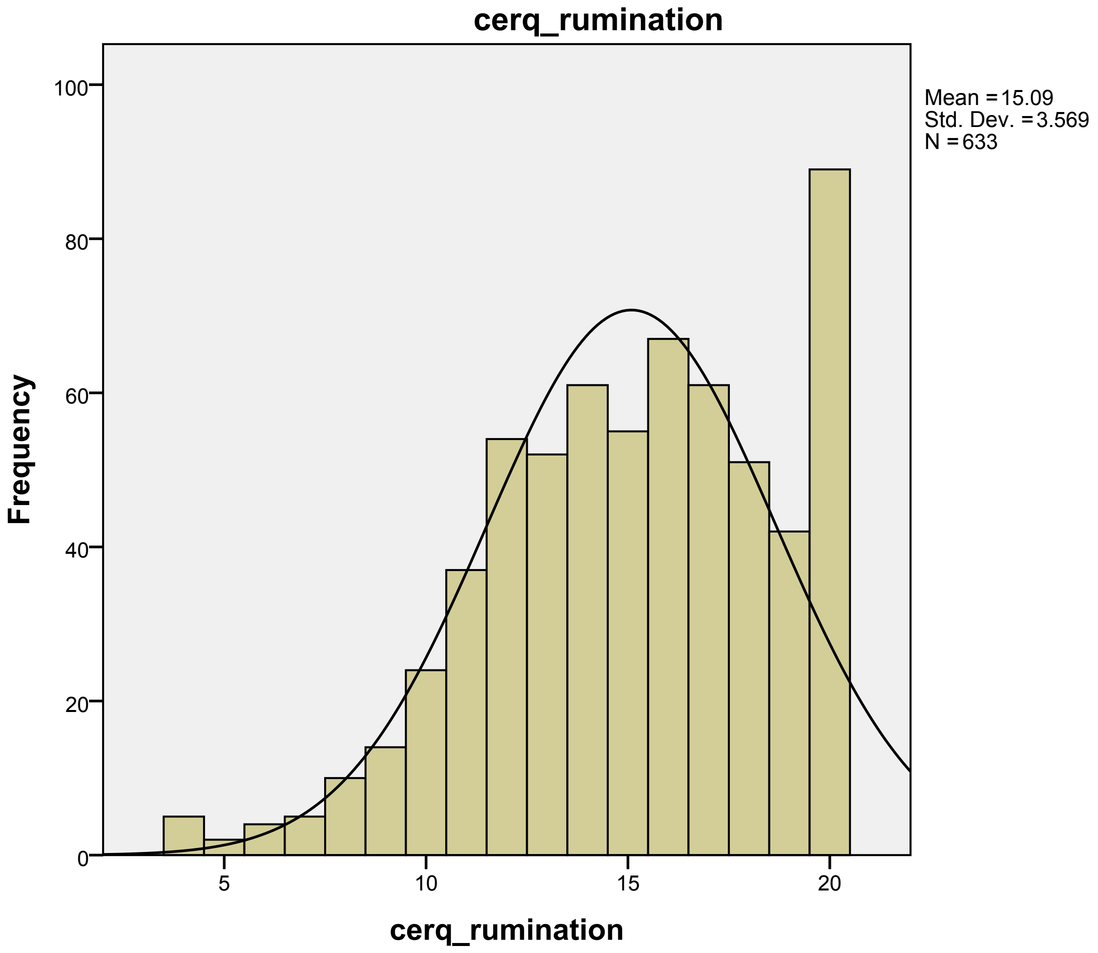
cerq_rumination
Mean = 15.09
Std. Dev. = 3.569
N = 633
Frequency
100
80
60
40
20
0
5
10
15
20
cerq_rumination

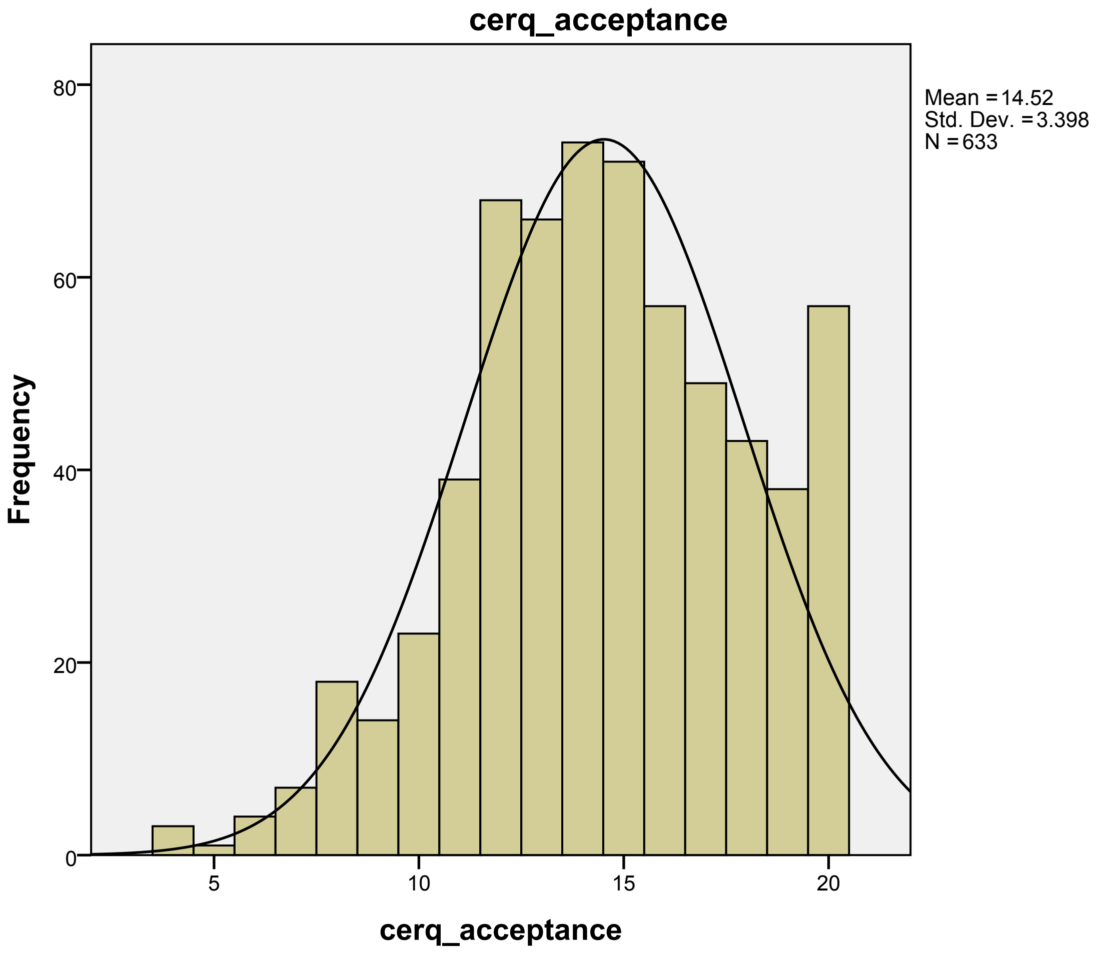
cerq_acceptance
Mean = 14.52
Std. Dev. = 3.398
N = 633
Frequency
80
60
40
20
0
5
10
15
20
cerq_acceptance

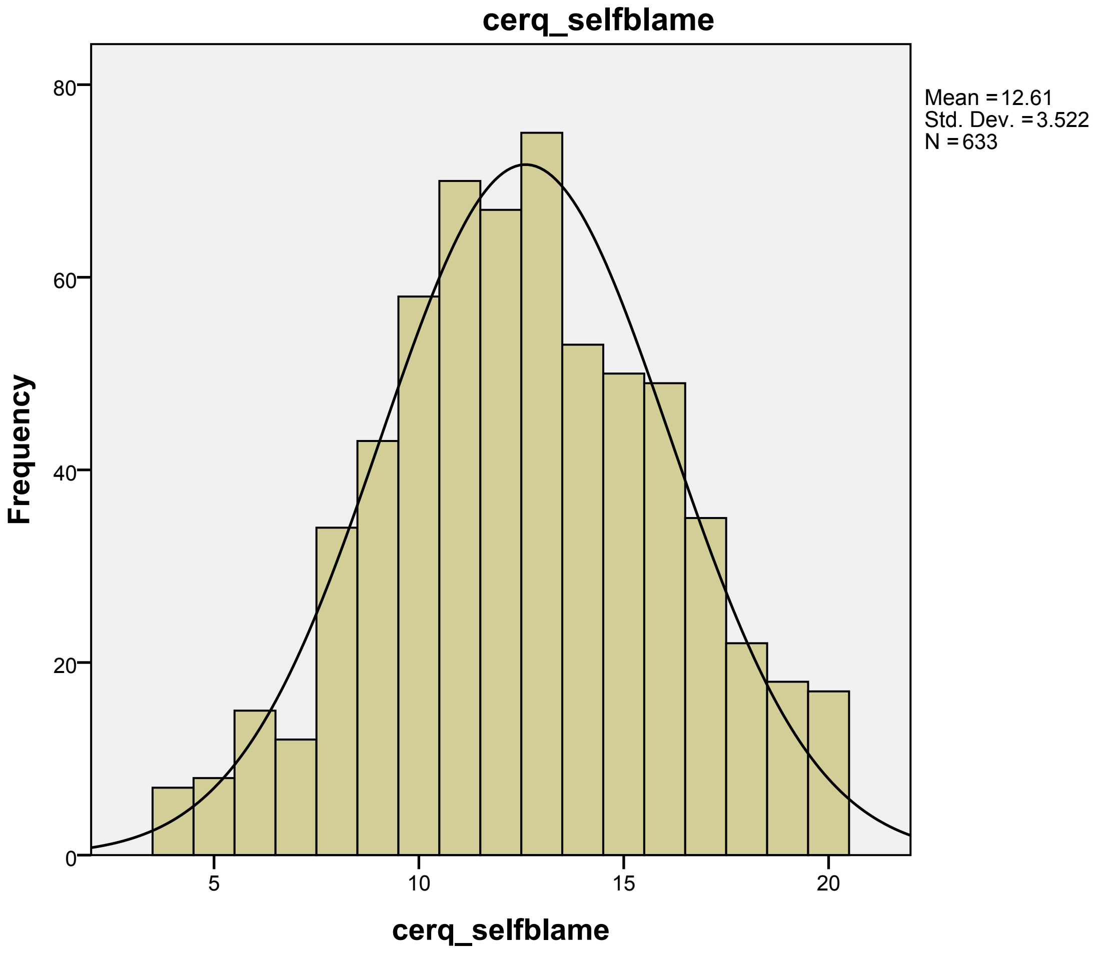
cerq_selfblame
Mean = 12.61
Std. Dev. = 3.522
N = 633
Frequency
80
60
40
20
0
5
10
15
20
cerq_selfblame

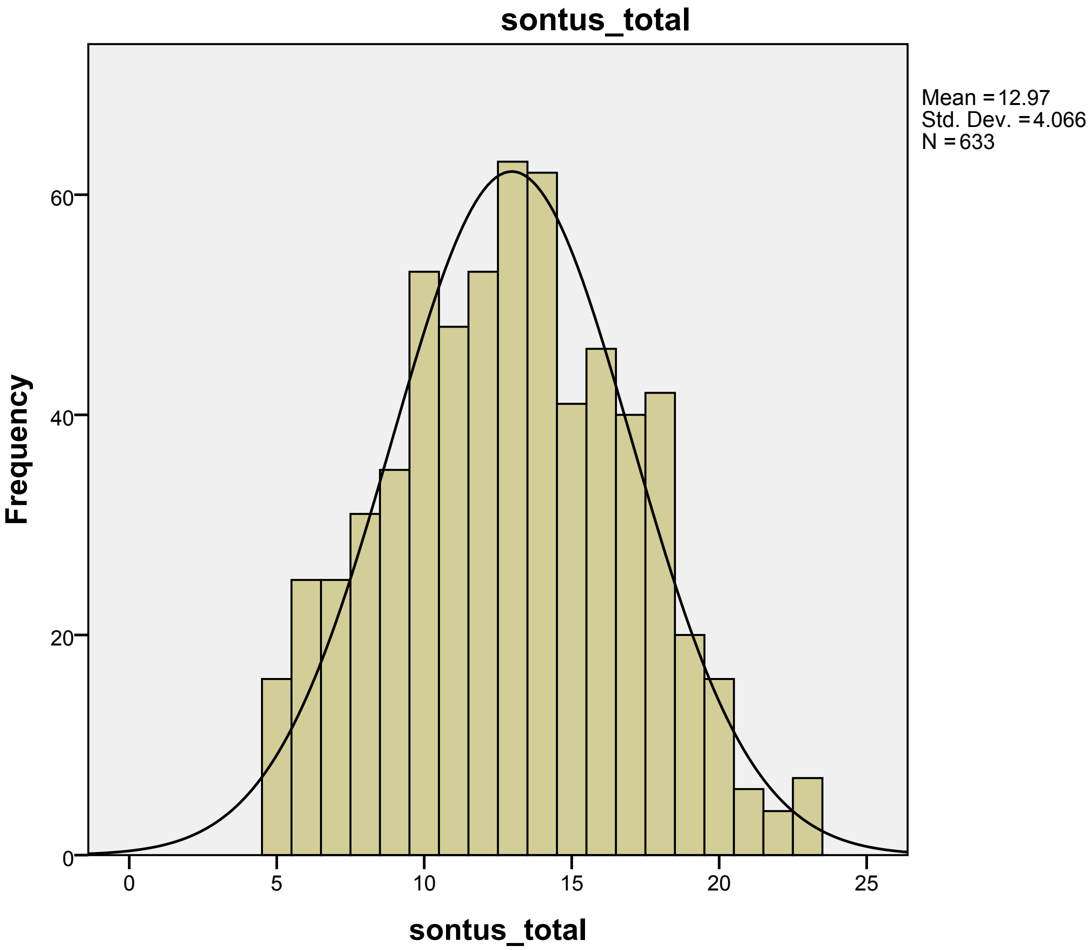
sontus_total
Mean = 12.97
Std. Dev. = 4.066
N = 633
Frequency
0
20
40
60
0
5
10
15
20
25
sontus_total

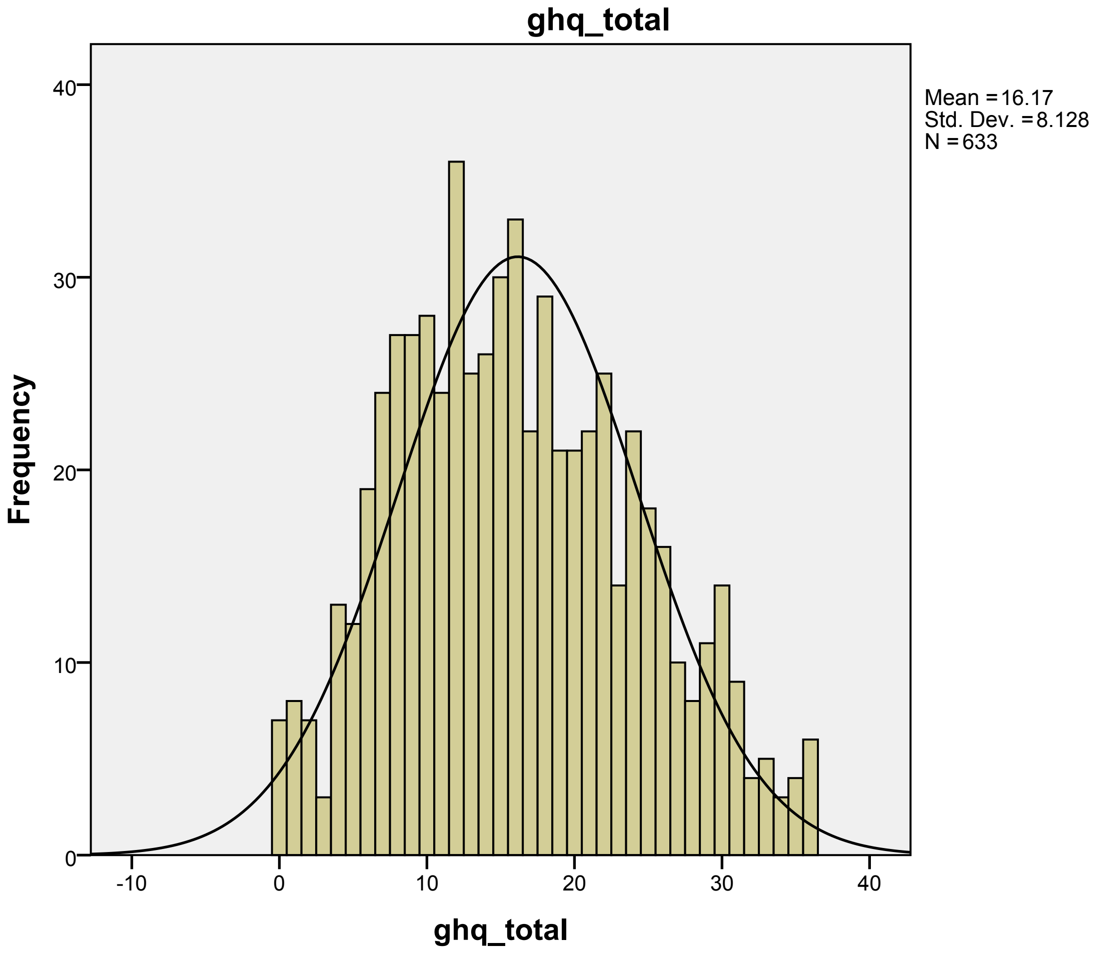
ghq_total
Mean = 16.17
Std. Dev. = 8.128
N = 633
Frequency
40
30
20
10
0
-10
0
10
20
30
40
ghq_total

```
GET
  FILE='D:\Psiho-An 6\Conferinta razboi\FearOfWar_database_1.sav'.
DATASET NAME DataSet1 WINDOW=FRONT.
GRAPH
  /SCATTERPLOT(BIVAR)=information_overload WITH fake_news
  /MISSING=LISTWISE.
```

Graph

Notes

Output Created		16-APR-2023 18:23:43
Comments		
Input	Data	D:\Psiho-An 6\Conferinta razboi\FearOfWar_database_1.sav
	Active Dataset	DataSet1
	Filter	<none>
	Weight	<none>
	Split File	<none>
	N of Rows in Working Data File	633
Syntax		GRAPH /SCATTERPLOT(BIVAR) =information_overload WITH fake_news /MISSING=LISTWISE.
Resources	Processor Time	00:00:01.00
	Elapsed Time	00:00:00.63

```
[DataSet1] D:\Psiho-An 6\Conferinta razboi\FearOfWar_database_1.sav
```

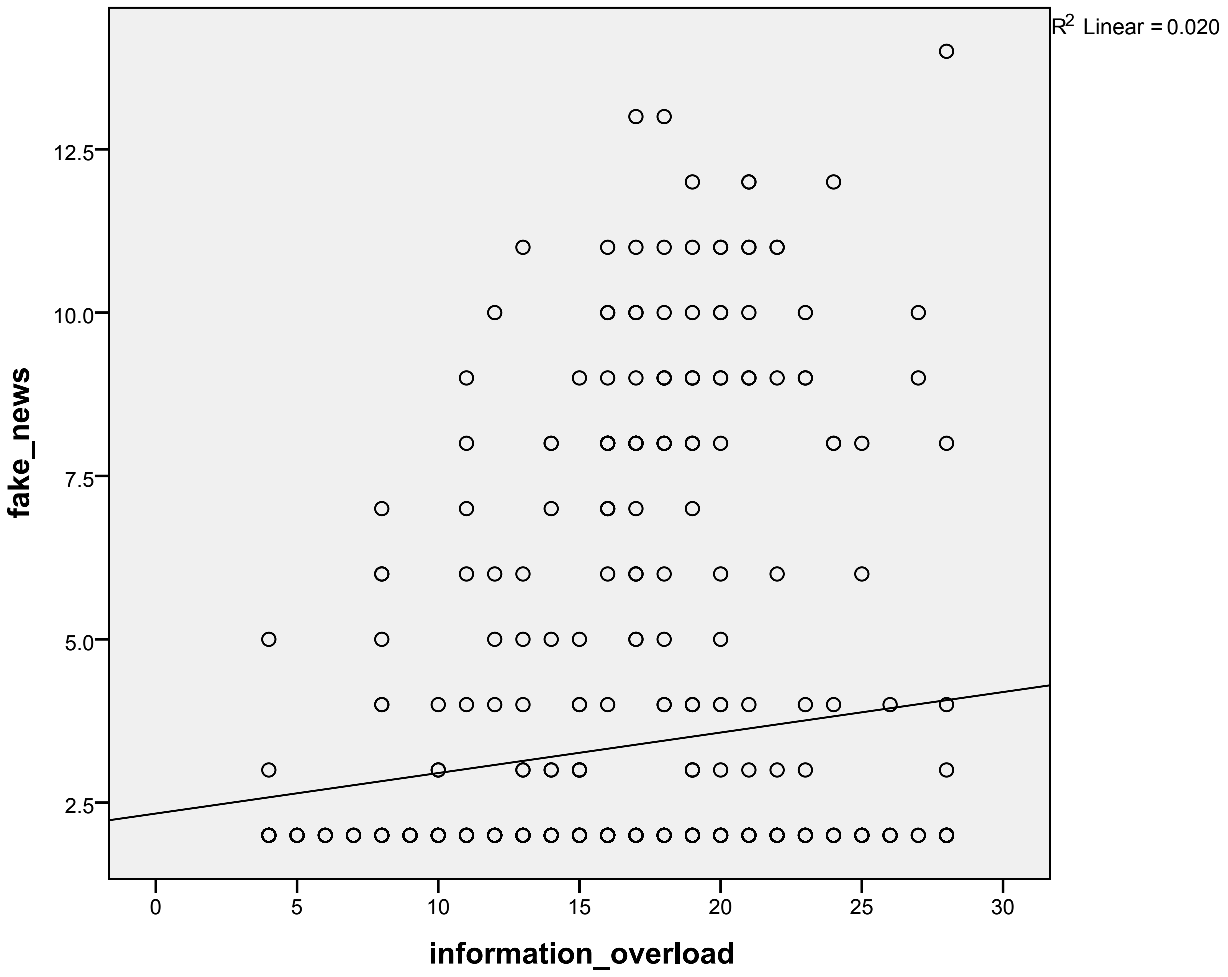


```
GRAPH
  /SCATTERPLOT(BIVAR)=information_strain WITH fake_news
  /MISSING=LISTWISE.
```

Graph

Notes

Output Created		16-APR-2023 18:24:40
Comments		
Input	Data	D:\Psiho-An 6\Conferinta razboi\FearOfWar_database_1.sav
	Active Dataset	DataSet1
	Filter	<none>
	Weight	<none>
	Split File	<none>
	N of Rows in Working Data File	633
Syntax		GRAPH /SCATTERPLOT(BIVAR) =information_strain WITH fake_news /MISSING=LISTWISE.
Resources	Processor Time	00:00:00.16
	Elapsed Time	00:00:00.16

```
[DataSet1] D:\Psiho-An 6\Conferinta razboi\FearOfWar_database_1.sav
```

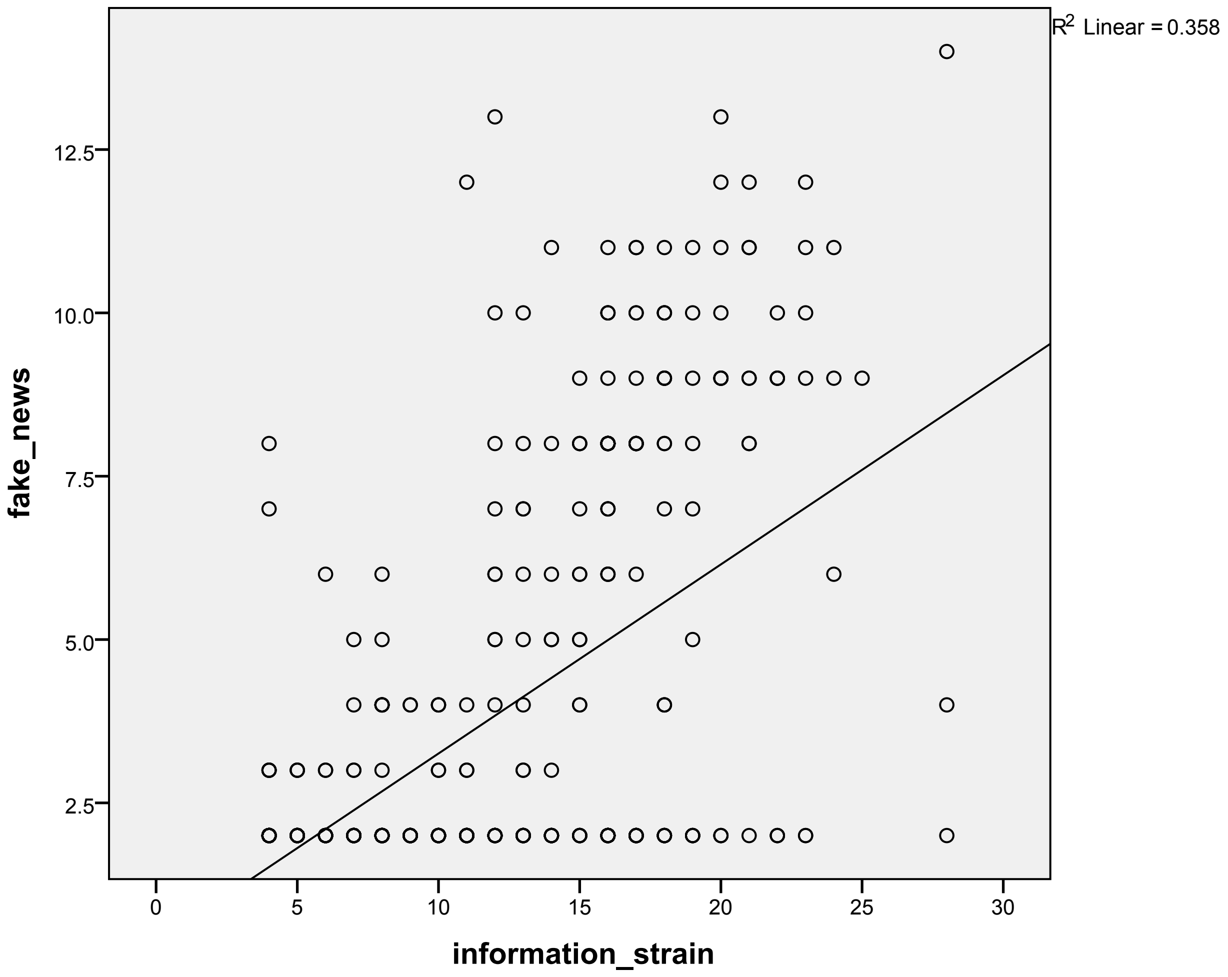


```
GRAPH
 /SCATTERPLOT(BIVAR)=sontus_total WITH fake_news
 /MISSING=LISTWISE.
```

Graph

Notes

Output Created		16-APR-2023 18:25:11
Comments		
Input	Data	D:\Psiho-An 6\Conferinta razboi\FearOfWar_database_1.sav
	Active Dataset	DataSet1
	Filter	<none>
	Weight	<none>
	Split File	<none>
	N of Rows in Working Data File	633
Syntax		GRAPH /SCATTERPLOT(BIVAR) =sontus_total WITH fake_news /MISSING=LISTWISE.
Resources	Processor Time	00:00:00.17
	Elapsed Time	00:00:00.14

```
[DataSet1] D:\Psiho-An 6\Conferinta razboi\FearOfWar_database_1.sav
```

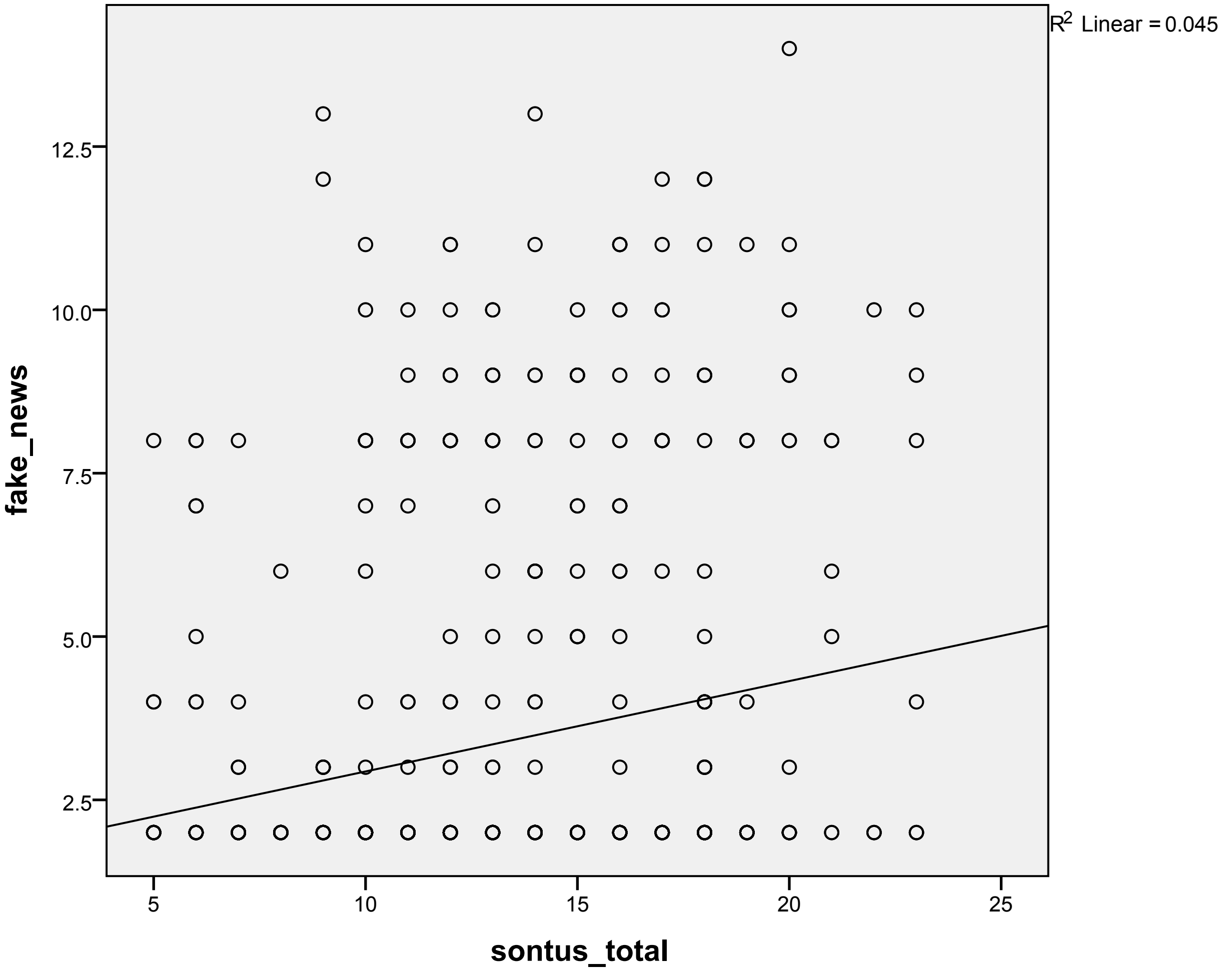


```
GRAPH
  /SCATTERPLOT(BIVAR)=information_overload WITH information_strain
  /MISSING=LISTWISE.
```

Graph

Notes

Output Created		16-APR-2023 18:27:22
Comments		
Input	Data	D:\Psiho-An 6\Conferinta razboi\FearOfWar_database_1.sav
	Active Dataset	DataSet1
	Filter	<none>
	Weight	<none>
	Split File	<none>
	N of Rows in Working Data File	633
Syntax		GRAPH /SCATTERPLOT(BIVAR) =information_overload WITH information_strain /MISSING=LISTWISE.
Resources	Processor Time	00:00:00.17
	Elapsed Time	00:00:00.16

```
[DataSet1] D:\Psiho-An 6\Conferinta razboi\FearOfWar_database_1.sav
```

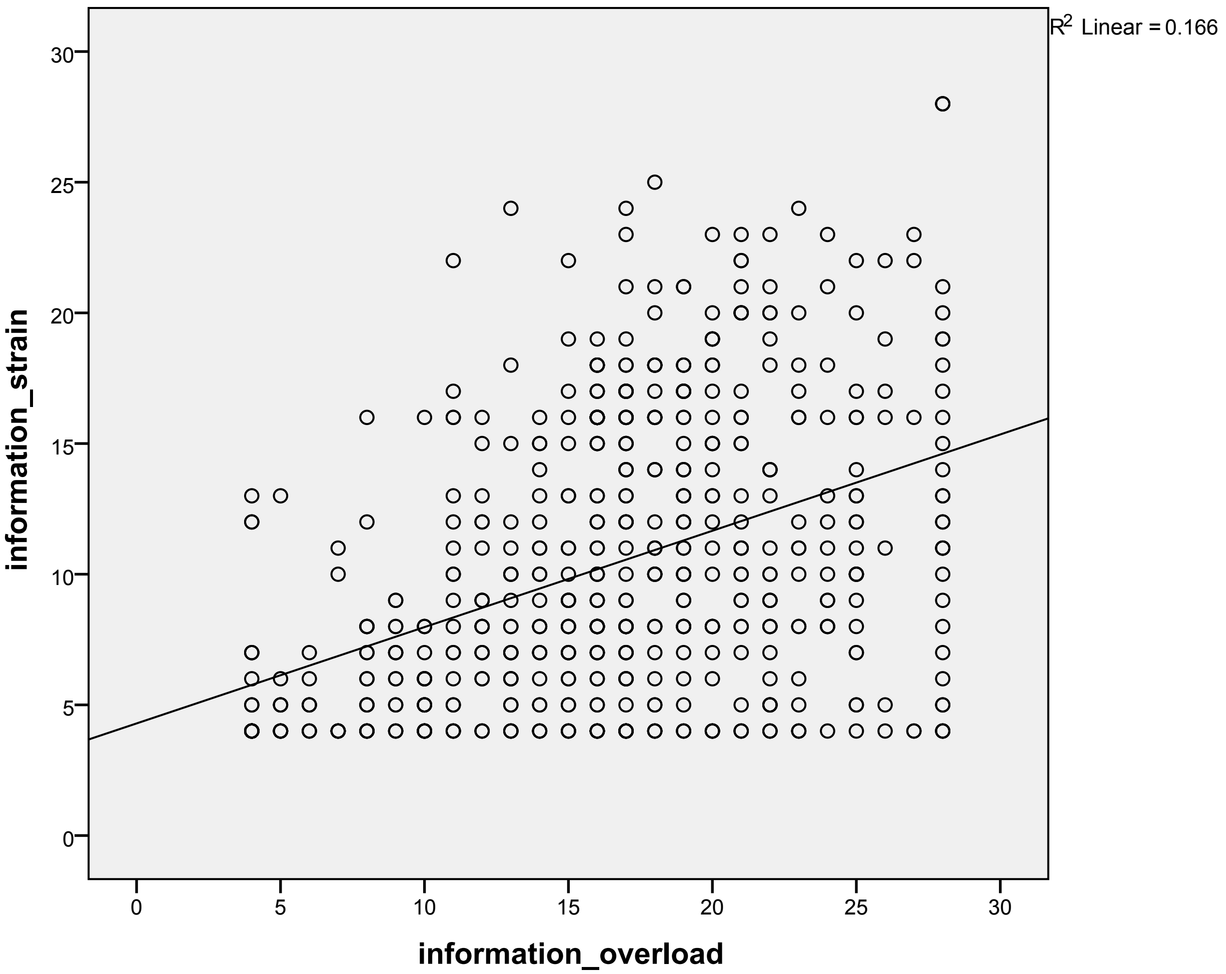


```
GRAPH
  /SCATTERPLOT(BIVAR)=sontus_total WITH information_strain
  /MISSING=LISTWISE.
```

Graph

Notes

Output Created		16-APR-2023 18:28:28
Comments		
Input	Data	D:\Psiho-An 6\Conferinta razboi\FearOfWar_database_1.sav
	Active Dataset	DataSet1
	Filter	<none>
	Weight	<none>
	Split File	<none>
	N of Rows in Working Data File	633
Syntax		GRAPH /SCATTERPLOT(BIVAR)=sontus_total WITH information_strain /MISSING=LISTWISE.
Resources	Processor Time	00:00:00.16
	Elapsed Time	00:00:00.13

```
[DataSet1] D:\Psiho-An 6\Conferinta razboi\FearOfWar_database_1.sav
```

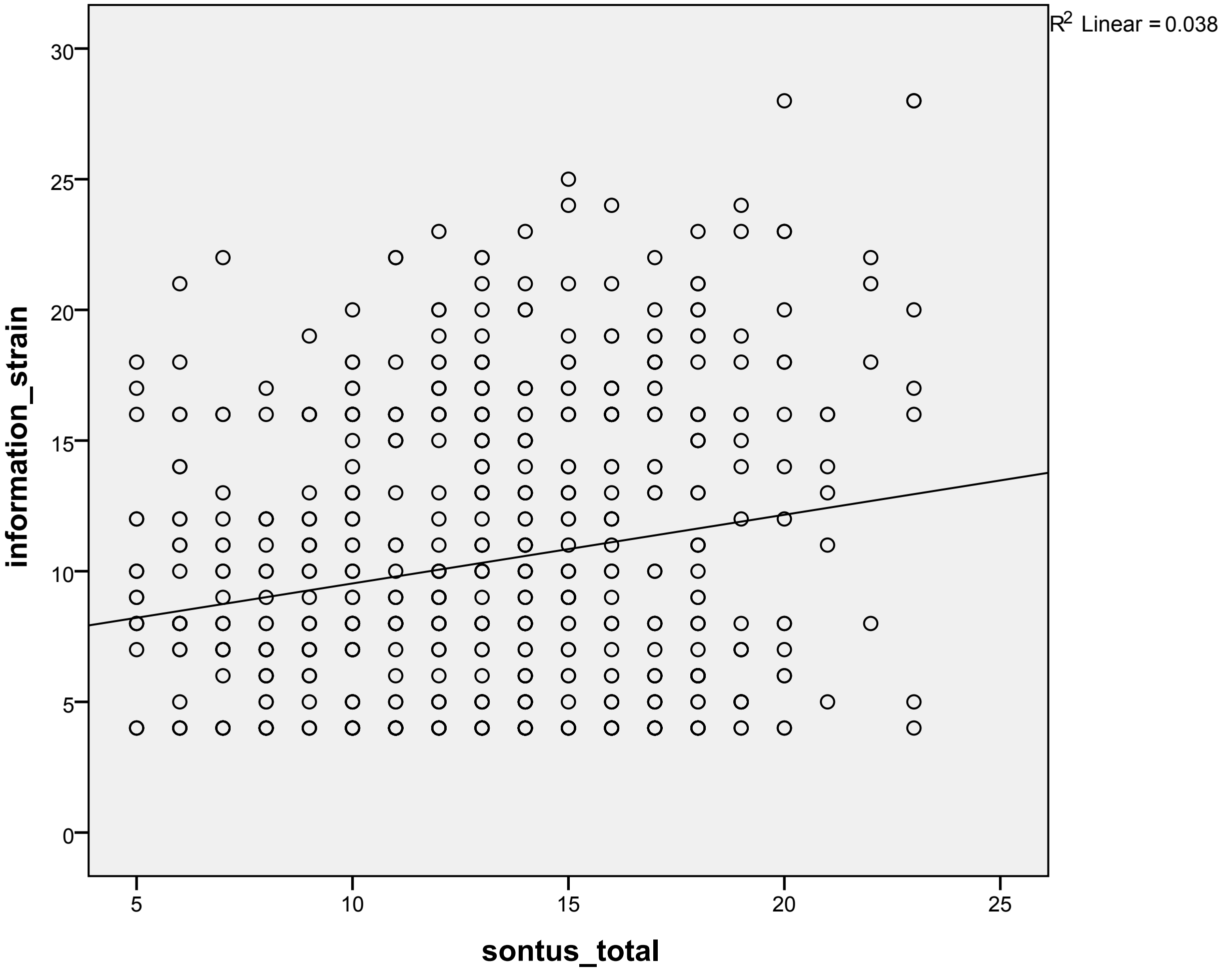
R² Linear = 0.038
information_strain
30
25
20
15
10
5
0
5
10
15
20
25
sontus_total

Results

Independent Samples T-Test

Independent Samples T-Test

							95% Confidence Interval				95% Confidence Interval	
		Statistic	df	p	Mean difference	SE difference	Lower	Upper		Effect Size	Lower	Upper
cerq_rumination	Student's t	1.72	631	0.043	0.524	0.3047	0.0216	Inf	Cohen's d	0.147	-0.0215	0.315
fearofwar	Student's t	2.34	631	0.010	0.181	0.0772	0.0537	Inf	Cohen's d	0.200	0.0314	0.369

Note. H_a Da > Nu

Independent Samples T-Test

Independent Samples T-Test

							95% Confidence Interval				95% Confidence Interval	
		Statistic	df	p	Mean difference	SE difference	Lower	Upper		Effect Size	Lower	Upper
cerq_blamingothers	Student's t	-1.66	629	0.049	-0.528	0.319	-Inf	-0.00278	Cohen's d	-0.142	-0.310	0.0268

Note. H_a Da < Nu

Correlation Matrix

Correlation Matrix

		information_strain	information_overload	fake_news	sontus_total
information_strain	Pearson's r	—			
	p-value	—			
	95% CI Upper	—			
	95% CI Lower	—			
	N	—			
information_overload	Pearson's r	0.407 ***	—		
	p-value	< .001	—		
	95% CI Upper	0.470	—		
	95% CI Lower	0.340	—		
	N	633	—		
fake_news	Pearson's r	0.598 ***	0.142 ***	—	
	p-value	< .001	< .001	—	
	95% CI Upper	0.646	0.217	—	
	95% CI Lower	0.546	0.064	—	
	N	633	633	—	
sontus_total	Pearson's r	0.195 ***	0.165 ***	0.212 ***	—
	p-value	< .001	< .001	< .001	—
	95% CI Upper	0.268	0.240	0.285	—
	95% CI Lower	0.118	0.088	0.136	—
	N	633	633	633	—

Note. * p < .05, ** p < .01, *** p < .001

References

[1] The jamovi project (2021). *jamovi*. (Version 1.6) [Computer Software]. Retrieved from https://www.jamovi.org.

[2] R Core Team (2020). *R: A Language and environment for statistical computing*. (Version 4.0) [Computer software]. Retrieved from https://cran.r-project.org. (R packages retrieved from MRAN snapshot 2020-08-24).
